# Pathophysiological Mechanisms Underlying Sarcopenia and Sarcopenic Obesity: A Systematic Review and Meta-Analysis of Biomarker Evidence

**DOI:** 10.3390/ijms26115113

**Published:** 2025-05-26

**Authors:** Zhiyuan Feng, Jiayue Xia, Junhui Yu, Jiongnan Wang, Shiyu Yin, Jingyi Yang, Tianyu Wu, Zhenzhen Zhang, Wei Yan, Shaokang Wang, Guiju Sun

**Affiliations:** 1 Key Laboratory of Environmental Medicine and Engineering of Ministry of Education, School of Public Health, Southeast University, Nanjing 210009, China; 15832238185@163.com (Z.F.); 230229010@seu.edu.cn (J.X.); jshmyjh@126.com (J.Y.); 230239098@seu.edu.cn (J.W.); yinshiyu@seu.edu.cn (S.Y.); jingyiyang@seu.edu.cn (J.Y.); tianyu_w@seu.edu.cn (T.W.); zzyw120530@163.com (Z.Z.); yan03260326@163.com (W.Y.); 2 Department of Nutrition and Food Hygiene, School of Public Health, Southeast University, Nanjing 210009, China

**Keywords:** sarcopenia, sarcopenic obesity, biomarkers, inflammation, hematologic markers

## Abstract

Sarcopenia and sarcopenic obesity (SO) represent significant age-related muscular disorders. Their specific biomarkers and pathophysiological mechanisms remain insufficiently elucidated. This study aims to identify differential and shared biomarkers between these conditions to reveal distinct pathophysiological processes, providing a foundation for precision diagnostics and targeted interventions. We conducted a systematic review and meta-analysis of studies examining biomarkers related to sarcopenia and SO in adults aged 45 and older. Electronic and manual searches were performed in PubMed, Web of Science, Cochrane Library, and Embase up to December 2024. The quality of each study was assessed using the National Institutes of Health Quality Assessment Tool. Meta-analysis was performed when at least three studies investigated the same biomarkers in frailty and sarcopenia, calculating the pooled effect size based on the standard mean difference using a random effects model. In total, 80 studies (64 on sarcopenia and 16 on SO) were included, encompassing 36,680 older adults (aged 45 and above) from 16 countries with varying levels of development. Participants were categorized based on their setting, age, and gender distribution. Sarcopenia is characterized by lower serum triglycerides and stable HDL/LDL ratios, while SO presents with higher triglycerides and disrupted cholesterol correlation, indicating distinct metabolic interactions. Analysis of inflammatory profiles revealed significantly elevated CRP levels in SO, with WBC as a specific marker, while TNF-α was associated with sarcopenia, suggesting a subtype-specific role of chronic inflammation. Vitamin D deficiency is prevalent in both conditions and may represent a potential therapeutic target. Subgroup analyses indicated an increased risk of muscle function decline in high-risk communities in developing regions, underscoring the urgent need for early intervention. A set of shared metabolic, hematologic, and inflammatory biomarkers was identified in sarcopenia and SO. These findings address a knowledge gap in biomarker research and highlight the distinct mechanisms involved in the development of both conditions. Developing biomarker-based diagnostic algorithms is essential for optimizing personalized treatment. Subgroup analyses have also identified high-risk populations, underscoring the need for early intervention.

## 1. Introduction

Aging is a complex process characterized by a gradual decline in organ function, disruption of homeostasis, reduced physiological reserves, and changes in body composition [1,2]. The skeletal muscle mass and function in older adults decline at rates of 0.5–1% and 2–3% per year with increasing age [3]. The aging population has led to an increase in the incidence of elderly diseases, disability rates, a decline in quality of life, and mortality rates. In 2016, sarcopenia was included in the International Classification of Diseases, 10th Revision, recognizing it as a medical condition [4]. Although there is considerable consensus on the theoretical framework of sarcopenia, clinical recognition is hindered by complex pathophysiology, heterogeneous phenotypic expressions, individual variability in severity, and the existence of multiple operational definitions [5,6].

With the increase in life expectancy, the proportion of older adults with high body fat and/or low muscle function and quality is gradually rising [7,8]. However, sarcopenia and sarcopenic obesity (SO) are closely related conditions that are still rarely identified and untreated in clinical practice [9,10]. Currently, the estimated prevalence of sarcopenia ranges from 6.2% to 35.3%, characterized by decreased muscle function and quality [11]. SO is a common elderly combination of excess body weight and loss of muscle quality and strength, with a global prevalence of 11% (95% CI, 10–13%) [12]. Both sarcopenia and SO may serve as risk factors for other chronic inflammation-based complications, such as anabolic resistance (AR), insulin resistance (IR), cardiovascular disease, and diabetes [13,14]. Therefore, the elderly population often has multiple comorbidities, making it difficult to clarify the specificity and sensitivity of certain biomarkers, and many potential biomarkers may serve as important factors in disease onset while also being results of disease progression.

The pathophysiological mechanisms of sarcopenia and SO are similar to those of other chronic elderly diseases, and the etiology of these diseases involves many biological characteristics of aging (such as genomic and epigenetic instability, inflammatory status, disruption of protein balance, mitochondrial dysfunction, nutritional signaling dysregulation, dyslipidemia, and changes in intercellular signaling) [15]. Consequently, multimodal biomarker analysis may be better suited than single-marker methods for elucidating the pathogenesis of these dynamic and complex diseases [16,17]. The fundamental principle of the multimarker paradigm is rooted in the concept of allostatic load, which refers to the cumulative biological burden imposed on cells and bodily systems during recurrent or chronic stress [18]. Therefore, biomarkers serve as internal phenotypes of physiological dysregulation, supporting the diagnosis and monitoring of specific conditions, as well as the validation of clinical decisions and intervention outcomes. Nevertheless, to the best of our knowledge, earlier reviews have not synthesized structured data to investigate the connections between diverse aging markers and the diagnosis of primary sarcopenia and SO.

This meta-analysis aims to examine the association between circulating biomarkers and incident risk of sarcopenia and SO, while clarifying their roles in disease mechanisms and guiding intervention strategies. Through analysis of biomarkers associated with two disease populations, we aim to identify potential shared signaling pathways underlying these conditions. Our work will address knowledge gaps in biomarker discovery for these diseases and establish a robust foundation for guiding future biomarker selection and clinical treatment regimen development in sarcopenia and SO research.

## 2. Methods

This systematic review, registered on PROSPERO (CRD420250653481), followed the Cochrane Handbook for Diagnostic Test Accuracy and adhered to the PRISMA guidelines for reporting (Supplementary S1) [19].

We performed a systematic review and meta-analysis to investigate the co-expression of biomarkers in sarcopenia and SO. We selected biomarkers from serum, plasma, saliva, urine, feces, and cerebrospinal fluid, focusing on observational studies. Candidates were identified as molecules reported in at least three studies on sarcopenia and three on SO and were included in our analyses.

### 2.1. Search Strategy and Selection Criteria

Research published on or before 31 December 2024, was retrieved by two researchers (Z. Feng and J. Xia) from PubMed, Web of Science, Cochrane Library, and Embase. The reference lists obtained were examined for inclusion in the review, and the initial search strategy utilized keywords, MeSH terms, and free-text terms (such as sarcopenic obesity, sarcopenia, biomarkers). The retrieval strategy is detailed in Supplementary S2, and references of retrieved studies were also screened to identify potentially eligible studies.

### 2.2. Inclusion and Exclusion Criteria

The inclusion criteria are as follows: (a) Diagnosis of sarcopenia based on the presence of muscle wasting plus reduced muscle strength and/or physical dysfunction [20,21]; (b) Participants aged 45 years or older; (c) Studies published in Chinese or English; (d) Availability of means and standard deviations (SD) for biomarker levels in cases (i.e., sarcopenia/SO) and non-cases (i.e., non-sarcopenia); (e) Observational studies, including cross-sectional, case-control, and longitudinal studies, that investigated the association between aging and/or sarcopenia with biomarker levels in elderly individuals as a primary or secondary outcome. Studies that did not report diagnostic criteria for sarcopenia or defined it solely by skeletal muscle mass, as well as animal studies, editorials, reviews, meta-analyses, conference abstracts, comments, and research protocols, were excluded, along with duplicate publications.

### 2.3. Data Extraction and Quality Assessment

Two researchers (Z. Feng and J. Xia) screened article titles and abstracts, consulting the full text when necessary. They utilized a standardized coding form to extract variables and sought the input of a third researcher (J. Yu) to resolve any disagreements. The reporting quality of each study was assessed by the two researchers (Z. Feng and J. Xia) using the National Institutes of Health (NIH) quality assessment tools for observational cohort and cross-sectional studies, as well as the NIH quality assessment tool for case-control studies (NIH, 2014). This tool includes 12–14 questions aimed at evaluating various aspects. Certain questions were excluded because they were not relevant to the specific types of studies. Ultimately, each study was categorized based on scores into three levels: poor (0–4), fair (5–6), and good (7–8), with an inter-rater reliability of 99% for the quality assessments.

### 2.4. Statistical Analysis

Meta-analysis was performed using STATA 17 (StataCorp, College Station, TX, USA). Summary analysis was performed when at least three studies assessed the same biomarker in two contexts (comparing frailty versus robustness and sarcopenia versus non-sarcopenia). Effect sizes (ES) were quantified as mean values and standard deviations (SD). If the authors did not report mean and SD data, SD was calculated following Cochrane guidelines [22], specifically deriving SD from confidence intervals or standard errors, with means converted from medians. Data reported graphically for measures of central tendency and dispersion were extracted using Plot Digitizer 2.4.4 software [23]. When multiple studies referenced the same database, a single pairwise comparison was created according to the Cochrane group formula. Considering the variations in techniques used for biomarker quantification (e.g., ELISA, multiplex assays) and/or biological samples (e.g., serum, plasma), mixed ES was calculated based on standardized mean differences (SMD). Due to variability in sample characteristics, study designs, and the assessment tools for frailty and sarcopenia, a random-effects model was used to calculate the pooled ES. Sensitivity analysis was performed utilizing a stratified approach based on binary data, with strata defined by age (<75 years and ≥75 years), setting (community, hospital, and long-term care), country development level (developed countries and developing countries), and sex ratio (male/female <1 or ≥1).

## 3. Results

### 3.1. Search Results

**Figure 1:**
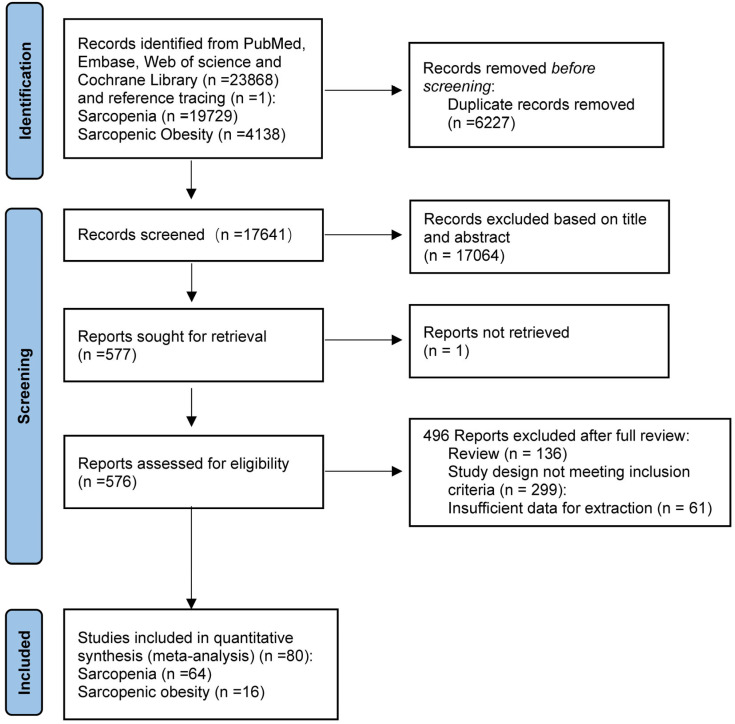
Study selection process based on PRISMA guidelines. PRISMA: Preferred Reporting Items for Systematic Reviews and Meta-Analysis.

Figure 1 presents the PRISMA flowchart outlining the various stages of the systematic review. A total of 23868 records were identified through database searches and 1 record through reference tracing. Of these, 6227 records were removed as duplicates, and 17,064 articles were excluded based on title and abstract, leaving 576 articles for eligibility assessment. Following the elimination of 496 studies according to the inclusion criteria, 80 studies were included in the qualitative analysis, which included 64 studies on sarcopenia and 16 on SO.

### 3.2. Trial Characteristics

We first performed a meta-analysis of data measuring biomarkers related to sarcopenia and SO (Supplementary S3 and Supplementary S4). Among the 80 eligible studies, 64 reported data on sarcopenia [24,25,26,27,28,29,30,31,32,33,34,35,36,37,38,39,40,41,42,43,44,45,46,47,48,49,50,51,52,53,54,55,56,57,58,59,60,61,62,63,64,65,66,67,68,69,70,71,72,73,74,75,76,77,78,79,80,81,82,83,84,85,86,87], and 16 on SO [88,89,90,91,92,93,94,95,96,97,98,99,100,101,102,103] were included in the qualitative analysis. The identified studies investigated molecules related to inflammation, metabolism, hematology, and hormones, and reported accordingly. A total of 23,534 community-dwelling and hospitalized older adults (aged 45 and above) with sarcopenia were recruited from Australia, India, Austria, Brazil, China, Germany, Italy, Japan, South Korea, Spain, China (Taiwan), Singapore, Turkey, and Saudi Arabia. The average age of the study participants ranged from 45 to 95 years. Studies investigating SO included 13,146 community-dwelling, hospitalized, and institutionalized older adults from Italy, Brazil, South Korea, China, India, Japan, Iran, Australia, and China (Taiwan), with an average age ranging from 53 to 86 years. According to the study protocol, all investigations defined sarcopenia and SO based on the coexistence of muscle atrophy and dynapenia and/or physical frailty.

### 3.3. Methodology Quality Appraisal

In this meta-analysis, we performed a systematic methodological quality evaluation of the included studies (Supplementary S5). The NIH tool was used for assessment, with a scoring range of 0 to 10 points. Based on the scoring results, among the 60 included studies, 17 were rated as high quality (7–8 points), 59 as moderate quality (5–6 points), and none as low quality (≤4 points). All included studies clearly articulated the research questions and meticulously described the characteristics of the study population and the inclusion criteria (Questions 1 and 2) and employed effective and reliable tools to assess sarcopenia or SO (Question 11). Among all the studies, only one did not recruit participants from the same or similar populations and was evaluated according to the same inclusion criteria (Question 4). A total of 71 studies utilized valid and reliable tools and provided clear descriptions for the assessment of biomarkers (Question 9), whereas 61 studies adjusted the results for potential confounding factors (Question 14). Only 19 studies provided a rational justification for the sample size (Question 5). 6 studies reported whether the participation rate of eligible candidates was at least 50% (Question 3). Only five studies examined whether the variables were evaluated by blinded assessors (Question 12).

Two studies were case-control studies, and all investigations clearly stated the research questions (Question 1) and specified and defined the study population (Question 2). Controls were recruited from the same or similar case groups (Question 4), and the same eligibility criteria were used to select cases and controls (Question 5), clearly defining and distinguishing the two groups (Question 6) and using concurrent controls (Question 8). They employed valid and reliable tools to assess frailty or sarcopenia (Question 10), while no study demonstrated the rationale for the sample size (Question 3), adjusted data for potential confounding factors (Question 12), or described whether assessors were blinded to participants’ case or control status (Question 11). Overall, while most studies adhered to certain standards in writing and design, deficiencies in sample size and blinding assessments may affect the credibility of the results. Therefore, these results should be interpreted with caution in subsequent analyses.

### 3.4. Shared Biomarkers Between Sarcopenia and SO

**Figure 2:**
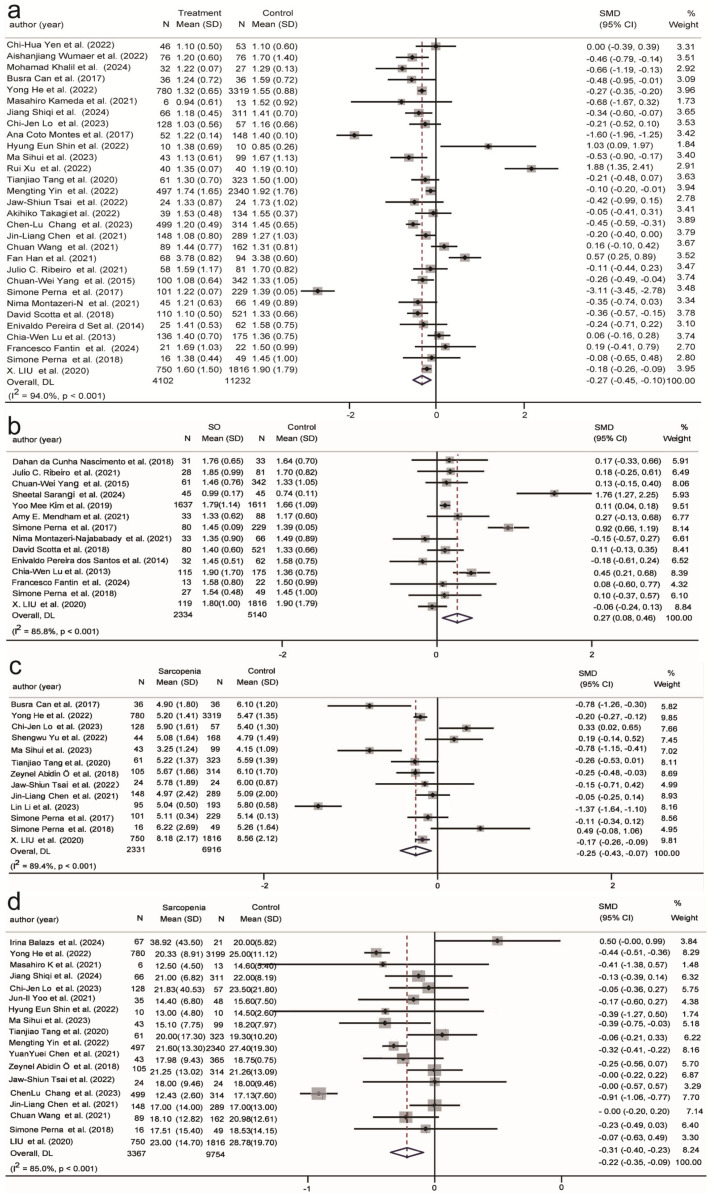
Association of metabolic biomarkers with sarcopenia and SO. (**a**) Association between serum triglycerides and sarcopenia; (**b**) Association between serum triglycerides and SO; (**c**) Association between uric acid and sarcopenia; (**d**) Association between ALT and sarcopenia. SMD: Standardized Mean Difference.

**Figure 3:**
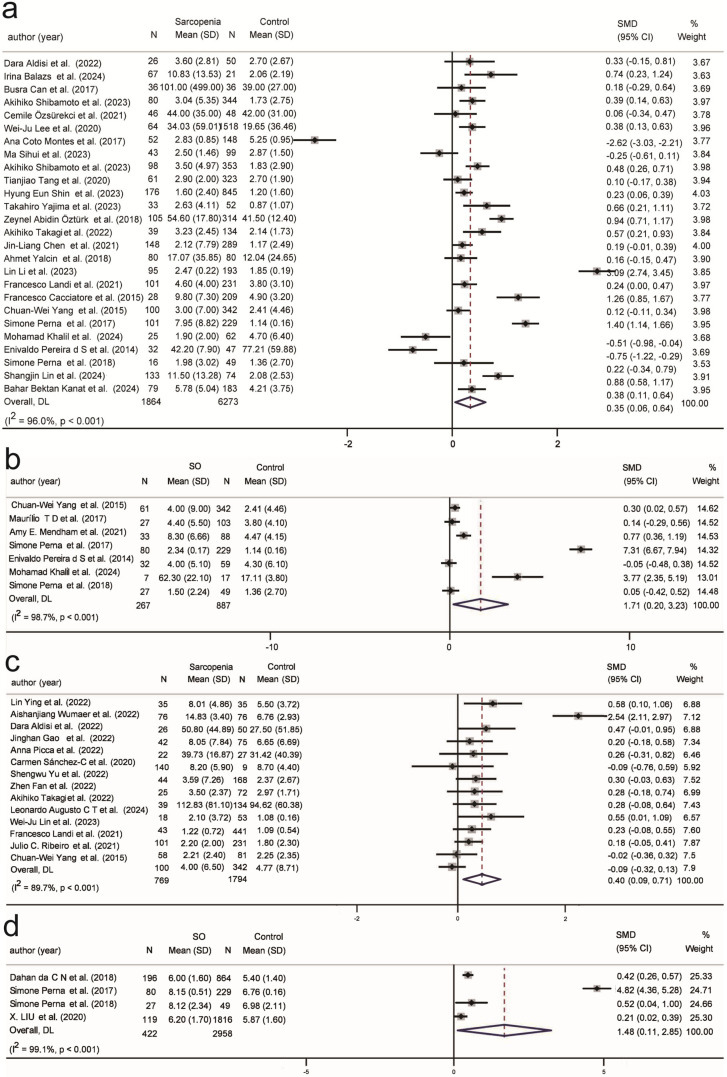
Association of inflammation with sarcopenia and SO. (**a**) Association between CRP and sarcopenia; (**b**) Association between CRP and SO; (**c**) Association between TNF-α and sarcopenia; (**d**) Association between WBC and SO. SMD: Standardized Mean Difference.

**Figure 4:**
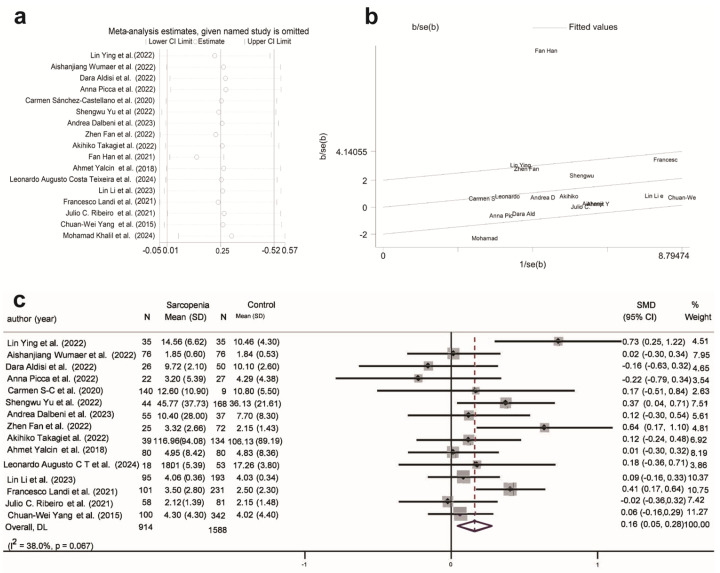
Refined analysis of IL-6 and sarcopenia excluding high-bias Studies. (**a**) Leave-one-out sensitivity analysis of IL-6 in sarcopenia; (**b**) Galbraith test for heterogeneity in IL-6 and sarcopenia; (**c**) Refined analysis of IL-6 and sarcopenia excluding high-bias Studies. SMD: Standardized Mean Difference.

**Figure 5:**
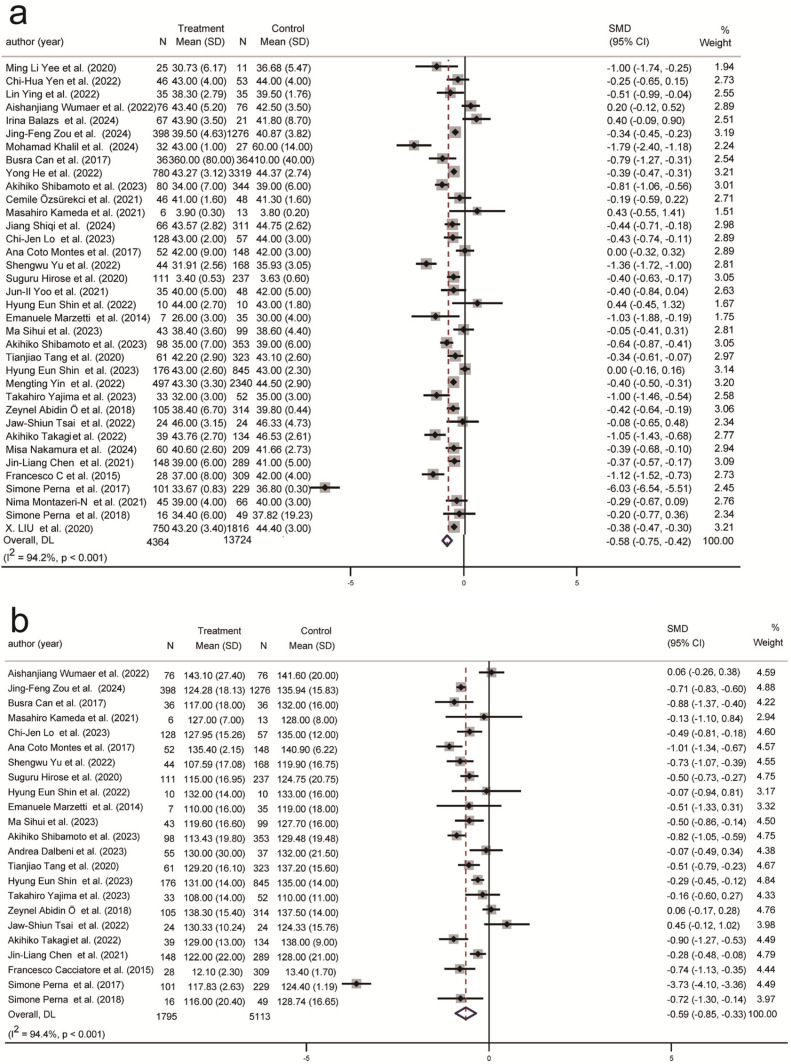
Association of hematological and hormonal biomarkers with sarcopenia and SO. (**a**). Association between albumin and sarcopenia; (**b**) Association between hemoglobin and sarcopenia; SMD: Standardized Mean Difference.

**Figure 6:**
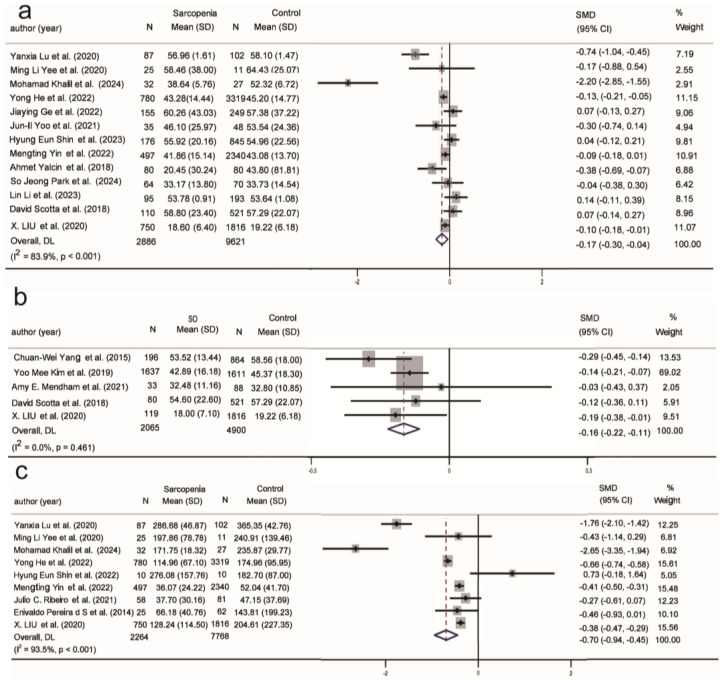
Association of hormonal biomarkers with sarcopenia and SO. (**a**) Association between 25(OH)D and sarcopenia; (**b**) Association between 25(OH)D and SO; (**c**) Association between insulin and sarcopenia. SMD: Standardized Mean Difference.

Table 1 presents the biomarkers of frailty and sarcopenia measured in at least three studies, while Figure 2, Figure 3, Figure 4, Figure 5 and Figure 6 illustrate the forest plot results of biomarkers significantly correlated with sarcopenia or SO. The supplementary figures in Supplementary S6 and Supplementary S7 present the forest plot results for biomarkers from the subgroup analyses of sarcopenia or SO. Metabolic, hematologic, and inflammatory biomarkers were identified.

#### 3.4.1. Metabolic Biomarkers in Sarcopenia and SO

Overall, triglycerides show opposite correlations in populations with sarcopenia and SO, with triglycerides negatively correlated with sarcopenia (SMD = −0.27, 95% CI (−0.45, 0.10), K = 30, N = 15,282, *I*^2^ = 94.0%) (Figure 2a). In participants aged <75 years, in community populations, in developing countries, and among males, serum triglyceride levels were negatively associated with sarcopenia. In participants aged >75 years, this relationship was not significant in hospitalized individuals, those in developed countries, and females (Figure S1). Conversely, triglycerides showed a positive correlation with SO (SMD = 0.27, 95% CI (0.08, 0.46), K = 14, N = 7474, *I*^2^ = 85.8%) (Figure 2b). SO populations showed a positive association with triglycerides in those aged <75 years, in community populations, and among males (Figure S2).

The sarcopenia population is positively correlated with high-density lipoprotein (SMD = 0.15, 95% CI (−0.03, 0.34), K = 26, N = 15,015, *I*^2^ = 94.6%) and low-density lipoprotein (SMD = 0.14, 95% CI (−0.02, 0.30), K = 21, N = 13,925, *I*^2^ = 91.5%) (Figure S3a and Figure S4a). In community populations and among females, HDL showed a positive correlation with sarcopenia (Figure S3). However, LDL showed a positive correlation with sarcopenia only in male participants (Figure S4). There was no correlation between the SO population and HDL (Figure S5), and only the SO population in developing countries showed a positive correlation with LDL (Figure S6).

Glucose (SMD = −0.10, 95% CI (−0.25, 0.04), K = 12, N = 14,288, *I*^2^ = 87.1%) (Figure S7) and cholesterol (SMD = −0.13, 95% CI (−0.32, 0.07), K = 33, N = 15,862, *I*^2^ = 95.8%) (Figure S8) did not show any correlation with the population of sarcopenia. Similarly, glucose (SMD = 0.00, 95% CI (−0.19, 0.19), K = 11, N = 6619, *I*^2^ = 77.1%) (Figure S9) and cholesterol (SMD = −0.07, 95% CI (−0.37, 0.22), K = 12, N = 6722, *I*^2^ = 93.0%) (Figure S10) also did not show any correlation with the SO population. However, glucose was positively correlated with the SO population in participants with an average age < 75 years, in community populations, and in developing countries, while it was negatively correlated with SO populations aged >75 years.

Figure 2c shows a significant negative correlation between the sarcopenia population and uric acid (SMD = −0.25, 95% CI (−0.43, −0.07), K = 13, N = 9247, *I*^2^ = 89.4%). Uric acid is negatively correlated with the sarcopenia population in participants aged <75 years, in community populations, and in developing countries. In contrast, in participants over 75 years, hospitalized individuals, and those in developed countries, this relationship was not apparent (Figure S11). Conversely, among the SO population under 75 years old, in community settings and developing countries, there was a positive correlation with uric acid (Figure S12).

Compared to the normal population, ALT is negatively correlated with the sarcopenia population (SMD = −0.22, 95% CI (−0.35, −0.09), K = 18, N = 13,121, *I*^2^ = 85.0%) (Figure 2d), and is not correlated with SO (Figure S13). ALT is negatively correlated in the sarcopenia population within community settings and in developing countries (Figure S14).

Creatinine and AST did not show any correlation with the sarcopenia and SO populations in the study (Figures S15–S18).

#### 3.4.2. Inflammation Biomarkers in Sarcopenia and SO

Among the four immune activation biomarkers, CRP showed a positive correlation with sarcopenia (SMD = 0.35, 95% CI (0.06, 0.64), K = 26, N = 8137, *I*^2^ = 96.0%) and the SO population (SMD = 1.71, 95% CI (0.2, 3.23), K = 7, N = 1154, *I*^2^ = 98.7%) compared to individuals with normal muscle function (Figure 3a,b). In hospitalized participants with an average age < 75 years, CRP exhibited a more pronounced positive correlation with sarcopenia (Figure S19), while CRP did not show any correlation with the SO subgroup (Figure S20).

Compared to the normal population and SO (Figure S21), TNF-α is significantly positively correlated with sarcopenia (SMD = 0.40, 95% CI (0.09, 0.71), K = 14, N = 2563, *I*^2^ = 89.7%) (Figure 3c). TNF-α is positively correlated with sarcopenia regardless of age and the level of development of the country. It is positively correlated with sarcopenia in hospitalized individuals and in males, but no association was observed in studies with a higher proportion of females in community settings (Figure S22).

Compared to the normal population and sarcopenia (Figure S24), WBC is significantly positively correlated with SO (SMD = 1.48, 95% CI (0.11, 2.85), K = 4, N = 3380, *I*^2^ = 99.1%) (Figure 3d). WBC is positively correlated with SO of age < 75 years, in LDCs, and in community-dwelling populations (Figure S25).

Interestingly, contrary to previous studies that showed a significant positive correlation between IL-6 and sarcopenia [104,105,106], this study did not identify a significant positive correlation between IL-6 (SMD = 0.25, 95% CI (−0.01, 0.52), K = 17, N = 2710, *I*^2^ = 88.7%) and sarcopenia, with a substantial positive correlation evident only in developing countries (Figure S23). Further analysis of the included studies, using a leave-one-out approach and Galbraith heterogeneity tests, revealed that Fan Han et al. and Mohamad Khalil et al. had a substantial impact on the results (Figure 4a,b) [75,88]. Excluding these two studies from the meta-analysis significantly reduced heterogeneity (*I*^2^ = 38.0%), and a significant positive correlation between IL-6 and sarcopenia was found (SMD = 0.16, 95% CI (0.05, 0.28), K = 15, N = 2502, *I*^2^ = 38%) (Figure 4c). The subgroup analysis results in Table 2 indicated that IL-6 was positively correlated with sarcopenia in populations under 75 years old, in hospitalized individuals, and in developing countries with a larger proportion of females (Figure S26). However, no information was found regarding the association between IL-6 and SO (Figure S27).

#### 3.4.3. Hematological Biomarkers in Sarcopenia and SO

Two hematological biomarkers, albumin (SMD = −0.58, 95% CI (−0.75, −0.42), K = 37, N = 18,178, *I*^2^ =94.2%) (Figure 5a) and hemoglobin (SMD = −0.59, 95% CI (−0.85, −0.33), K = 23, N = 6908, *I*^2^ =94.4%) (Figure 5b), were significantly negatively correlated with sarcopenia across all four classifications (Figure S28 and Figure S29). However, albumin was positively correlated with SO only in those under 75 years old, in community populations, and in developing countries (Figure S30). There was no significant correlation observed between SO and hemoglobin (Figure S31).

#### 3.4.4. Hormonal Biomarkers in Sarcopenia and SO

Research indicates that serum 25(OH)D levels are significantly lower in individuals with sarcopenia. Recently, vitamin D has been suggested as a modulator of skeletal muscle function, enhancing mitochondrial ATP production and alleviating oxidative stress through the overexpression of vitamin D receptors (VDR) in skeletal muscle stem cells [107,108]. In this study, compared to the normal population, 25(OH)D levels showed a significant negative correlation in the sarcopenia (SMD = −0.17, 95% CI (−0.30, −0.04), K = 13, N = 12,507, *I*^2^ = 83.9%) and SO populations (SMD = −0.16, 95% CI (−0.22, −0.11), K = 5, N = 6965, *I*^2^ = 0.0%) (Figure 6a,b). In the subgroup analysis, serum 25(OH)D was negatively correlated with sarcopenia in populations aged over 75 years, community settings, and among males in developing countries (Figure S32). Regardless of country development level and gender, 25(OH)D showed a significant negative correlation with SO (Figure S33). In contrast to individuals with sarcopenia alone, those aged under 75 years in the SO population exhibited a negative correlation with 25(OH)D. However, there is currently insufficient research analyzing the correlation between the SO population and 25(OH)D across different settings.

Insulin balance plays an important role in maintaining muscle mass and function, with both insulin resistance and insulin deficiency promoting the occurrence and progression of sarcopenia. Figure 6c shows a significant negative correlation between insulin levels and sarcopenia (SMD = −0.70, 95% CI (−0.94, −0.45), K = 9, N = 10,032, *I*^2^ = 93.5%). Among the nine studies, only the research by Hyung Eun Shin et al. (2022) [54] showed reduced insulin levels in individuals with sarcopenia. In the subgroup analysis, a negative correlation was found in the sarcopenia population aged <75 years, community populations, and those in developing countries with a higher proportion of males (Figure S34). No correlation was observed between insulin levels and the SO population (Figure S35).

### 3.5. Publication Bias

As shown in Supplementary S8 (Figures S36–S41), a funnel plot was systematically employed to assess potential publication bias among the included studies. The plot demonstrated a symmetrical distribution, with no evident signs of small-study effects or other biases. This observation strengthens the robustness and credibility of our findings.

## 4. Discussion

### 4.1. Principal Findings and Comparison with Previous Studies

This systematic review provides the first comprehensive analysis of the biomarker profiles associated with sarcopenia and SO, highlighting the complex similarities and pathophysiological differences in lipid metabolism, inflammatory markers, and endocrine regulation. The research identified a key differentiation in lipid metabolism: sarcopenia is characterized by a distinctive decrease in triglycerides, while SO exhibits an unusual increase in triglycerides and a disappearance of cholesterol correlation, indicating fundamental differences in the fat–muscle metabolic interaction. Classification-CRP is significantly elevated in SO, with WBC serving as a specific marker, while the selective association of TNF-α with sarcopenia suggests the existence of subtype-specific pathways driving chronic inflammation. At the endocrine level, the shared feature of vitamin D deficiency suggests common therapeutic targets, whereas the unique insulin resistance in sarcopenia, along with the negative correlation of both uric acid and ALT with muscle mass, indicates specific mechanisms of imbalance in muscle protein homeostasis. These findings characterize sarcopenia at the molecular level as a syndrome of protein degradation and chronic inflammation, whereas SO is classified as a fat-mediated metabolic inflammatory entity, which offers a translational medicine framework for biomarker-based classification and treatment.

Sarcopenia and SO share commonalities as well as significant differences in pathophysiological characteristics. The most notable difference lies in lipid metabolism—triglycerides exhibit completely opposite correlations (sarcopenia: SMD = −0.27, 95% CI [−0.43, −0.10]; SO: SMD = 0.27, 95% CI [0.08, 0.46]). A decrease in muscle mass may lead to impaired lipid oxidation capacity, and the body compensates by suppressing hepatic very low-density lipoprotein (VLDL) secretion to reduce the accumulation of systemic triglycerides [109]. Conversely, in SO, insulin resistance and chronic inflammation driven by visceral fat accumulation may play a dominant role, leading to enhanced lipolysis and excessive production of hepatic VLDL [110].

Simultaneously aligning with the latest evidence of mitochondrial heterogeneity in adipose tissue, muscle fat infiltration in sarcopenia may stem from the selective atrophy of beige fat mitochondria [111], while SO exhibits typical white fat dysfunction coupled with enhanced FGF21 resistance, leading to a disorder in the capacity of adipose tissue to store and metabolize triglycerides [112].

Consistent with previous research findings, serum UA levels are positively correlated with muscle strength in the population [113]. Both conditions exhibit a metabolic disconnection with blood glucose and cholesterol levels, although SO shows age-related fluctuations in blood glucose, which are not observed in sarcopenia.

The differences in inflammatory characteristics between sarcopenia and SO highlight the fundamentally distinct pathophysiological mechanisms driving these two conditions. While elevated CRP levels are prevalent in both conditions, the marked increase observed in SO indicates a dominant inflammatory role of the fat–liver axis. Visceral fat tissue drives the production of CRP through non-IL-6-dependent pathways, directly activating CRP transcription in hepatocytes via the NF-κB pathway [113,114]. Furthermore, free fatty acids released from visceral fat increase hepatocyte sensitivity to low levels of IL-6 via the TLR4-MyD88 pathway, resulting in a “synergistic amplification effect” on CRP production [115].

The specific increase in TNF-α in sarcopenia and the refined IL-6 levels after excluding studies with significant heterogeneity delineate the muscle-autonomous inflammatory pathway [116]. Muscle wasting triggers mitochondrial dysfunction, causing the release of mitochondrial DNA (mtDNA) and activation of the NLRP3 inflammasome, thereby promoting excessive TNF-α production. TNF-α, a crucial pro-inflammatory cytokine, exacerbates muscle atrophy by activating the NF-κB and JNK signaling pathways, which promote muscle protein degradation and suppress muscle synthesis [117,118]. However, in the context of chronic inflammation or muscle atrophy, IL-6 inhibits the differentiation capacity of muscle stem cells through SOCS3 feedback and, in synergy with other inflammatory mediators (such as TNF-α), blunts muscle anabolism and disrupts energy homeostasis, ultimately leading to muscle catabolism and atrophy [119]. The cooperative effects of these inflammatory mediators play a pivotal role in the pathology of sarcopenia. The interaction between IL-6 and TNF-α not only embodies the intrinsic phenotype of “inflammaging” but also highlights the heightened sensitivity of muscle to inflammatory signals during the aging process [120]. While age-related elevation in IL-6 levels may not independently trigger muscle atrophy, its synergistic interplay with TNF-α fosters an environment that promotes muscle wasting [119]. Furthermore, in chronic inflammation, the liver responds by producing acute-phase reactants (such as CRP) while suppressing the synthesis of other proteins (such as albumin), further exacerbating muscle protein degradation and functional loss. This complex interplay between inflammation and muscle metabolism provides new insights into the pathological mechanisms of sarcopenia and highlights the potential value of targeting inflammatory pathways to delay or reverse muscle atrophy.

The unique leukocytosis in SO is closely related to the adipose-immune cell interaction network [121]. The expansion of visceral fat triggers inflammatory responses, recruiting monocytes to infiltrate the adipose tissue. These monocytes can differentiate into dendritic cells that secrete cytokines such as IL-23, thereby promoting the extramedullary expansion of neutrophils [122]. However, in sarcopenia, the absence of fat-driven immune cell recruitment results in systemic white blood cell counts remaining within normal limits.

Hematological parameters indicate that sarcopenia patients commonly exhibit reduced albumin and hemoglobin levels, potentially reflecting alterations in muscle–liver metabolic interactions. Muscle atrophy may lead to branched-chain amino acid metabolic dysregulation, which is closely associated with hepatic albumin synthesis and hematopoietic function [123]. In SO patients, albumin levels tend to remain relatively stable, possibly due to metabolic interactions between adipose tissue and the liver. Studies demonstrate that bioactive substances secreted by adipose tissue, such as leptin, participate in metabolic regulation through signaling pathways including JAK2/STAT3, while inflammatory factors like TNF-α may suppress the gene expression of various proteins [124,125].

This study reveals that vitamin D deficiency is a shared feature of sarcopenia and SO, suggesting that both conditions involve endocrine dysregulation and impaired skeletal muscle mitochondrial function. Studies indicate that the vitamin D receptor (VDR) directly modulates mitochondrial oxidative phosphorylation capacity in muscle, and vitamin D deficiency markedly decreases the activity of mitochondrial complex I and inhibits critical genes involved in mitochondrial biogenesis. This compromised mitochondrial function results in diminished energy metabolism efficiency, characterized by reduced overall energy expenditure, further aggravating muscle wasting and fat deposition [126]. Notably, in muscle-specific VDR knockout mouse models, mitochondrial density is reduced by 20–30%, and the activity of the key fatty acid oxidation enzyme HAD is decreased by 40%, indicating that the VDR signaling pathway plays a central role in maintaining muscle metabolic homeostasis [127]. Clinical intervention trials further confirm that vitamin D supplementation significantly increases appendicular skeletal muscle mass and grip strength in elderly subjects, suggesting that improving vitamin D status may break the vicious cycle of sarcopenia and SO by restoring mitochondrial oxidative capacity [128,129]. The effects of vitamin D intervention still require cautious interpretation. Uusi-Rasi et al.’s study on women aged 70–80 found that exercise alone improved lower limb muscle strength and physical function, but vitamin D did not enhance the functional benefits of exercise, which may be related to the participants’ relatively high baseline serum 25(OH)D3 levels [130]. Notably, the international clinical practice guidelines for sarcopenia do not recommend vitamin D supplementation for elderly individuals with sarcopenia due to insufficient evidence [130]. These findings indicate that the clinical efficacy of vitamin D may be affected by baseline levels, intervention dose, and gender variations, necessitating further stratified studies to determine its specific applicable population.

Overall, these findings reveal that while both conditions involve endocrine dysregulation, sarcopenia is more dependent on insulin signaling and the imbalance of related metabolic networks, whereas SO primarily manifests as disruptions in hormonal signaling and metabolic pathways due to abnormal adipose tissue. These findings link sarcopenia mainly to protein breakdown and chronic inflammation, while SO exhibits lipid-driven metabolic inflammation and enhanced immune activation. The shared pattern of vitamin D deficiency suggests potential common therapeutic targets, while distinct biomarker profiles support the development of different diagnostic algorithms for these clinically overlapping but biologically distinct entities.

Our stratified analysis reveals key effect modification patterns with significant pathophysiological and clinical implications. This study extracted 17 indicators, of which 11 showed significant associations with sarcopenia (10 indicators) or SO (4 indicators). In subgroup analyses of these 12 indicators, age < 75 years (10 indicators), community living environment (10 indicators), and developing countries (11 indicators) were significantly related to disease occurrence. Among participants aged <75 years, living in community environments, and from developing countries, biochemical and inflammatory indicators exhibited the most significant associations with sarcopenia and SO. Although age, environment, and economic development level are not the sole determinants, these grouping characteristics may collectively shape the mechanisms underlying muscle decline and body composition abnormalities, warranting further exploration.

In individuals under 75 years of age, muscle mass has not yet undergone the accelerated loss seen in older age groups, making them more sensitive to metabolic and inflammatory abnormalities [131]. In this population, vitamin D deficiency and elevated inflammatory factors often manifest more directly as a “dual burden” of skeletal muscle dysfunction and fat accumulation. After the age of 75, the body typically presents with a more complex spectrum of chronic diseases, such as cardiovascular and cerebrovascular disorders, moderate to severe glucocorticoid use, or other comorbidities, which may obscure the association between single indicators like vitamin D status or immune factors and muscle decline [132].

Community populations typically have relatively autonomous lifestyles and a broader range of activities, and their daily behavioral patterns (such as outdoor exercise and dietary structure) directly influence vitamin D synthesis and energy metabolism [133]. Comparatively, hospitalized individuals are affected by illnesses and treatment plans (particularly drugs such as glucocorticoids), leading to more intricate variations in serological markers, which complicate the identification of the specific metabolic or inflammatory signals driving muscle loss [134]. Simultaneously, attention must be paid to potential confounders in the community population, such as inadequate nutritional intake or gender and physical labor disparities, and future research should incorporate more diverse cohorts for further validation.

In developing countries, disease biomarkers such as vitamin D deficiency, altered glucose and lipid metabolism, and inflammation are more likely to be exacerbated by factors such as air pollution, monotonous dietary patterns, and the lack of fortified foods [135]. Research by Yang et al. suggests that in environments lacking systematic nutritional interventions, individuals are more likely to experience impaired mitochondrial function in skeletal muscle, leading to the concurrent pathological features of decreased muscle strength and fat accumulation [136]. The level of poverty and insufficient access to medical resources increase the risk of the coexistence of obesity and sarcopenia [137,138]. Conversely, developed countries typically have more comprehensive nutritional supplementation and health screening mechanisms, which relatively reduce the severity of vitamin D and other nutrient deficiencies, thereby diminishing the statistical association of single indicators in population cohorts [137].

Overall, the three stratifications of age <75 years, community environment, and developing country background collectively imply more actionable and observable external factors: malnutrition, physical inactivity, or insufficient early intervention, all of which can directly affect inflammatory markers, skeletal muscle mitochondrial function, and body composition changes [139,140].

### 4.2. Implications of Study and Limitations

Accurate management of sarcopenia and SO depends on a comprehensive understanding of biomarker profiles. This study systematically elucidates the similarities and differences between the two conditions in lipid metabolism, inflammatory markers, and endocrine regulatory biomarker profiles, offering a scientific foundation for tailored interventions. The study shows that although vitamin D deficiency is a common feature of both conditions, the efficacy of supplementation is influenced by baseline levels, intervention dosage, and gender differences, suggesting the need for careful evaluation in clinical practice. The differentiation of sarcopenia and SO in inflammatory profiles and metabolic regulation provides direction for targeted therapy. Sarcopenia requires attention to branched-chain amino acid supplementation, while SO necessitates reshaping adipose-liver metabolic flow through PPAR γ agonists.

However, the current study still has limitations, with the evidence quality for most biomarkers being low and their clinical significance not yet fully clarified. Based on the biomarker characteristics revealed in this study, further research will be conducted to achieve early screening and precise management of sarcopenia and SO, develop diagnostic classification algorithms, and formulate targeted public health policies and nutritional interventions. Furthermore, the limited number of studies meeting inclusion criteria for certain biomarkers, the heterogeneity of analyzed biomolecules, and restricted access to raw data hindered more in-depth statistical analyses.

## 5. Conclusions

In conclusion, vitamin D deficiency merits attention as a common factor, while the distinct lipid profiles and inflammatory response patterns between sarcopenia and SO have provided critical biomarker combinations that reveal underlying pathophysiological differences. Specific biomarkers contribute to an enhanced understanding of the molecular mechanisms governing disease onset and progression, offering actionable indicators for clinical practice. Stratified analysis further emphasizes the influence of age, lifestyle, and geographical factors on disease risk and progression, highlighting the importance of early intervention and targeted public health policies. Despite limitations in the observational study design and potential data heterogeneity restricting the quality of conclusions, our biomarker findings establish a platform for future randomized trials and longitudinal studies to refine precision management, mitigate muscle atrophy, and address metabolic dysfunction in the aging population.

## Figures and Tables

**Table 1:** Meta-analysis of differential biomarker associations in sarcopenia versus SO.

	Sarcopenia (n = 66)			Sarcopenic Obesity (n = 16)		
	SMD	95% CI	*I* ^2^	SMD	95% CI	*I* ^2^
Metabolic						
Glucose (9/11)	−0.10	−0.25, 0.04	87.1%	0.00	−0.19, 0.19	77.1%
Cholesterol (33/12)	−0.13	−0.32, 0.07	95.8%	−0.07	−0.37, 0.22	93.0%
Triglycerides (30/14)	−0.27	−0.45, −0.10	94.0%	0.27	0.08, 0.46	85.8%
HDL (26/12)	0.15	−0.03, 0.34	94.6%	−0.16	−0.41, 0.09	90.9%
LDL (25/9)	0.14	−0.02, 0.30	91.5%	0.10	−0.03, 0.22	41.4%
Creatinine (26/6)	0.04	−0.07, 0.15	77.7%	0.25	−0.60, 0.11	96.6%
Uric acid (13/4)	−0.25	−0.43, −0.07	89.4%	1.14	−0.35, 2.64	98.8%
ALT (18/3)	−0.22	−0.35, −0.09	85.0%	−0.02	−0.15, 0.10	38.8%
AST (17/3)	0.02	−0.04, 0.09	36.9%	0.05	−0.02, 0.12	3.0%
Inflammatory						
IL-6 (17/5)	0.25	−0.01, 0.52	88.7%	0.07	−0.24, 0.38	56.9%
TNF-α (14/3)	0.40	0.09, 0.71	89.7%	−0.10	−0.30, 0.10	0.0%
CRP (26/7)	0.35	0.06, 0.64	96.0%	1.71	0.20, 3.23	98.7%
WBC (18/4)	0.07	−0.19, 0.32	96.2%	1.48	0.11, 2.85	99.1%
Hematologic						
ALB (36/4)	−0.58	−0.75, −0.42	94.2%	−1.33	−3.25, 0.60	99.1%
HB (23/3)	−0.59	−0.85, −0.33	94.4%	0.09	−0.09, 0.27	32%
Hormonal						
25(OH)D (13/5)	−0.17	−0.30, −0.04	83.9%	−0.16	−0.22, −0.11	0.0%
Insulin (9/4)	−0.70	−0.94, −0.45	93.5%	0.09	−0.14, 0.32	75.7%

Abbreviations: ALB: albumin; CI: confidence interval; CRP: C-reactive protein; HDL: high-density lipoprotein; IL-6: interleukin 6; LDL: low-density lipoprotein; SMD: standard mean difference; TNF-α: tumor necrosis factor alpha; WBC: white blood cell; HB: hemoglobin.

**Table 2:** Meta-analysis results of IL-6 after excluding highly heterogeneous studies.

		SMD	95% CI	*I* ^2^
IL-6 (15)	Overall	0.16	0.05, 0.28	38.0%
Age	<75	0.14	0.00, 0.28	34.3%
	>75	0.21	−0.02, 0.43	44.4%
Setting	Hospitalized	0.24	0.07, 0.42	39.8%
	Community-dwelling	0.09	−0.08, 0.25	38.3%
DC/LDC	DC	0.20	−0.01, 0.40	24.3%
	LDC	0.16	0.01, 0.30	44.8%
Gender (male/female)	<1	0.13	−0.01, 0.27	24.7%
	>1	0.26	0.02, 0.50	61.8%

Abbreviations: CI: confidence interval; DC: developed country; LDCs: least-developed countries.

## Supplementary Materials

The supporting information can be downloaded at: https://www.mdpi.com/article/10.3390/ijms26115113/s1. Reference [141] is cited in the Supplementary Materials.
