# Supplementary material for: Pathophysiological Mechanisms Underlying Sarcopenia and Sarcopenic Obesity: A Systematic Review and Meta-Analysis of Biomarker Evidence
Source: Int J Mol Sci. 2025 May 26;26(11):5113. doi: 10.3390/ijms26115113 (PMC12154750; doi:10.3390/ijms26115113)
Supplement: Supplementary file 1 [file ijms-26-05113-s001.zip › ijms-3609159-supplementary.pdf]

Supplementary materials

Supplement to:

Pathophysiological Mechanisms Underlying Sarcopenia and Sarcopenic Obesity: A Systematic Review and Meta-Analysis of Biomarker Evidence

Supplementary S1 PRISMA Reporting Checklist

Section and Topic	Item #	Checklist item	Location where item is reported
**TITLE**			
Title	1	Identify the report as a systematic review.	Page 1
**ABSTRACT**			
Abstract	2	See the PRISMA 2020 for Abstracts checklist.	Page 2, 3
**INTRODUCTION**			
Rationale	3	Describe the rationale for the review in the context of existing knowledge.	Page 3, 4, 5
Objectives	4	Provide an explicit statement of the objective(s) or question(s) the review addresses.	Page 5, 6
**METHODS**			
Eligibility criteria	5	Specify the inclusion and exclusion criteria for the review and how studies were grouped for the syntheses.	Page 7
Information sources	6	Specify all databases, registers, websites, organisations, reference lists and other sources searched or consulted to identify studies. Specify the date when each source was last searched or consulted.	Page 6
Search strategy	7	Present the full search strategies for all databases, registers and websites, including any filters and limits used.	Page 6, 7
Selection process	8	Specify the methods used to decide whether a study met the inclusion criteria of the review, including how many reviewers screened each record and each report retrieved, whether they worked independently, and if applicable, details of automation tools used in the process.	Page 6, 7
Data collection process	9	Specify the methods used to collect data from reports, including how many reviewers collected data from each report, whether they worked independently, any processes for obtaining or confirming data from study investigators, and if applicable, details of automation tools used in the process.	Page 6, 7
Data items	10a	List and define all outcomes for which data were sought. Specify whether all results that were compatible with each outcome domain in each study were sought (e.g. for all measures, time points, analyses), and if not, the methods used to decide which results to collect.	Page 6, 7
	10b	List and define all other variables for which data were sought (e.g. participant and intervention characteristics, funding sources). Describe any assumptions made about any missing or unclear information.	Page 6, 7
Study risk of bias assessment	11	Specify the methods used to assess risk of bias in the included studies, including details of the tool(s) used, how many reviewers assessed each study and whether they worked independently, and if applicable, details of automation tools used in the process.	Page 7, 8
Effect measures	12	Specify for each outcome the effect measure(s) (e.g. risk ratio, mean difference) used in the synthesis or presentation of results.	Page 7, 8

Section and Topic	Item #	Checklist item	Location where item is reported
Synthesis methods	13a	Describe the processes used to decide which studies were eligible for each synthesis (e.g. tabulating the study intervention characteristics and comparing against the planned groups for each synthesis (item #5)).	Page 6, 7
	13b	Describe any methods required to prepare the data for presentation or synthesis, such as handling of missing summary statistics, or data conversions.	Page 6, 7
	13c	Describe any methods used to tabulate or visually display results of individual studies and syntheses.	Page 6, 7
	13d	Describe any methods used to synthesize results and provide a rationale for the choice(s). If meta-analysis was performed, describe the model(s), method(s) to identify the presence and extent of statistical heterogeneity, and software package(s) used.	Page 6, 7
	13e	Describe any methods used to explore possible causes of heterogeneity among study results (e.g. subgroup analysis, meta-regression).	Page 6, 7
	13f	Describe any sensitivity analyses conducted to assess robustness of the synthesized results.	Page 6, 7
Reporting bias assessment	14	Describe any methods used to assess risk of bias due to missing results in a synthesis (arising from reporting biases).	Page 6, 7
Certainty assessment	15	Describe any methods used to assess certainty (or confidence) in the body of evidence for an outcome.	Page 6, 7
**RESULTS**			
Study selection	16a	Describe the results of the search and selection process, from the number of records identified in the search to the number of studies included in the review, ideally using a flow diagram.	Page 7, 8
	16b	Cite studies that might appear to meet the inclusion criteria, but which were excluded, and explain why they were excluded.	Page 7, 8
Study characteristics	17	Cite each included study and present its characteristics.	Page 8, 9 Supplementary S3
Risk of bias in studies	18	Present assessments of risk of bias for each included study.	Supplementary S4
Results of individual studies	19	For all outcomes, present, for each study: (a) summary statistics for each group (where appropriate) and (b) an effect estimate and its precision (e.g. confidence/credible interval), ideally using structured tables or plots.	Page 8-12 Supplementary S5
Results of syntheses	20a	For each synthesis, briefly summarise the characteristics and risk of bias among contributing studies.	Page 8-13 Supplementary S5
	20b	Present results of all statistical syntheses conducted. If meta-analysis was done, present for each the summary estimate and its precision (e.g. confidence/credible	Page 8-12 Supplementary

Section and Topic	Item #	Checklist item	Location where item is reported
		interval) and measures of statistical heterogeneity. If comparing groups, describe the direction of the effect.	S5
	20c	Present results of all investigations of possible causes of heterogeneity among study results.	Page 8-12 Supplementary S5
	20d	Present results of all sensitivity analyses conducted to assess the robustness of the synthesized results.	Page 8-12 Supplementary S5
Reporting biases	21	Present assessments of risk of bias due to missing results (arising from reporting biases) for each synthesis assessed.	Page 12 Supplementary S6
Certainty of evidence	22	Present assessments of certainty (or confidence) in the body of evidence for each outcome assessed.	Supplementary S4
**DISCUSSION**			
Discussion	23a	Provide a general interpretation of the results in the context of other evidence.	Page 13-18
	23b	Discuss any limitations of the evidence included in the review.	Page 18, 19
	23c	Discuss any limitations of the review processes used.	Page 18, 19
	23d	Discuss implications of the results for practice, policy, and future research.	Page 19
**OTHER INFORMATION**			
Registration and protocol	24a	Provide registration information for the review, including register name and registration number, or state that the review was not registered.	Page 5
	24b	Indicate where the review protocol can be accessed, or state that a protocol was not prepared.	NS
	24c	Describe and explain any amendments to information provided at registration or in the protocol.	NS
Support	25	Describe sources of financial or non-financial support for the review, and the role of the funders or sponsors in the review.	Page 20
Competing interests	26	Declare any competing interests of review authors.	Page 20
Availability of data, code and other materials	27	Report which of the following are publicly available and where they can be found: template data collection forms; data extracted from included studies; data used for all analyses; analytic code; any other materials used in the review.	NS

From: Page MJ, McKenzie JE, Bossuyt PM, Boutron I, Hoffmann TC, Mulrow CD, et al The PRISMA 2020 statement: an updated guideline for reporting systematic reviews. BMJ 2021;372:n71. doi: 10.1136/bmj.n71. For more information, visit: http://www.prisma-statement.org/

Supplementary S2: The literature search strategy

PubMed:

Sarcopenia:

(((sarcopeni*[MeSH Terms]) OR (sarcopeni*[Title/Abstract] OR myopeni*[Title/Abstract]))) AND ((Biomarkers[Title/Abstract]) OR (Biomarkers[Mesh]) OR (Senescence markers[Title/Abstract]) OR (Inflamm*[Mesh]) OR (Inflamm*[Title/Abstract]) OR (Cytokines[Mesh]) OR (Cytokines[Title/Abstract]) OR (Myokin*[Mesh]) OR (Myokin*[Title/Abstract]) OR (Metabolic markers[Title/Abstract]) OR (Oxidative stress markers[Title/Abstract]) OR (Mitochondri* markers[Title/Abstract]) OR (Cerebrospinal fluid markers[Title/Abstract]) OR (Saliva* markers[Title/Abstract]) OR (Plasma markers[Title/Abstract]) OR (Urine markers[Title/Abstract]) OR (Biochemical markers[Title/Abstract]) OR (Surrogate markers[Title/Abstract]) OR (Serum markers[Title/Abstract]) OR (Immun* markers[Title/Abstract]) OR (Biologic* markers[Title/Abstract]) OR (Body composition markers[Title/Abstract])) NOT (((((((((animals[MeSH Terms]) OR (animal*)) OR (animals)) OR (animal)) OR (rat)) OR (mice)) OR (mouse)) OR (rats)) OR (rabbit))) 741

sarcopenic obesity:

((("Obesity"[Mesh]) OR (Obesity[Title/Abstract]) OR (Overweight[Mesh])OR (Overweight[Title/Abstract])) AND (("Sarcopeni*"[Mesh]) OR (Sarcopeni*[Title/Abstract])) OR (sarcopenic obesity [Title/Abstract])) AND (((Biomarkers[Title/Abstract]) OR (Biomarkers[Mesh]) OR (Outcomes[Title/Abstract]) OR (Senescence markers[Title/Abstract]) OR (Inflamm*[Mesh]) OR (Inflamm*[Title/Abstract]) OR (Cytokines[Mesh]) OR (Cytokines[Title/Abstract]) OR (Myokin*[Mesh]) OR (Myokin*[Title/Abstract]) OR (Metabolic markers[Title/Abstract]) OR (Oxidative stress markers[Title/Abstract]) OR (Mitochondri* markers[Title/Abstract]) OR (Cerebrospinal fluid markers[Title/Abstract]) OR (Saliva* markers[Title/Abstract]) OR (Plasma markers[Title/Abstract]) OR (Urine markers[Title/Abstract]) OR (Biochemical markers[Title/Abstract]) OR (Surrogate markers[Title/Abstract]) OR (Serum markers[Title/Abstract]) OR (Immun* markers[Title/Abstract]) OR (Biologic* markers[Title/Abstract]) OR (Body composition markers[Title/Abstract])) NOT (((((((((animals[MeSH Terms]) OR (animal*)) OR (animals)) OR (animal)) OR (rat)) OR (mice)) OR (mouse)) OR (rats)) OR (rabbit))))
249

Web of science:

Sarcopenia:

(TI= (sarcopeni* OR myopeni*) OR AB= (sarcopeni* OR myopeni*)) AND (TI=(Biomarkers OR Senescence markers OR Inflamm* OR Cytokines OR Myokin* OR Metabolic markers OR Oxidative stress markers OR Mitochondri* markers OR Cerebrospinal fluid markers OR Saliva* markers OR Plasma markers OR Urine markers OR Biochemical markers OR Surrogate markers OR Serum markers OR Immun* markers OR Biologic* markers OR Body composition markers） OR AB=(Biomarkers OR Senescence markers OR Inflamm* OR Cytokines OR Myokin* OR Metabolic markers OR Oxidative stress markers OR Mitochondri* markers OR Cerebrospinal fluid markers OR Saliva* markers OR Plasma markers OR Urine markers OR Biochemical markers OR Surrogate markers OR Serum markers OR Immun* markers OR Biologic* markers OR Body composition markers)) NOT (TI= (animal* OR rat OR mice OR mouse OR rats OR rabbit) OR AB= (animal* OR rat OR mice OR mouse OR rats OR rabbit))

4236

sarcopenic obesity:

(TI=(sarcopenic obesity OR ((Obesity OR Overweight) AND Sarcopeni*)) OR AB= (sarcopenic obesity OR ((Obesity OR Overweight) AND Sarcopeni*))) AND (TI=(Biomarkers OR Senescence markers OR Inflamm* OR Cytokines OR Myokin* OR Metabolic markers OR Oxidative stress markers OR Mitochondri* markers OR Cerebrospinal fluid markers OR Saliva* markers OR Plasma markers OR Urine markers OR Biochemical markers OR Surrogate markers OR Serum markers OR Immun* markers OR Biologic* markers OR Body composition markers） OR AB=(Biomarkers OR Senescence markers OR Inflamm* OR Cytokines OR Myokin* OR Metabolic markers OR Oxidative stress markers OR Mitochondri* markers OR Cerebrospinal fluid markers OR Saliva* markers OR Plasma markers OR Urine markers OR Biochemical markers OR Surrogate markers OR Serum markers OR Immun* markers OR Biologic* markers OR Body composition markers)) NOT (TI= (animal* OR rat OR mice OR mouse OR rats OR rabbit) OR AB= (animal* OR rat OR mice OR mouse OR rats OR rabbit))   899

Embase:

Sarcopenia:

#1 'sarcopenia'/exp OR 'sarcopenia':ti,ab,kw OR 'age-related muscle atrophy':ti,ab,kw OR 'age-related muscle decline':ti,ab,kw OR 'age-related muscular decline':ti,ab,kw OR 'age-related muscular degeneration':ti,ab,kw OR 'ageing-related muscle atrophy':ti,ab,kw OR 'geriatric muscular atrophy':ti,ab,kw   31819+2569

#2 'Biomarkers'/exp or 'Biomarkers':ab,ti,kw OR 'Senescence markers':ab,ti,kw OR 'Inflamm*'/exp or 'Inflamm*':ab,ti,kw OR 'Cytokines'/exp or 'Cytokines':ab,ti,kw OR ' Myokin*'/exp or ' Myokin*':ab,ti,kw OR

'Metabolic markers ':ab,ti,kw OR 'Oxidative stress markers':ab,ti,kw OR ' Mitochondri* markers':ab,ti,kw or 'Cerebrospinal fluid markers':ab,ti,kw OR 'Saliva* markers':ab,ti,kw OR 'Plasma markers':ab,ti,kw OR 'Urine markers':ab,ti,kw OR 'Biochemical markers':ab,ti,kw OR 'Surrogate markers':ab,ti,kw OR 'Serum markers':ab,ti,kw OR 'Immun* markers':ab,ti,kw OR 'Biologic* markers':ab,ti,kw OR 'Body composition markers':ab,ti,kw 4289368

#3 (#1 AND #2) AND [humans]/lim 6978

sarcopenic obesity:

#4 'sarcopenic obesity':ti,ab,kw 2379

#5 'Obesity'/exp OR 'adipose tissue hyperplasia':ti,ab,kw OR 'adipositas':ti,ab,kw OR 'adiposity':ti,ab,kw OR 'alimentary obesity':ti,ab,kw OR 'body weight, excess':ti,ab,kw OR 'corpulency':ti,ab,kw OR 'fat overload syndrome':ti,ab,kw OR 'nutritional obesity':ti,ab,kw OR 'obesitas':ti,ab,kw OR 'overweight':ti,ab,kw OR 'obesity':ti,ab,kw 855262

#6 ((#4 OR (#1 AND #5)) AND #2) AND [humans]/lim 2793

Cochrane Library :

sarcopenia

#1 MeSH descriptor: [Sarcopenia] explode all trees

#2 ((sarcopeni* OR myopeni*)):ti,ab,kw (Word variations have been searched) 2929

#3 #1 OR #2 2929

#4 MeSH descriptor: [Biomarkers] explode all trees

#5 MeSH descriptor: [Inflammation] explode all trees

#6 MeSH descriptor: [Cytokines] explode all trees

#7 MeSH descriptor: [Myokines] explode all trees

#8 (Biomarkers OR Senescence markers OR Inflamm* OR Cytokines OR Myokin* OR Metabolic markers OR Oxidative stress markers OR Mitochondri* markers OR Cerebrospinal fluid markers OR Saliva* markers OR Plasma markers OR Urine markers OR Biochemical markers OR Surrogate markers OR Serum markers OR Immun* markers OR Biologic* markers OR Body composition markers):ti,ab,kw (Word variations have been searched) 195831

#9 #4 OR #5 OR #6 OR #7 OR #8 220745

#10 (animal* OR animals OR animal OR rat OR mice OR mouse OR rats OR rabbit):ti,ab,kw (Word variations have been searched) 49501

#11 (#3 AND #9) NOT #10 7775

sarcopenic obesity:

#12 (sarcopenic obesity):ti,ab,kw 163

#13 MeSH descriptor: [Obesity] explode all trees

#14 MeSH descriptor: [Overweight] explode all trees

#15 (obesity or weight):ti,ab,kw 169894

#16 (#13 OR #14 OR #15) AND (#1 OR #2) 835

#17 ((#12 OR #16) AND #9) NOT #10 197

Supplementary S3: Characteristics of included studies

Author	year	Country	Study design	Simple	Size, n	Assessment	Male/female, n	Mean age (years)	Age(sd) (years)	biomarker
Ze Chen et al.	2023	Germany	Cross-sectional	Hospitalized	80	ASMI plus IHG	35/45	81.4	6.4	metabolic and inflammatory
Chi-Hua Yen et al.	2022	China	Cross-sectional	Community-dwelling	99	ASMI plus IHG or WS	26/63	73.3	8.0	hormonal and metabolic
Lin Ying et al.	2022	China	Cross-sectional	Hospitalized	80	ASMI plus IHG or WS	43/27	73.0	5.7	inflammatory
Ming Li Yee et al.	2020	Australia	Cross-sectional	Hospitalized	39	SMI plus IHG	0/39	77.8	10.3	hormonal
Aishanjiang Wumaer et al.	2022	China	Case-control	Community-dwelling	152	Muscle mass plus IHG or WS	65/87	70.5	4.2	inflammatory
Dara Aldisi et al.	2022	Saudi	Cross-sectional	Community-dwelling	76	ASMI plus IHG or WS	0/76	66.8	5.7	metabolic and inflammatory
Jinghan Gao et al.	2022	China	cross-sectional	Hospitalized	235	SMI plus IHG or WS	160/75	64.4	10.68	inflammatory
Nana He et al.	2022	China	Cross-sectional	Community-dwelling	186	ASMI plus IHG or WS	68/118	76.17	0.58	metabolic
Sunny Singhal et al.	2019	India	cross-sectional	Hospitalized	100	ASMI plus IHG or WS	69/31	72.5	6.4	metabolic
Irina Balazs et al.	2024	Austria	cross-sectional	Hospitalized	88	ASMI plus IHG or WS	58/30	61.045	10.3	metabolic, hematologic and inflammatory

Jing Feng Zou et al.	2024	China	cross-sectional	Hospitalized	1674	SMI	1110/564	61.97	9.82	metabolic and inflammatory
Busra Can et al.	2017	Turkey	cross-sectional	Hospitalized	72	ASMI plus IHG or WS	27/45	78.75	6.38	metabolic and inflammatory
Anna Picca et al.	2022	Italy	cross-sectional	Community-dwelling	49	ASMI plus IHG or WS	14/35	75.2	4.5	inflammatory
Carmen Sánchez-Castellano et al.	2020	Spain	cross-sectional	Hospitalized	150	SMI plus IHG or WS	32/118	87.6	4.9	oxidative stress and inflammatory
Yong He et al.	2022	China	cross-sectional	Community-dwelling	4099	ASMI plus IHG or WS	1471/2628	60.3	8.9	metabolic, hematologic, and hormonal
Yawara Eguchi et al.	2021	Japan	cross-sectional	Hospitalized	70	SMI plus IHG or WS	0/80	74.2	8.6	metabolic and hormonal
Akihiko Shibamoto et al.	2023	Japan	cross-sectional	Hospitalized	424	SMI plus IHG	162/262	67.6	11.4	metabolic, hormonal and inflammatory
Cemile Özsürekci et al.	2021	Turkey	cross-sectional	Hospitalized	94	SMI plus IHG or WS	24/70	75.0	6.6	metabolic, hormonal and inflammatory
Masahiro Kameda et al.	2021	Japan	cross-sectional	Hospitalized	19	SMI plus IHG or WS	7/12	84.2	6.9	metabolic and inflammatory
Jiang Shiqi et al.	2024	China	cross-sectional	Community-dwelling	377	ASMI plus IHG	164/213	71.68	5.9	metabolic and inflammatory
Jiaying Ge et al.	2022	China	cross-sectional	Hospitalized	404	ASMI plus IHG	146/258	66.0	10.0	metabolic, hormonal and inflammatory
Ze Chen et al.	2023	Germany	cross-sectional	Hospitalized	80	ASMI plus IHG	35/45	81.4	6.4	inflammatory
Wei Ju Lee et al.	2020	China (Taiwan)	cross-sectional	Hospitalized	1582	ASMI plus IHG or WS	749/833	64.0	9.2	metabolic, hormonal and inflammatory
Chi Jen Lo et al.	2023	China (Taiwan)	cross-sectional	Community-dwelling	289	ASMI plus IHG or WS	123/661	81.8	7.1	metabolic, hormonal and inflammatory
Ana Coto Montes et al.	2017	Spain	cross-sectional	Community-dwelling	200	SMI plus IHG or WS	84/116	76.7	1.0	oxidative stress, metabolic and inflammatory
Huang-Chun Liu et al.	2021	China (Taiwan)	cross-sectional	Community-dwelling	77	SMI plus IHG or WS	41/36	78.7	6.1	inflammatory
Riccardo Calvani et al.	2021	Italy	cross-sectional	Community-dwelling	200	Muscle mass plus IHG or WS	75/125	76.2	4.7	hormonal
Shengwu Yu et al.	2022	China	cross-sectional	Hospitalized	212	ASMI plus IHG	89/123	77.4	11.5	hormonal and inflammatory

Suguru Hirose et al.	2020	Japan	cross-sectional	Hospitalized	348	SMI plus IHG or WS	234/114	61.2	19.7	hormonal
Jun-Il Yoo et al.	2021	Korea	cross-sectional	Hospitalized	83	ASMI plus IHG or WS	25/58	72.1	11.1	hormonal
Hyung Eun Shin et al.	2022	Korea	cross-sectional	Hospitalized	20	ASMI plus IHG or WS	20/0	82.0	2.8	metabolic
Emanuele Marzetti et al.	2014	Italy	cross-sectional	Hospitalized	42	MSI plus IHG	10/32	83.7	8.6	hematologic, metabolic and hormonal
Ma Sihui et al.	2023	China	case-control	Hospitalized	142	SMI plus IHG	73/69	73.0	11.3	hematologic, metabolic and hormonal
Akihiko Shibamoto et al.	2023	Japan	cross-sectional	Hospitalized	451	SMI plus IHG	288/163	69.1	10.8	hematologic, metabolic and hormonal
Rui Xu et al.	2022	China	cross-sectional	Hospitalized	80	ASMI plus IHG or WS	45/35	76.3	1.8	metabolic
Andrea Dalbeni et al.	2023	Italy	cross-sectional	Hospitalized	92	SMI plus IHG	38/54	70.4	13.9	metabolic, hematologic and inflammatory
Tianjiao Tang et al.	2020	China	cross-sectional	Community-dwelling	384	ASMI plus IHG or WS	160/224	71.5	5.8	metabolic, hematologic and inflammatory
Hyung Eun Shin et al.	2023	Korea	cohort study	Community-dwelling	1021	ASMI plus IHG or WS	498/523	75.7	3.8	metabolic, hematologic and inflammatory
Mengting Yin et al.	2022	China	cross-sectional	Community-dwelling	2837	ASMI plus IHG or WS	0/2837	61.6	8.1	metabolic
Takahiro Yajima et al.	2023	Japan	cross-sectional	Hospitalized	85	ASMI plus IHG or WS	58/27	67.8	13.1	metabolic
YuanYuei Chen et al.	2021	China	cross-sectional	Hospitalized	408	SMI plus IHG or WS	173/235	74.3	35.7	metabolic
Zeynel Abidin Öztürk et al.	2018	Turkey	cross-sectional	Hospitalized	419	SMI plus IHG or WS	179/240	71.8	6.0	hematologic and inflammatory
Jaw-Shiun Tsai et al.	2022	China	cross-sectional	Hospitalized	48	ASMI plus IHG or WS	20/28	79.2	6.0	hematologic and inflammatory
Zhen Fan et al.	2022	China	cross-sectional	Hospitalized	97	ASMI plus IHG or WS	50/47	72.4	6.3	inflammatory
Hanako Nakajima et al.	2023	Japan	cross-sectional	Community-dwelling	99	ASMI plus IHG	56/43	63.7	11.9	metabolic
Akihiko Takagi et al.	2022	Japan	cross-sectional	Hospitalized	114	ASMI	74/40	68.4	10.5	metabolic
Misa Nakamura et al.	2024	Japan	cross-sectional	Community-dwelling	269	ASMI plus IHG or WS	63/206	74.5	6.5	metabolic
Chen-Lu Chang et al.	2023	China	cross-sectional	Community-dwelling	816	ASMI plus IHG or WS	450/366	74.9	7.0	metabolic

Jin-Liang Chen et al.	2021	China	cross-sectional	Hospitalized	437	ASMI plus IHG or WS	197/240	71.7	7.9	hematologic and metabolic
Chuan Wang et al.	2021	China	cross-sectional	Hospitalized	251	ASMI	124/127	61.7	6.6	metabolic
Fan Han et al.	2021	China	case-control	Hospitalized	162	ASMI plus IHG or WS	162/0	73.4	7.8	metabolic, hormonal and inflammatory
Hye-Sun Park et al.	2019	Korea	cross-sectional	Hospitalized	153	Muscle mass plus IHG	0/153	72.2	6.0	hormonal
Ahmet Yalcin et al.	2018	Turkey	cross-sectional	Hospitalized	160	SMI plus IHG or WS	80/80	79.3	21.2	hormonal and inflammatory
Leonardo Augusto Costa Teixeira et al.	2024	Brazil	cross-sectional	Community-dwelling	71	ASMI plus IHG or WS	0/71	74.5	7.0	hormonal and inflammatory
So Jeong Park et al.	2024	Korea	cross-sectional	Community-dwelling	134	ASMI plus IHG or WS	121/13	76.4	5.6	hormonal
Jae Young Jang et al.	2023	Korea	cross-sectional	Community-dwelling	945	ASMI plus IHG or WS	453/492	75.6	3.9	hormonal
Lin Li et al.	2023	China	cross-sectional	Hospitalized	288	ASMI plus IHG or WS	288/0	66.7	5.9	hormonal and inflammatory
Wei-Ju Lin et al.	2023	China (Taiwan)	cross-sectional	Community-dwelling	484	ASMI plus IHG or WS	241/243	69.3	8.4	inflammatory
Francesco Landi et al.	2021	Germany	cross-sectional	Community-dwelling	332	MAMC plus IHG or WS	104/228	85.8	4.8	hormonal and inflammatory
Francesco Cacciatore et al.	2015	Italy	cross-sectional	Hospitalized	337	MSI plus IHG or WS	143/194	76.2	6.7	hormonal and inflammatory
Yanxia Lu et al.	2020	Singapore	Cross-sectional	Community-dwelling	189	SMI plus IHG or WS	70/119	73.2	5.3	hormonal and metabolic
Mohamad Khalil et al.	2024	Italy	cross-sectional	Hospitalized	79	ASMI plus IHG or WS	46/33	78.2	0.9	metabolic and inflammatory
Dahan da Cunha Nascimento et al.	2018	Brazil	cross-sectional	Community-dwelling	64	Muscle mass plus IHG	0/64	68.4	6.0	metabolic, hematologic and inflammatory
Julio C. Ribeiro et al.	2021	Brazil	cross-sectional	Community-dwelling	247	ASMI plus IHG	112/135	68.0	6.7	metabolic, hormonal and inflammatory
Yoo Mee Kim et al.	2019	Korea	cross-sectional	Community-dwelling	3267	Muscle mass	1080/2187	64.1	9.0	metabolic and hormonal
Maurílio Tiradentes Dutra et al.	2017	Brazil	cross-sectional	Community-dwelling	130	Muscle mass plus IHG	0/130	66.7	5.2	inflammatory
Chuan Wei Yang et al.	2015	China	cross-sectional	Community-dwelling	844	ASMI	448/396	73.9	6.2	metabolic and inflammatory

Amy E. Mendham et al.	2021	South Africa	cross-sectional	Community-dwelling	122	ASMI plus IHG or WS	0/122	67.3	5.3	metabolic, hormonal and inflammatory
Simone Perna et al.	2017	Italy	cross-sectional	Hospitalized	639	SMI plus IHG	196/443	81.4	1.1	hematologic, metabolic, hormonal and inflammatory
Sheetal Sarangi et al.	2024	India	cross-sectional	Hospitalized	90	ASMI plus IHG or WS	0/90	60.8	5.4	metabolic
Francesco Fantin et al.	2024	Italy	cross-sectional	Hospitalized	77	SMI plus IHG	34/43	79.0	9.7	metabolic and inflammatory
Yoshihiro Yoshimura et al.	2023	Japan	cross-sectional	Community-dwelling	760	ASMI plus IHG or WS	408/352	71.0	9.0	metabolic
Nima Montazeri-Najababady et al.	2021	Iranian	cross-sectional	Community-dwelling	206	ASMI plus IHG or WS	NS	70.5	4.8	metabolic
David Scotta et al.	2018	Australia	cross-sectional	Community-dwelling	1231	ASMI plus IHG	1231/0	76.7	5.0	hematologic, metabolic, hormonal and inflammatory
Simone Perna et al.	2018	Italy	cross-sectional	Hospitalized	141	SMI plus IHG	141/0	77.2	9.0	metabolic and inflammatory
Enivaldo Pereira dos Santos et al.	2014	Brazil	cross-sectional	Community-dwelling	149	ASMI	0/149	67.2	6.1	metabolic
Chia Wen Lu et al.	2013	China (Taiwan)	cross-sectional	Community-dwelling	600	SMI	144/456	63.6	10.1	hematologic, metabolic, hormonal and inflammatory
X LIU et al.	2020	China	cross-sectional	Community-dwelling	4500	ASMI plus IHG or WS	1627/2873	62.4	8.3	metabolic and inflammatory
Shangjin Lin et al.	2024	China	cross-sectional	Hospitalized	207	ASMI plus WS	98/109	64.4	13.7	inflammatory
Misa Nakamura et al.	2024	Japan	cross-sectional	Hospitalized	150	SMI plus IHG or WS	37/113	74.3	6.3	hematologic
Bahar Bektan Kanat et al.	2024	Turkey	cross-sectional	Hospitalized	262	ASMI plus WS	66/196	76.6	7.3	inflammatory and hematologic

Abbreviations: ASMI, appendicular skeletal muscle index; IHG, isometric handgrip; WS, walking speed

Supplementary S4: SO diagnostic criteria.

Author	year	Sarcopenia Assessment	Obesity Assessment	Instruments
Mohamad Khalil et al.	2024	EWGSOP2	FM>25% (M) / >35% (F)	BIA

Dahan da Cunha	2018	ALM/BMI<0.512	FM >38% (F)	DXA
Julio C. Ribeiro et al.	2021	EWGSOP2	BMI ⩾ 30	DXA
Yoo Mee Kim et al.	2019	ASM/Wt > 1	FM>25% (M) / >35% (F)	DXA
Maurílio Tiradentes Dutra et al.	2017	Muscle mass plus IHG	AFFM − AFFM pred ≤3.4	DXA
Chuan Wei Yang et al.	2015	SMI<6.87 (M)/<5.64 (F)	FM>27.82% (M) / >37.61% (F)	DXA
Amy E. Mendham et al.	2021	FNIH sarcopenia project	BMI > 30.0	DXA
Simone Perna et al.	2017	SMI plus IHG	BMI > 25.0	DXA
Sheetal Sarangi et al.	2024	EWGSOP2	BMI > 25.0	DXA
Francesco Fantin et al.	2024	/	ESPEN and EASO Consensus Statement	BIA
Nima Montazeri-Najababady et al.	2021	AWGS	FM/FFM>0.80	BIA
David Scotta et al.	2018	EWGSOP	FM >30%	DXA
Simone Perna et al.	2018	SMI<6.87 (M)/<5.64 (F)	BMI > 25	DXA
Enivaldo Pereira dos Santos et al.	2014	AFFM≤5.45	AFFM − AFFM pred ≤3.4	DXA
Chia Wen Lu et al.	2013	SMM/Wt≦37% (M)/≦27.6% (F)	BMI > 25	BIA
X LIU et al.	2020	AWGS	FM>34.30% (M) / >42.10% (F)	BIA

EWGSOP2: European Working Group on Sarcopenia in Older People 2. FM: Fat Mass. M: Male. F: Female. BIA: Bioimpedance analysis. ALM: Appendicular Lean Mass. DXA: Dual-energy X-ray absorptiometry. ASM: Appendicular skeletal muscle mass. Wt: body weight. SMI: Skeletal muscle index. AFFM: Appendicular Fat-Free Mass. AFFM pred=−14.529+(17.989×height)+(0.1307×FM). FNIH: The Foundation for the National Institutes of Health. ESPEN: The European Society for Clinical Nutrition and Metabolism. EASO: The European Association for the Study of Obesity. AWGS: Asian Working Group on Sarcopenia. FFM: Fat-Free Mass. EWGSOP: The European Working Group on Sarcopenia.

Supplementary S5: Results of qualitative analysis.

a) Cross-sectional and cohort studies

Sarcopenia

Year	Authors	1	2	3	4	5	6	7	8	9	10	11	12	13	14	Overall score	qualitative
2023	Ze Chen et al.	Y	Y	NR	Y	N	NA	NA	NA	Y	NA	Y	NR	NR	Y	7	good
2022	Chi Hua Yen et al.	Y	Y	NR	Y	N	NA	NA	NA	Y	NA	Y	NR	NR	Y	6	fair
2022	Lin Ying et al.	Y	Y	NR	Y	Y	NA	NA	NA	Y	NA	Y	NR	NR	N	6	fair
2020	Ming Li Yee et al.	Y	Y	NR	Y	Y	NA	NA	NA	Y	NA	Y	NR	NR	N	6	fair
2022	Aishanjiang Wumaer et al.	Y	Y	Y	Y	N	NA	NA	NA	Y	NA	Y	NR	NR	Y	7	good
2022	Dara Aldisi et al.	Y	Y	NR	Y	Y	NA	NA	NA	Y	NA	Y	NR	NR	N	6	fair
2022	Jinghan Gao et al.	Y	Y	NR	Y	Y	NA	NA	NA	Y	NA	Y	NR	NR	N	6	fair
2022	Nana He et al.	Y	Y	NR	Y	Y	NA	NA	NA	Y	NA	Y	NR	NR	Y	7	good
2022	Sunny Singhal et al.	Y	Y	NR	Y	N	NA	NA	NA	Y	NA	Y	NR	NR	Y	6	fair
2024	Irina Balazs et al.	Y	Y	NR	Y	N	NA	NA	NA	Y	NA	Y	NR	NR	Y	6	fair
2024	Jing-Feng Zou et al.	Y	Y	NR	Y	N	NA	NA	NA	Y	NA	Y	NR	NR	Y	6	fair
2017	Busra Can et al.	Y	Y	NR	Y	N	NA	NA	NA	Y	NA	Y	NR	NR	Y	6	fair
2022	Anna Picca et al.	Y	Y	N	Y	N	NA	NA	NA	Y	NA	Y	NR	NR	Y	6	fair
2020	Carmen Sánchez-Castellano et al.	Y	Y	N	Y	N	NA	NA	NA	Y	NA	Y	NR	NR	N	5	fair
2022	Yong He et al.	Y	Y	Y	Y	N	NA	NA	NA	Y	NA	Y	NR	NR	N	6	fair
2021	Yawara Eguchi et al.	Y	Y	NR	Y	N	NA	NA	NA	Y	NA	Y	NR	NR	N	5	fair
2023	Akihiko Shibamoto et al.	Y	Y	N	Y	N	NA	NA	NA	Y	NA	Y	NR	NR	Y	6	fair
2021	Cemile Özsürekci et al.	Y	Y	NR	Y	N	NA	NA	NA	Y	NA	Y	NR	NR	N	5	fair
2021	Masahiro Kameda et al.	Y	Y	NR	Y	NR	NA	NA	NA	Y	NA	Y	NR	NR	NR	5	fair
2024	Jiang Shiqi et al.	Y	Y	NR	Y	Y	NA	NA	NA	Y	NA	Y	NR	NR	N	6	fair
2022	Jiaying Ge et al.	Y	Y	NR	Y	NR	NA	NA	NA	Y	NA	Y	NR	Y	Y	7	good
2023	Ze Chen et al.	Y	Y	Y	Y	NR	NA	NA	NA	Y	NA	Y	Y	Y	Y	8	good
2020	Wei-Ju Lee et al.	Y	Y	NR	Y	NR	NA	NA	NA	Y	NA	Y	Y	Y	Y	7	good
2023	Chi-Jen Lo et al.	Y	Y	Y	Y	NR	NA	NA	NA	Y	NA	Y	NR	NR	Y	7	good
2017	Ana Coto Montes et al.	Y	Y	NR	Y	NR	NA	NA	NA	Y	NA	Y	NR	NR	Y	6	fair
2021	Huang Chun Liu et al.	Y	Y	NR	Y	NR	NA	NA	NA	Y	NA	Y	NR	NR	N	5	fair
2021	Riccardo Calvani et al.	Y	Y	NR	Y	NR	NA	NA	NA	Y	NA	Y	NR	NR	Y	6	fair
2022	Shengwu Yu et al.	Y	Y	NR	N	Y	NA	NA	NA	Y	NA	Y	NR	NR	Y	6	fair
2020	Suguru Hirose et al.	Y	Y	NR	Y	NR	NA	NA	NA	Y	NA	Y	NR	NR	Y	6	fair
2021	Jun-Il Yoo et al.	Y	Y	NR	Y	Y	NA	NA	NA	Y	NA	Y	NR	NR	Y	6	fair
2022	Hyung Eun Shin et al.	Y	Y	NR	Y	Y	NA	NA	NA	Y	NA	Y	NR	NR	Y	7	good
2014	Emanuele Marzetti et al.	Y	Y	NR	Y	NR	NA	NA	NA	Y	NA	Y	NR	NR	Y	6	fair
2023	Akihiko Shibamoto et al.	Y	Y	NR	Y	NR	NA	NA	NA	N	NA	Y	NR	NR	Y	5	fair
2022	Rui Xu et al.	Y	Y	NR	Y	NR	NA	NA	NA	Y	NA	Y	NR	NR	Y	6	fair
2023	Andrea Dalbeni et al.	Y	Y	NR	Y	NR	NA	NA	NA	Y	NA	Y	NR	NR	Y	6	fair
2020	Tianjiao Tang et al.	Y	Y	NR	Y	NR	NA	NA	NA	Y	NA	Y	Y	Y	Y	7	good
2023	Hyung Eun Shin et al.	Y	Y	NR	Y	NR	NA	NA	NA	Y	N	Y	NR	Y	Y	8	good
2022	Mengting Yin et al.	Y	Y	Y	Y	NR	NA	NA	NA	Y	NA	Y	NR	NR	Y	7	good
2023	Takahiro Yajima et al.	Y	Y	NR	Y	NR	NA	NA	NA	Y	NA	Y	NR	NR	Y	6	fair
2021	YuanYuei Chen et al.	Y	Y	NR	Y	NR	NA	NA	NA	Y	NA	Y	NR	NR	Y	6	fair
2018	Zeynel Abidin Öztürk et al.	Y	Y	NR	Y	NR	NA	NA	NA	Y	NA	Y	NR	NR	Y	6	fair
2022	Jaw-Shiun Tsai et al.	Y	Y	NR	Y	Y	NA	NA	NA	Y	NA	Y	Y	Y	Y	8	good

2022	Zhen Fan et al.	Y	Y	NR	Y	Y	NA	NA	NA	Y	NA	Y	NR	NR	Y	7	good
2023	Hanako Nakajima et al.	Y	Y	NR	Y	NR	NA	NA	NA	N	NA	Y	NR	NR	Y	5	fair
2022	Akihiko Takagi et al.	Y	Y	N	Y	N	NA	NA	NA	Y	NA	Y	NR	NR	Y	6	fair
2023	Barbara Morawin et al.	Y	Y	NR	Y	NR	NA	NA	NA	Y	NA	Y	NR	NR	N	5	fair
2024	Misa Nakamura et al.	Y	Y	NR	Y	Y	NA	NA	NA	Y	NA	Y	NR	NR	Y	7	good
2023	Chen Lu Chang et al.	Y	Y	NR	Y	NR	NA	NA	NA	Y	NA	Y	NR	NR	Y	6	fair
2021	Jin Liang Chen et al.	Y	Y	NR	Y	NR	NA	NA	NA	Y	NA	Y	NR	NR	Y	6	fair
2021	Chuan Wang et al.	Y	Y	NR	Y	NR	NA	NA	NA	Y	NA	Y	NR	NR	Y	6	fair
2019	Hye-Sun Park et al.	Y	Y	NR	Y	NR	NA	NA	NA	Y	NA	Y	NR	NR	Y	6	fair
2018	Ahmet Yalcin et al.	Y	Y	N	Y	N	NA	NA	NA	Y	NA	Y	NR	NR	Y	6	fair
2024	Leonardo Augusto Costa Teixeira et al.	Y	Y	NR	Y	Y	NA	NA	NA	Y	NA	Y	NR	NR	N	6	fair
2024	So Jeong Park et al.	Y	Y	NR	Y	NR	NA	NA	NA	Y	NA	Y	NR	NR	Y	6	fair
2023	Jae Young Jang et al.	Y	Y	NR	Y	NR	NA	NA	NA	N	N	Y	NR	NR	Y	5	fair
2023	Lin Li et al.	Y	Y	NR	Y	NR	NA	NA	NA	Y	NA	Y	NR	NR	Y	6	fair
2023	Wei-Ju Lin et al.	Y	Y	NR	Y	NR	NA	NA	NA	N	N	Y	NR	NR	Y	5	fair
2021	Francesco Landi et al.	Y	Y	NR	Y	NR	NA	NA	NA	N	N	Y	NR	NR	Y	5	fair
2015	Francesco Cacciatore et al.	Y	Y	NR	Y	NR	NA	NA	NA	Y	NA	Y	NR	NR	Y	6	fair
2020	Yanxia Lu et al.	Y	Y	N	Y	N	NA	NA	NA	Y	NA	Y	NR	NR	Y	6	fair
2023	Shi-Teng Lee et al.	Y	Y	NR	Y	NR	NA	NA	NA	Y	NA	Y	NR	NR	N	5	fair
**Sarcopenic Obesity**																	
2024	Mohamad Khalil et al.	Y	Y	NR	Y	N	NA	NA	NA	Y	NA	Y	Y	Y	Y	7	good
2018	Dahan da Cunha Nascimento et al.	Y	Y	NR	Y	Y	NA	NA	NA	Y	NA	Y	NR	NR	Y	7	good
2021	Julio C. Ribeiro et al.	Y	Y	NR	Y	NR	NA	NA	NA	Y	NA	Y	NR	NR	Y	6	fair
2019	Yoo Mee Kim et al.	Y	Y	NR	Y	NR	NA	NA	NA	Y	NA	Y	NR	NR	Y	6	fair
2017	Maurílio Tiradentes Dutra et al.	Y	Y	NR	Y	NR	NA	NA	NA	Y	NA	Y	NR	NR	Y	6	fair
2015	Chuan-Wei Yang et al.	Y	Y	NR	Y	N	NA	NA	NA	Y	NA	Y	NR	NR	Y	6	fair
2021	Amy E. Mendham et al.	Y	Y	NR	Y	Y	NA	NA	NA	Y	NA	Y	NR	NR	Y	7	good
2017	Simone Perna et al.	Y	Y	NR	Y	NR	NA	NA	NA	Y	NA	Y	NR	NR	Y	6	fair
2024	Sheetal Sarangi et al.	Y	Y	NR	Y	Y	NA	NA	NA	Y	NA	Y	NR	NR	N	6	fair
2024	Francesco Fantin et al.	Y	Y	NR	Y	N	NA	NA	NA	Y	NA	Y	NR	NR	Y	6	fair
2021	Nima Montazeri-Najababady et al.	Y	Y	NR	Y	N	NA	NA	NA	Y	NA	Y	NR	NR	Y	6	fair
2018	David Scotta et al.	Y	Y	Y	Y	NR	NA	NA	NA	Y	NA	Y	NR	NR	Y	7	good
2018	Simone Perna et al.	Y	Y	NR	Y	N	NA	NA	NA	Y	NA	Y	NR	NR	N	5	fair
2014	Enivaldo Pereira dos Santos et al.	Y	Y	NR	Y	NR	NA	NA	NA	Y	NA	Y	NR	NR	Y	6	fair
2013	Chia-Wen Lu et al.	Y	Y	NR	Y	NR	NA	NA	NA	Y	NA	Y	NR	NR	Y	6	fair
2020	X. LIU et al.	Y	Y	NR	Y	NR	NA	NA	NA	Y	NA	Y	NR	NR	Y	6	fair
~~2016~~	~~Simone Perna et al.~~	~~Y~~	~~Y~~	~~NR~~	~~Y~~	~~NR~~	~~NA~~	~~NA~~	~~NA~~	~~Y~~	~~NA~~	~~Y~~	~~NR~~	~~NR~~	~~Y~~	~~6~~	~~fair~~
2024	Shangjin Lin et al.	Y	Y	NR	Y	NR	Y	NA	NA	Y	NA	Y	NR	NR	N	6	fair
2024	Misa Nakamura et al.	Y	Y	NR	Y	NR	Y	NA	NA	Y	NA	Y	NR	NR	N	6	fair
2024	Bahar Bektan Kanat et al.	Y	Y	NR	Y	NR	Y	NA	NA	Y	NA	Y	NR	NR	N	6	fair

b) Case-control studies

Sarcopenia

Year	Authors	1	2	3	4	5	6	7	8	9	10	11	12	Overall score	qualitative
2023	Ma Sihui et al.	Y	Y	N	Y	Y	Y	NA	Y	NA	Y	NR	N	7	good
2021	Fan Han et al.	Y	Y	N	Y	Y	Y	NA	Y	NA	Y	NR	N	7	good

Supplementary S6: Meta-analysis stratified by age, setting, DC/LDC and Gender

		Sarcopenia (n=66)			Sarcopenic Obesity (n =16)		
		SMD	95% CI	I^2	SMD	95% CI	I^2
**Metabolic markers**							
**Glucose (9/11)**	Overall	-0.104	-0.25, 0.04	87.1%	0.00	-0.19, 0.0.19	77.1%
Age	<75	-0.01	-0.13, 0.10	75.8%	0.20	0.06, 0.34	49.3%
	>75	-0.29	-0.83, 0.25	93.3%	-0.58	-1.09, -0.06	75.9%
Setting	Hospitalized	-0.10	-0.24, 0.04	31.1%	-0.80	-1.46, -0.13	72.0%
	Community-dwelling	-0.11	-0.31, 0.09	91.7	0.18	0.05, 0.31	46.8%
DC/LDC	DC	-0.40	-0.99, 0.19	93.2%	-0.39	-0.84, 0.05	86.5%
	LDC	0.01	-0.10, 0.12	73.8%	0.25	0.07, 0.42	75.5%
Gender (male/female)	<1	-0.11	-0.30, 0.07	90.8%	0.10	-0.07, 0.28	71.3%
	>1	-0.10	-0.25, 0.04	23.3%	-0.84	-2.43, 0.74	87.7%

		SMD	95% CI	I^2	SMD	95% CI	I^2
**Cholesterol (33/12)**	Overall	-0.13	-0.32, 0.07	95.8%	-0.07	-0.37, 0.22	93.0%
Age	<75	-0.03	-0.14, 0.08	85.1%	0.19	0.03, 0.36	73.4%
	>75	-0.25	-1.61, 1.10	98.6%	-1.06	-2.98, 0.87	97.2%
Setting	Hospitalized	-0.23	-0.73, 0.26	97.1%	-0.52	-2.25, 1.21	97.8%
	Community-dwelling	0.01	-0.16, 0.18	92.2%	0.12	0.01, 0.23	36.5%
DC/LDC	DC	-0.68	-1.81, 0.46	98.1%	-0.77	-2.02, 0.47	97.5%
	LDC	0.04	-0.11, 0.18	91.8%	0.23	0.02, 0.43	69.4%
Gender (male/female)	<1	-0.24	-0.46, -0.01	96.3%	-0.09	-0.41, 0.24	93.6%
	>1	0.23	-0.29, 0.74	94.2%	0.03	-0.25, 0.30	-
**Triglycerides (30/14)**	Overall	-0.27	-0.45, -0.10	94.0%	0.27	0.08, 0.46	85.8%
Age	<75	-0.17	-0.26, -0.08	73.6%	0.24	0.03, 0.45	84.5%
	>75	-0.39	-1.10, 0.32	97%	0.33	-0.17, 0.83	87.1%
Setting	Hospitalized	-0.18	-0.77, 0.41	96.7%	0.74	0.05, 1.42	89.4%
	Community-dwelling	-0.30	-0.42, -0.19	83.2%	0.11	0.01, 0.22	39.7%
DC/LDC	DC	-0.62	-1.43, 0.19	97.0%	0.29	-0.05, 0.62	88.3%
	LDC	-0.13	-0.25, -0.02	84.0%	0.27	-0.03, 0.57	85.9%
Gender (male/female)	<1	-0.41	-0.61, -0.20	94.7%	0.30	0.07, 0.53	87.9%
	>1	0.06	-0.30, 0.43	92.7%	0.12	-0.06, 0.29	0.0%
**HDL (26/12)**	Overall	0.15	-0.03, 0.34	94.6%	-0.16	-0.41, 0.09	90.9%
Age	<75	0.18	-0.01, 0.37	94.6%	-0.20	-0.50, 0.09	92.4%
	>75	0.04	-0.59, 0.67	95.2%	0.10	-0.12, 0.33	0.0%

		SMD	95% CI	I^2	SMD	95% CI	I^2
Setting	Hospitalized	-0.29	-0.61, 0.04	83.5%	-0.84	-2.47, 0.78	93.3%
	Community-dwelling	0.34	0.12, 0.55	94.6%	-0.05	-0.28, 0.18	88.7%
DC/LDC	DC	0.02	-0.78, 0.82	95.7%	-0.01	-0.07, 0.06	0.0%
	LDC	0.17	-0.01, 0.36	94.4%	-0.22	-0.61, 0.17	91.8%
Gender (male/female)	<1	0.31	0.09, 0.53	95.2%	-0.13	-0.42, 0.16	90.9%
	>1	-0.24	-0.59, 0.11	91.7%	-0.29	-1.10, 0.51	94.9%
**LDL (25/9)**	Overall	0.14	-0.02, 0.30	91.5%	0.10	-0.03, 0.22	41.4%
Age	<75	0.06	-0.07, 0.19	87.0%	0.11	-0.02, 0.24	44.2%
	>75	0.54	-0.22, 1.31	95.5%	-0.31	-1.00, 0.38	-
Setting	Hospitalized	0.48	-0.13, 1.10	95.8%	0.13	-0.63, 0.89	72.1%
	Community-dwelling	0.03	-0.08, 0.13	76.6%	0.08	-0.03, 0.19	31.8%
DC/LDC	DC	0.30%	-0.17, 0.78	78.8%	0.02	-0.05, 0.08	0.0%
	LDC	0.08	-0.08, 0.25	92.3%	0.16	0.01, 0.30	20.0%
Gender (male/female)	<1	-0.03	-0.15, 0.09	78.6%	0.10	-0.05, 0.24	48.3%
	>1	0.76	0.16, 1.35	96.2%	0.10	-0.17, 0.37	-
**Creatinine (26/6)**	Overall	0.04	-0.07, 0.15	77.7%	0.25	-0.60, 0.11	96.6%
Age	<75	-0.04	-0.12, 0.05	60.8%	-0.23	-0.94, 0.48	89.4%
	>75	0.28	-0.04, 0.59	75.6%	0.51	-0.68, 1.70	95.1%
Setting	Hospitalized	0.05	-0.13, 0.23	80.5%	0.51	-0.68, 1.70	95.1%
	Community-dwelling	-0.02	-0.10, 0.05	35.7%	-0.23	-0.94, 0.48	89.4%
DC/LDC	DC	0.02	-0.31, 0.35	86.2%	0.51	-0.68, 1.70	95.1%
	LDC	0.02	-0.06, 0.11	56.0%	-0.23	-0.94, 0.48	89.4%

		SMD	95% CI	I^2	SMD	95% CI	I^2
Gender (male/female)	<1	0.10	-0.02, 0.23	79.8%	0.39	-0.55, 0.39	97.2%
	>1	-0.15	-0.38, 0.09	73.2%	-0.50	-1.39, 0.39	-
**Uric acid (13/4)**	Overall	-0.25	-0.43, -0.07	89.4%	1.14	-0.35, 2.64	98.8%
Age	<75	-0.41	-0.64, -0.19	92.8%	0.33	0.16, 0.51	0.0%
	>75	0.00	-0.31, 0.31	74.6%	1.90	-0.95, 4.75	98.9%
Setting	Hospitalized	-0.33	-0.68, 0.03	91.6%	1.90	-0.95, 4.75	98.9%
	Community-dwelling	-0.13	-0.26, 0.00	71.8%	0.33	0.16, 0.51	0.0%
DC/LDC	DC	-0.06	-0.36, 0.23	65.5%	1.90	-0.95, 4.75	98.9%
	LDC	-0.31	-0.53, -0.10	91.5%	0.33	0.16, 0.51	0.0%
Gender (male/female)	<1	-0.11	-0.23, 0.01	68.3%	-	-	-
	>1	-1.09	-1.67, -0.51	84.4%	-	-	-
**ALT (18/3)**	Overall	-0.22	-0.35, -0.09	85.0%	-0.02	-0.15, 0.10	38.8%
Age	<75	-0.24	-0.39, 0.09	89.7%	-	-	-
	>75	-0.10	-0.31, 0.10	0.0%	-	-	-
Setting	Hospitalized	-0.10	-0.22, 0.02	20.2%	-	-	-
	Community-dwelling	-0.33	-0.50, -0.15	91.8%	-	-	-
DC/LDC	DC	-0.00	-0.21, 0.20	14.7%	-	-	-
	LDC	-0.27	-0.42, -0.13	97.9%	-	-	-
Gender (male/female)	<1	-0.19	-0.29, -0.09	68.0%	-	-	-
	>1	-0.32	-0.96, 0.32	91.0%	-	-	-
**AST (17/3)**	Overall	0.02	-0.04, 0.09	36.9%	0.05	-0.02, 0.12	3.0%

		SMD	95% CI	I^2	SMD	95% CI	I^2
Age	<75	0.02	-0.05, 0.09	49.6%	-	-	-
	>75	0.03	-0.19, 0.25	9.4%	-	-	-
Setting	Hospitalized	0.06	-0.08, 0.20	23.9%	-	-	-
	Community-dwelling	0.01	-0.07, 0.09	54.6%	-	-	-
DC/LDC	DC	0.07	-0.14, 0.28	17.9%	-	-	-
	LDC	0.02	-0.05, 0.09	47.5%	-	-	-
Gender (male/female)	<1	0.04	-0.01, 0.10	16.5%	-	-	-
	>1	-0.02	-0.32, 0.28	59.1%	-	-	-
**Inflammatory markers**							
**IL-6 (17/5)**	Overall	0.25	-0.01, 0.52	88.7%	0.07	-0.24, 0.38	56.9%
Age	<75	0.37	-0.01, 0.75	92.0%	0.14	-0.14, 0.42	51.3%
	>75	0.06	-0.24, 0.37	70.4%	-0.77	-1.71, 0.17	-
Setting	Hospitalized	0.39	-0.07, 0.85	92.7%	-0.77	-1.71, 0.17	-
	Community-dwelling	0.09	-0.08, 0.25	38.3%	0.14	-0.14, 0.42	51.3%
DC/LDC	DC	0.03	-0.03, 0.34	65.9%	-0.77	-1.71, 0.17	-
	LDC	0.38	0.01, 0.75	92.1%	0.14	-0.14, 0.42	51.3%
Gender (male/female)	<1	0.13	-0.01, 0.27	24.7%	0.24	-0.13, 0.61	50.9%
	>1	0.47	-0.14, 1.08	95.2%	-0.26	-0.91, 0.38	51.7%
**TNF-α (14/3)**	Overall	0.40	0.09, 0.71	89.7%	-0.10	-0.30, 0.10	0.0%
Age	<75	0.49	0.06, 0.93	92.7%	-	-	-
	>75	0.20	0.03, 0.38	0.0%	-	-	-
Setting	Hospitalized	0.28	0.11, 0.45	0.0%	-	-	-
	Community-dwelling	0.51	-0.01, 1.02	94.3%	-	-	-

		SMD	95% CI	I^2	SMD	95% CI	I^2
DC/LDC	DC	0.19	0.02, 0.37	0.0%	-	-	-
	LDC	0.49	0.06, 0.93	92.7%	-	-	-
Gender (male/female)	<1	0.50	0.02, 0.97	92.7%	-	-	-
	>1	0.2	-0.04, 0.44	52.6%	-	-	-
**CRP (26/7)**	Overall	0.35	0.06, 0.64	96.0%	1.71	0.20, 3.23	98.7%
Age	<75	0.51	0.18, 0.84	95%	0.29	-0.02, 0.61	63.6%
	>75	0.08	-0.49, 0.64	97.1%	3.71	-1.57, 8.99	99.4%
Setting	Hospitalized	0.58	0.27, 0.90	94.9%	3.71	-1.57, 8.99	99.4%
	Community-dwelling	-0.29	-0.85, 0.27	96.6%	0.29	-0.02, 0.61	63.6%
DC/LDC	DC	0.28	-0.13, 0.69	96.2%	3.71	-1.57, 8.99	99.4%
	LDC	0.42	-0.01, 0.86	96.1%	0.29	-0.02, 0.61	63.6%
Gender (male/female)	<1	0.24	-0.06, 0.55	95.1%	1.63	-0.53, 3.79	99.1%
	>1	0.58	-0.14, 1.31	97.4%	1.96	-1.44, 5.36	95.5%
**WBC (18/4)**	Overall	0.07	-0.19, 0.32	96.2%	1.48	0.11, 2.85	99.1%
Age	<75	0.04	-0.07, 0.16	76.4%	0.32	0.11, 0.52	65.2%
	>75	0.05	-1.29, 1.39	98.4%	2.67	-1.54, 6.88	99.4%
Setting	Hospitalized	0.20	-0.29, 0.69	96.8%	2.67	-1.54, 6.88	99.4%
	Community-dwelling	-0.20	-0.46, 0.06	93.8%	0.32	0.11, 0.52	65.2%
DC/LDC	DC	0.11	-0.83, 1.05	98.0%	2.67	-1.54, 6.88	99.4%
	LDC	0.01	-0.04, 0.06	3.0%	0.32	0.11, 0.52	65.2%
Gender (male/female)	<1	0.18	-0.16, 0.52	97.1%	-	-	-
	>1	-0.21	-0.51, 0.08	74.4%	-	-	-

		SMD	95% CI	I^2	SMD	95% CI	I^2
**Hematologic markers**							
**ALB (36/4)**	Overall	-0.58	-0.75, -0.42	94.2%	-1.33	-3.25, 0.60	99.1%
Age	<75	-0.40	-0.48, -0.32	67.7%	-0.22	-0.38, -0.05	0.0%
	>75	-0.90	-1.58, 0.22	97.4%	-2.41	-7.00, 2.18	99.5%
Setting	Hospitalized	-0.76	-1.08, -0.45	95.6%	-2.41	-7.00, 2.18	99.5%
	Community-dwelling	-0.28	-0.38, -0.18	71.0%	-0.22	-0.38, -0.05	0.0%
DC/LDC	DC	-0.76	-1.15, -0.36	96.4%	-2.41	-7.00, 2.18	99.5%
	LDC	-0.38	-0.48, -0.29	70.7%	-0.22	-0.38, -0.05	0.0%
Gender (male/female)	<1	-0.61	-0.82, -0.41	95.4%	-	-	-
	>1	-0.51	-0.77, -0.25	85.2%	-	-	-
**HB (23/3)**	Overall	-0.59	-0.85, -0.33	94.4%	0.09	-0.09, 0.27	32%
Age	<75	-0.40	-0.61, -0.20	86.1%	0.08	-0.08, 0.23	-
	>75	-0.76	-1.36, -0.16	96.4%	0.05	-0.39, 0.49	64.2%
Setting	Hospitalized	-0.63	-0.97, -0.29	95.2%	0.05	-0.39, 0.49	64.2%
	Community-dwelling	-0.44	-0.73, -0.15	83.25	0.08	-0.08, 0.23	-
DC/LDC	DC	-0.70	-1.16, 0.24	96.3%	0.05	-0.39, 0.49	64.2%
	LDC	-0.43	-0.64, -0.21	81.6%	0.08	-0.08, 0.23	-
Gender (male/female)	<1	-0.60	-1.01, -0.20	96.0%	0.05	-0.39, 0.49	64.2%
	>1	-0.62	-0.78, -0.45	55.9%	0.08	-0.08, 0.23	-
**Hormonal markers**							
**25(OH)D (13/5)**	Overall	-0.17	-0.30, -0.04	83.9%	-0.16	-0.22, -0.11	0.0%

		SMD	95% CI	I^2	SMD	95% CI	I^2
Age	<75	-0.11	-0.23, 0.01	80.2%	-0.18	-0.25, -0.10	13.6%
	>75	-0.35	-0.69, 0.00	87.8%	-0.12	-0.36, 0.11	-
Setting	Hospitalized	-0.41	-0.86, 0.04	90.0%	-	-	-
	Community-dwelling	-0.11	-0.22, 0.00	75.4%	-	-	-
DC/LDC	DC	-0.35	-0.75, 0.05	89.0%	-0.14	-0.21, -0.08	0.0%
	LDC	-0.14	-0.25, -0.02	78.6%	-0.23	-0.35, -0.12	0.0%
Gender (male/female)	<1	-0.14	-0.23, -0.04	68.3%	-0.15	-0.21, -0.08	0.0%
	>1	-0.58	-1.41, 0.25	95.6%	-0.23	-0.39, -0.07	28.8%
Insulin (9/4)	Overall	-0.70	-0.94, -0.45	93.5%	0.09	-0.14, 0.32	75.7%
Age	<75	-0.63	-0.86. -0.40	93.8%	-	-	-
	>75	-0.80	-2.72, 1.13	94.6%	-	-	-
Setting	Hospitalized	-0.80	-2.72, 1.13	94.6%	-	-	-
	Community-dwelling	-0.63	-0.86. -0.40	93.8%	-	-	-
DC/LDC	DC	-0.80	-2.72, 1.13	94.6%	0.20	0.13, 0.26	-
	LDC	-0.63	-0.86, -0.40	93.8%	0.04	-0.36, 0.45	74.1%
Gender (male/female)	<1	-0.62	-0.84, -0.40	9 2.6%	-	-	-
	>1	-0.97	-4.28, 2.34	97.0%	-	-	-

Abbreviations: CI: confidence interval; CRP: C-reactive protein; HDL: high-density lipoprotein; IGF-1: Insulin-like growth factor 1; IL-6: interleukin 6; LDL: low-density lipoprotein; SMD: standard mean difference; TNF-α: tumor necrosis factor alpha; ALB: albumin; WBC: white blood cell; HB: haemoglobin; DC: developed country; LDC: Least Developed Countries.

Supplementary S7: Forest plot of standardized mean differences

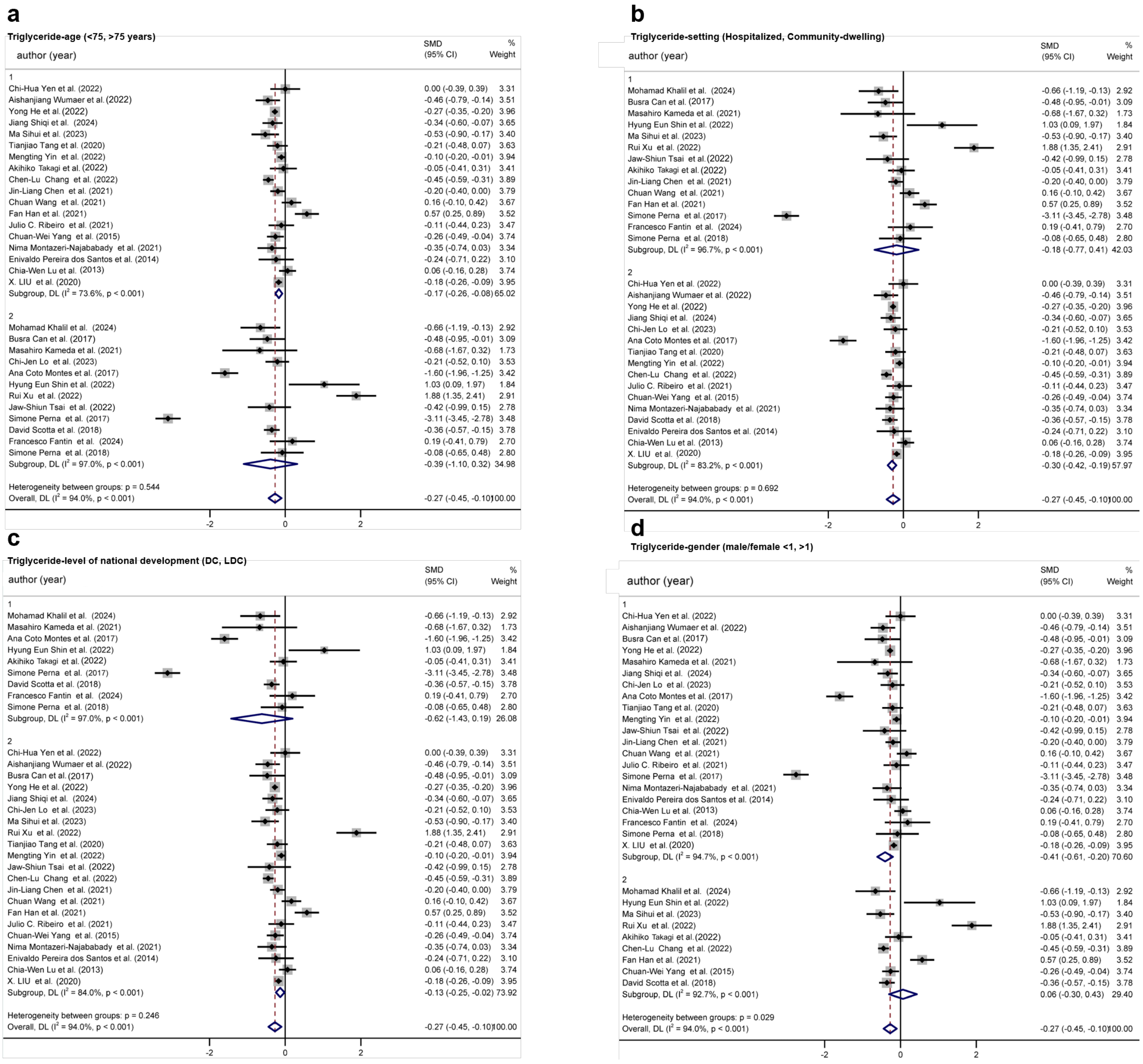

Figure S1: Forest plot of standardized mean differences for triglycerides in sarcopenia using random-effects model. a. Age subgroup; b. Setting subgroup; c. Level of national development subgroup; d. Gender subgroup. SMD, standardized mean differences; DC, Developed Countries; LDC, Less Developed Countries.

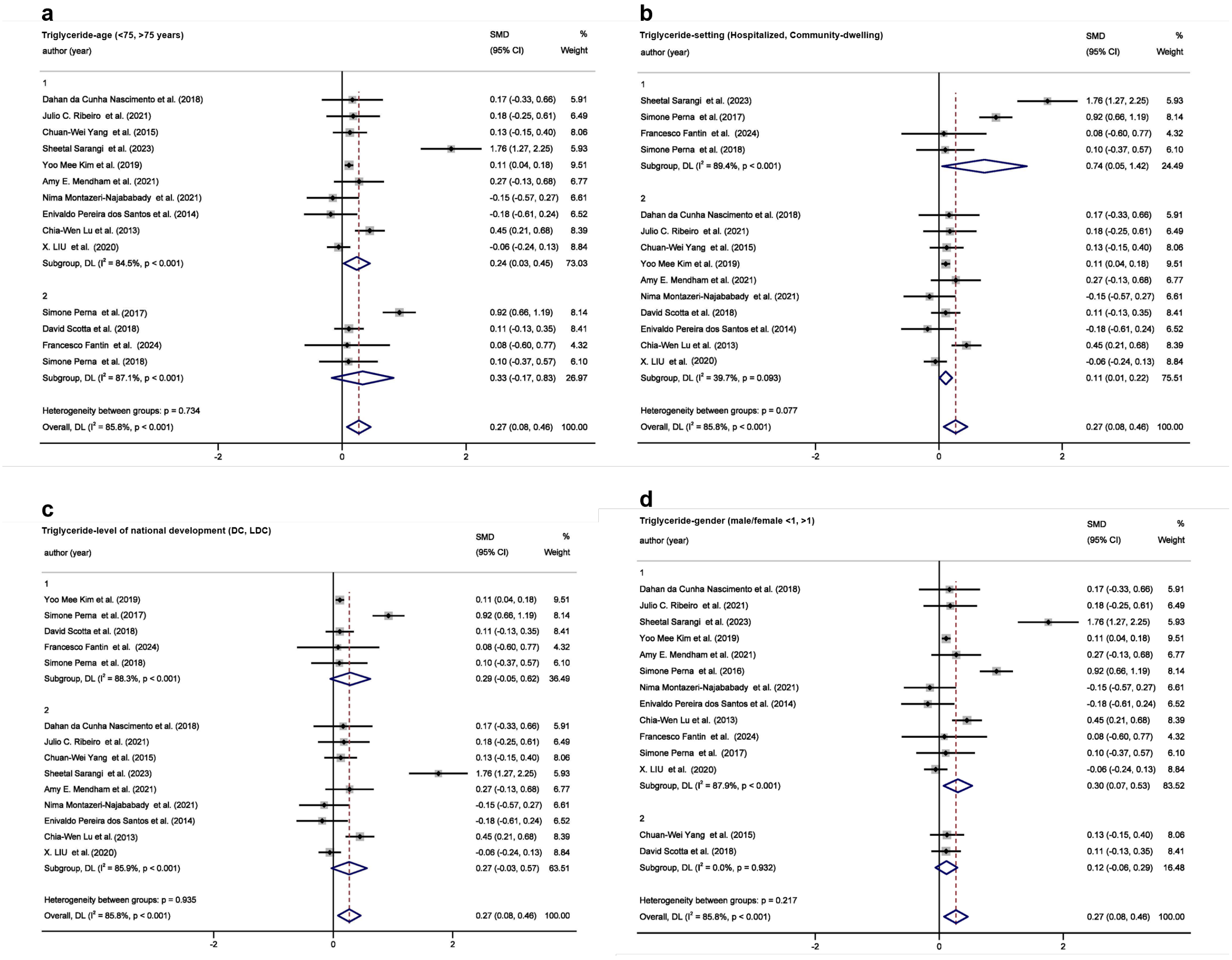

Figure S2: Forest plot of standardized mean differences for triglycerides in SO using random-effects model. a. Age subgroup; b. Setting subgroup; c. Level of national development subgroup; d. Gender subgroup. SMD, standardized mean differences; DC, Developed Countries; LDC, Less Developed Countries.

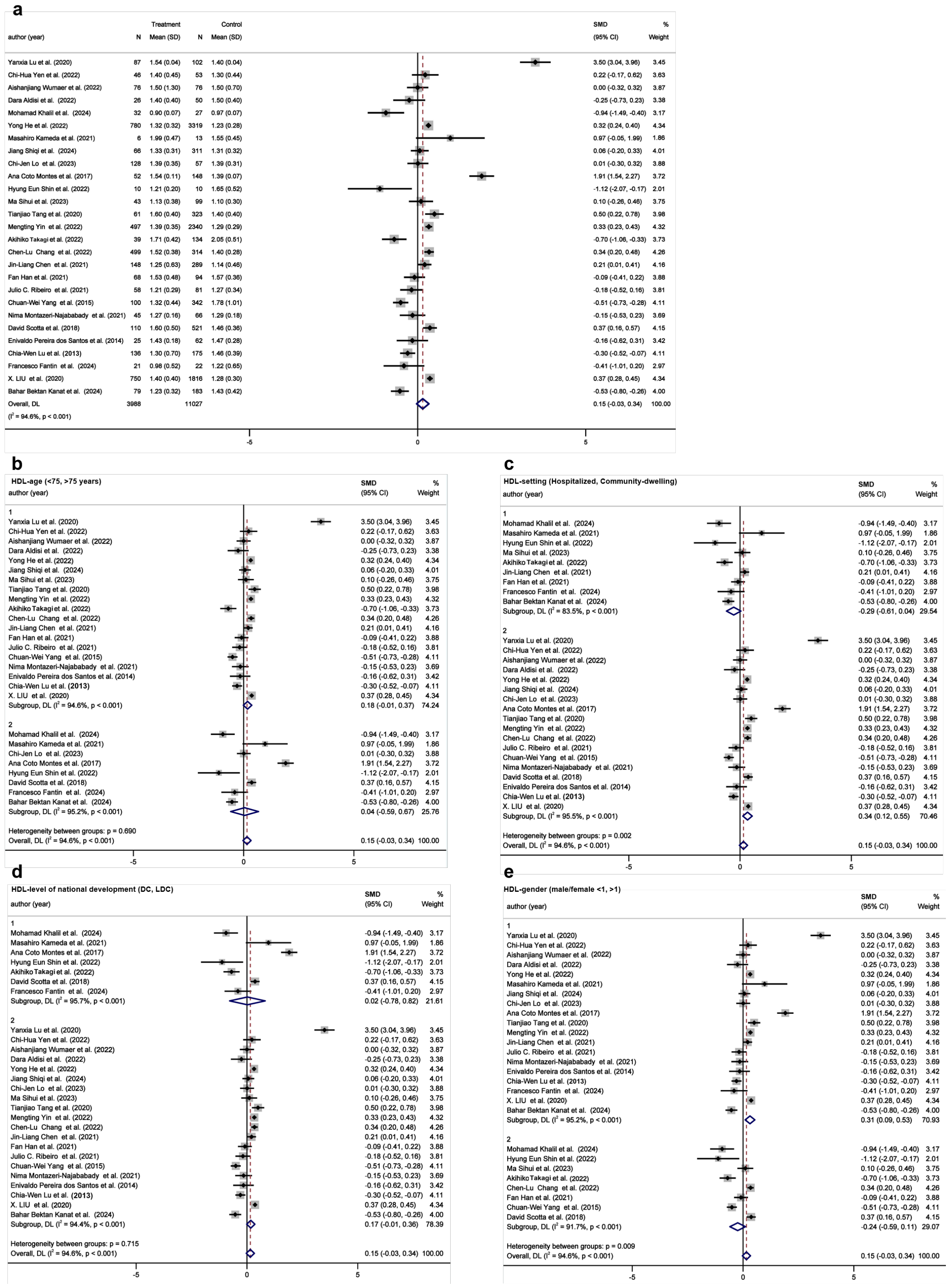

Figure S3: Forest plot of standardized mean differences for HDL in sarcopenia using random-effects model. a. Forest plot of HDL in sarcopenia; b. Age subgroup; c. Setting subgroup; d. Level of national development subgroup; e. Gender subgroup. SMD, standardized mean differences; DC, Developed Countries; LDC, Less Developed Countries.

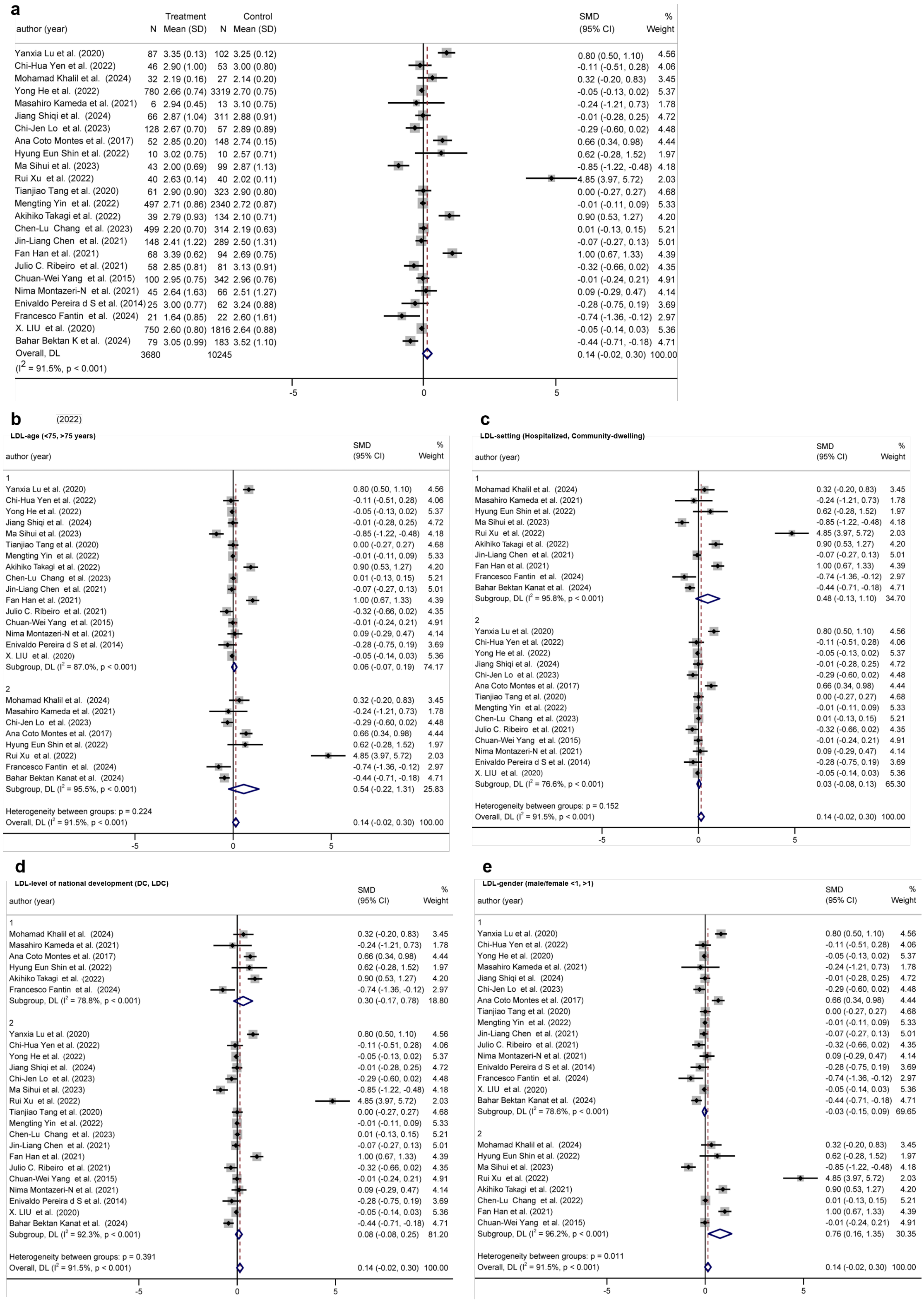

Figure S4: Forest plot of standardized mean differences for LDL in sarcopenia using random-effects model. a. Age subgroup; b. Setting subgroup; c. Level of national development subgroup; d. Gender subgroup. e. Gender subgroup. SMD, standardized mean differences; DC, Developed Countries; LDC, Less Developed Countries.

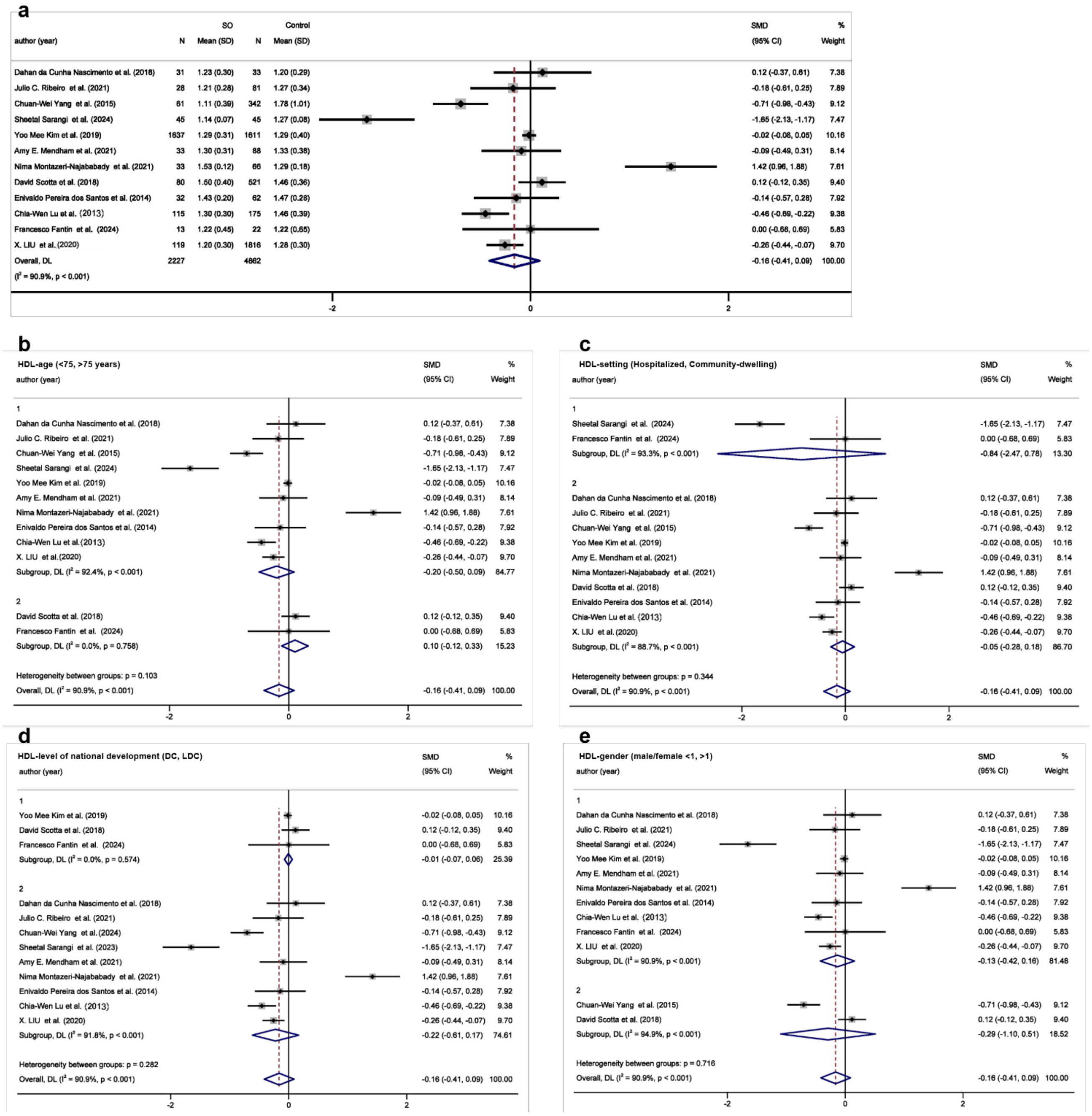

Figure S5: Forest plot of standardized mean differences for HDL in SO using random-effects model. a. Forest plot of HDL in SO; b. Age subgroup; c. Setting subgroup; d. Level of national development subgroup; e. Gender subgroup. SMD, standardized mean differences; DC, Developed Countries; LDC, Less Developed Countries.

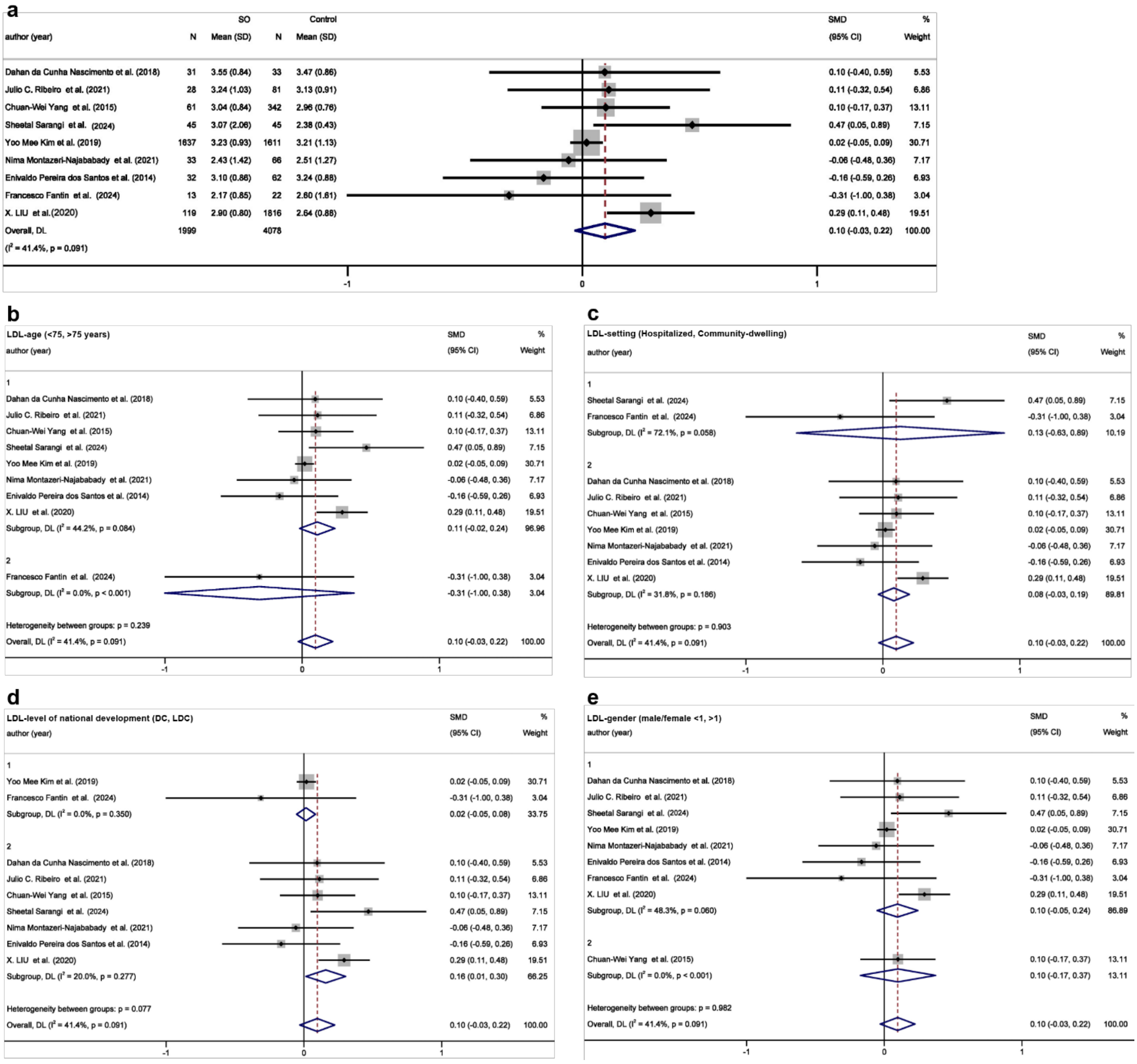

Figure S6: Forest plot of standardized mean differences for LDL in SO using random-effects model. a. Forest plot of LDL in SO; b. Age subgroup; c. Setting subgroup; d. Level of national development subgroup; e. Gender subgroup. SMD, standardized mean differences; DC, Developed Countries; LDC, Less Developed Countries.

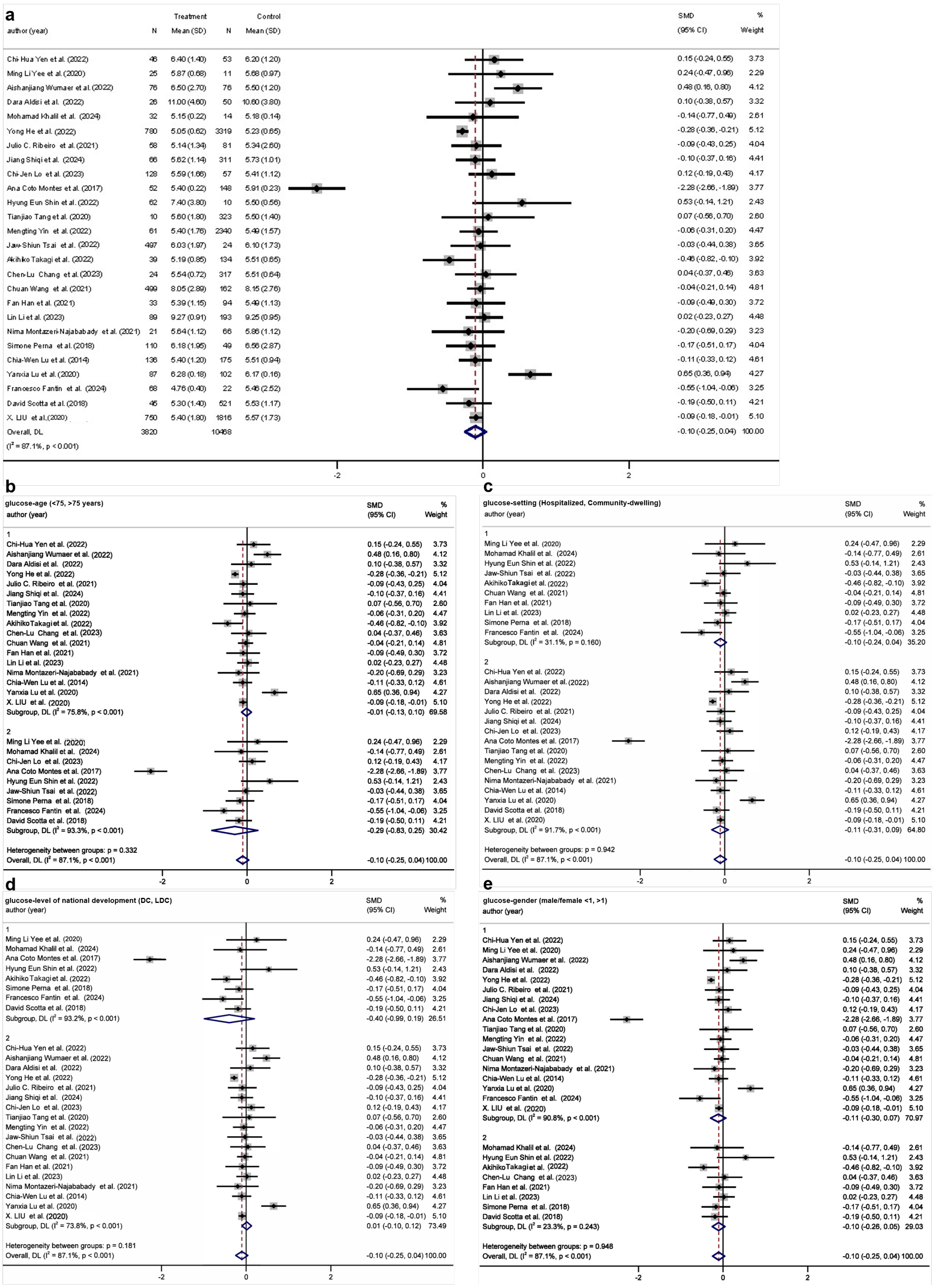

Figure S7: Forest plot of standardized mean differences for glucose in sarcopenia using random-effects model. a. Forest plot of glucose in sarcopenia; b. Age subgroup; c. Setting subgroup; d. Level of national development subgroup; e. Gender subgroup. SMD, standardized mean differences; DC, Developed Countries; LDC, Less Developed Countries.

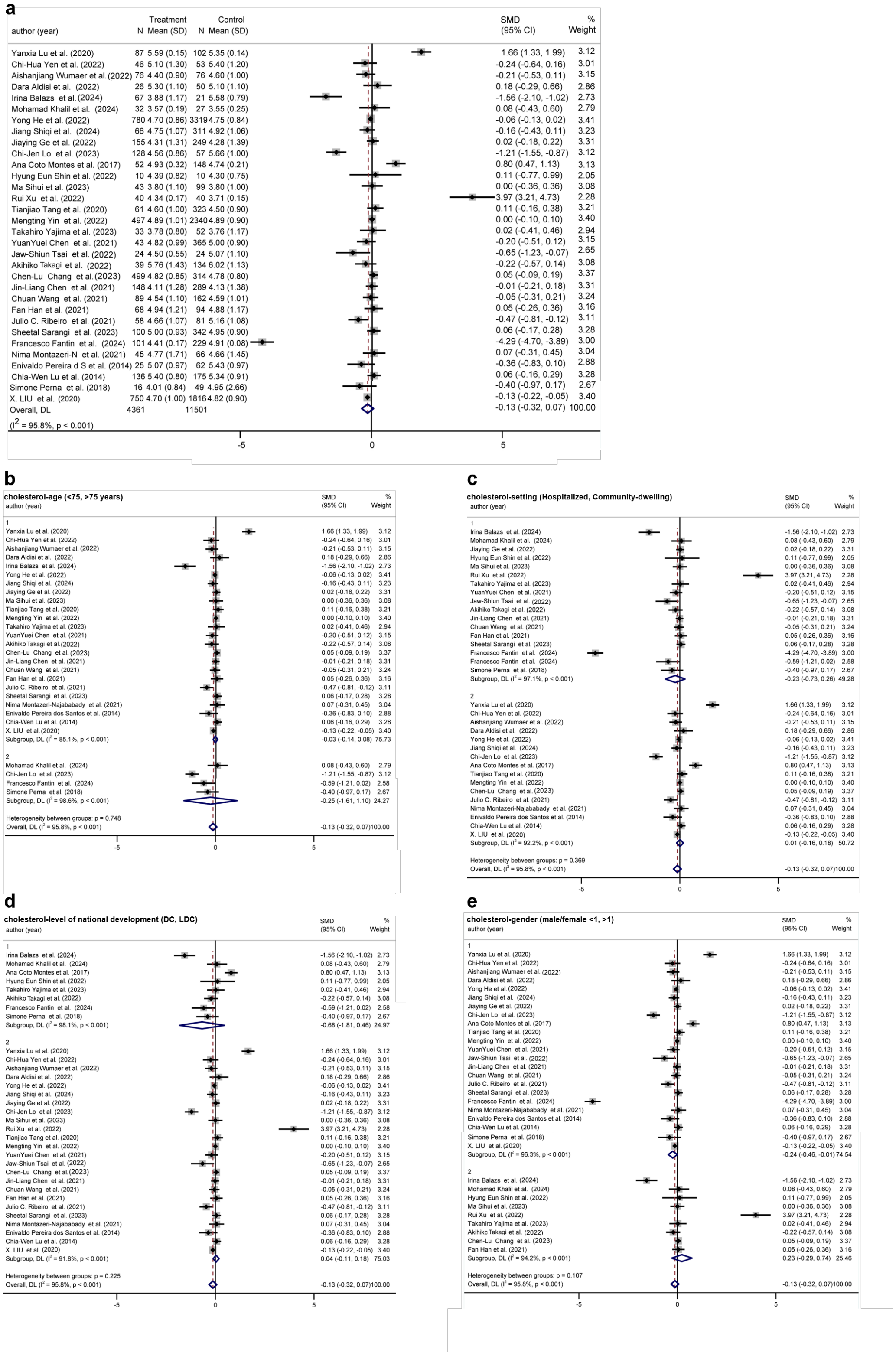

Figure S8: Forest plot of standardized mean differences for cholesterol in sarcopenia using random-effects model. a. Forest plot of standardized mean differences for cholesterol in sarcopenia; b. Age subgroup; c. Setting subgroup; d. Level of national development subgroup; e. Gender subgroup. SMD, standardized mean differences; DC, Developed Countries; LDC, Less Developed Countries.

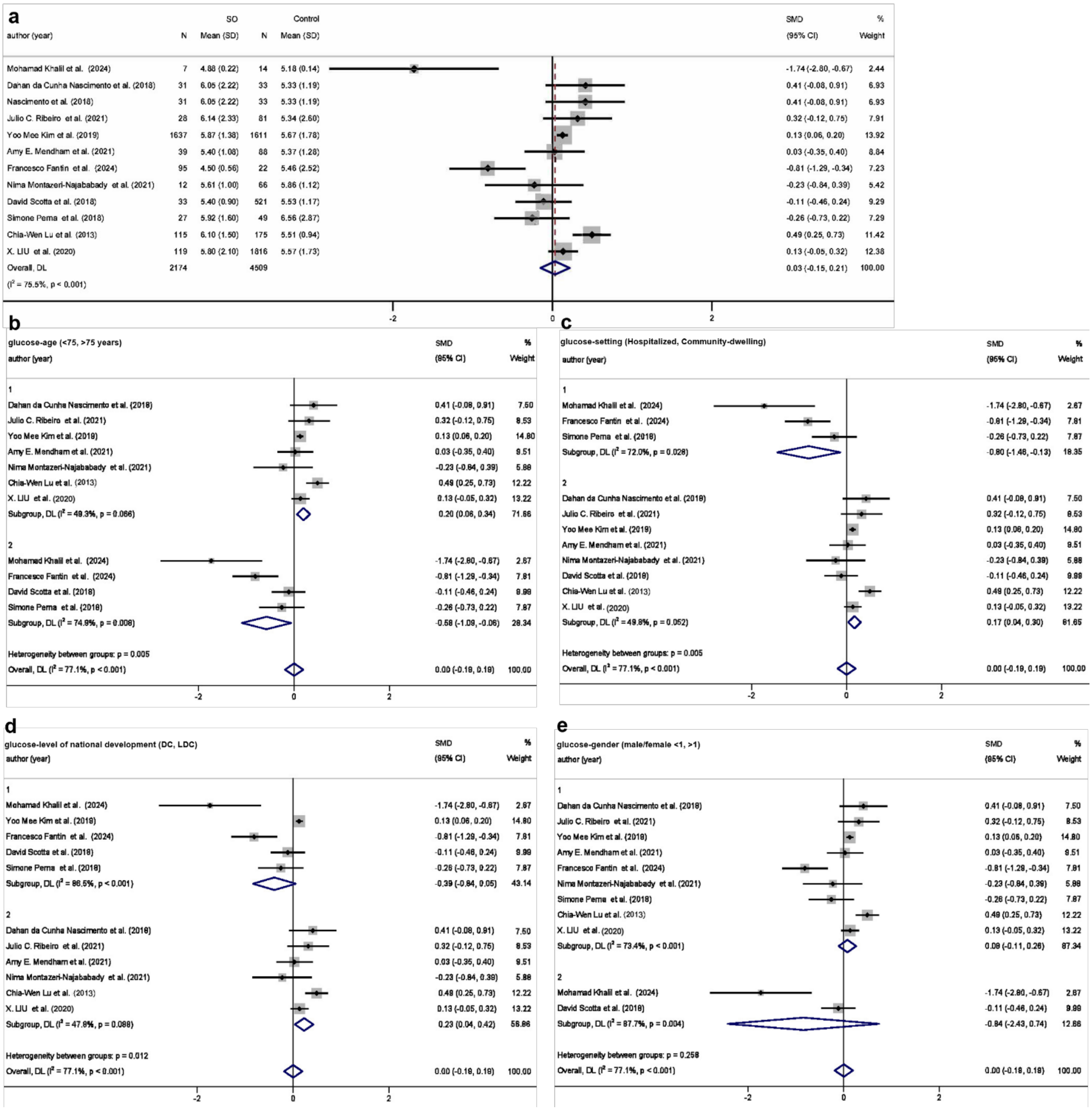

Figure S9: Forest plot of standardized mean differences for glucose in SO using random-effects model. a. Forest plot of glucose in SO; b. Age subgroup; c. Setting subgroup; d. Level of national development subgroup; e. Gender subgroup. SMD, standardized mean differences; DC, Developed Countries; LDC, Less Developed Countries.

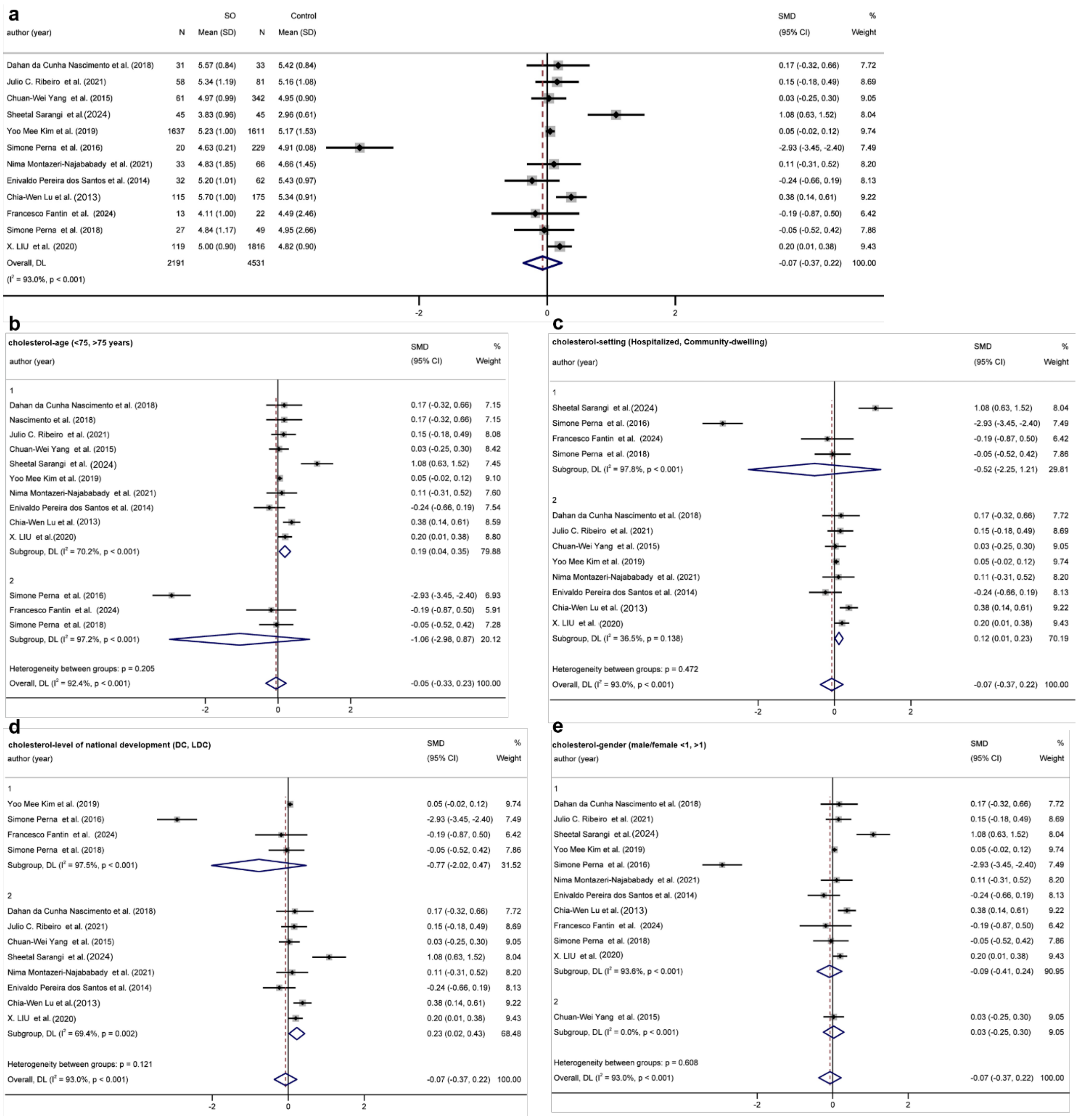

Figure S10: Forest plot of standardized mean differences for cholesterol in SO using random-effects model. a. Forest plot of cholesterol in SO; b. Age subgroup; c. Setting subgroup; d. Level of national development subgroup; e. Gender subgroup. SMD, standardized mean differences; DC, Developed Countries; LDC, Less Developed Countries.

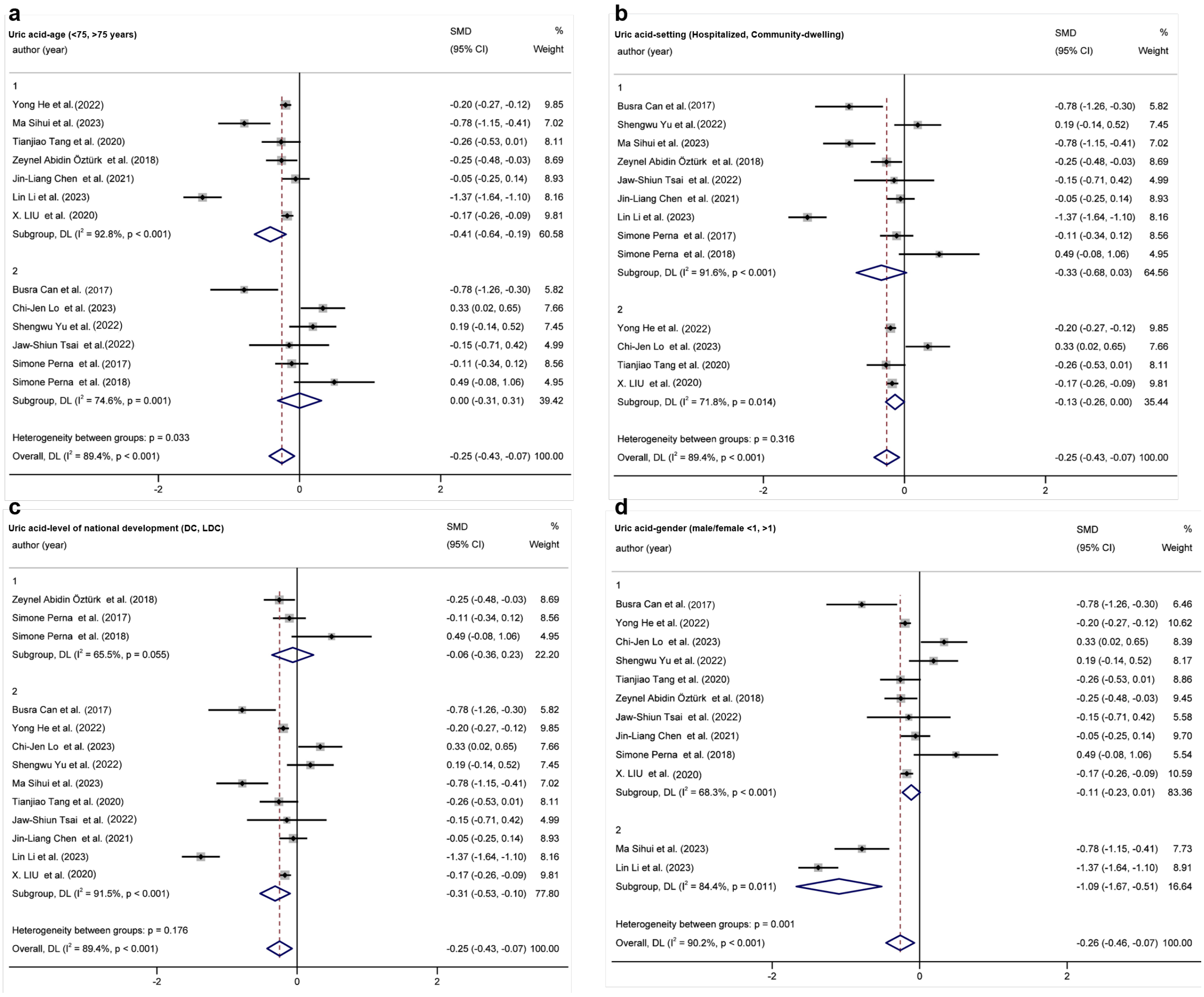

Figure S11: Forest plot of standardized mean differences for uric acid in sarcopenia using random-effects model. a. Age subgroup; b. Setting subgroup; c. Level of national development subgroup; d. Gender subgroup. SMD, standardized mean differences; DC, Developed Countries; LDC, Less Developed Countries.

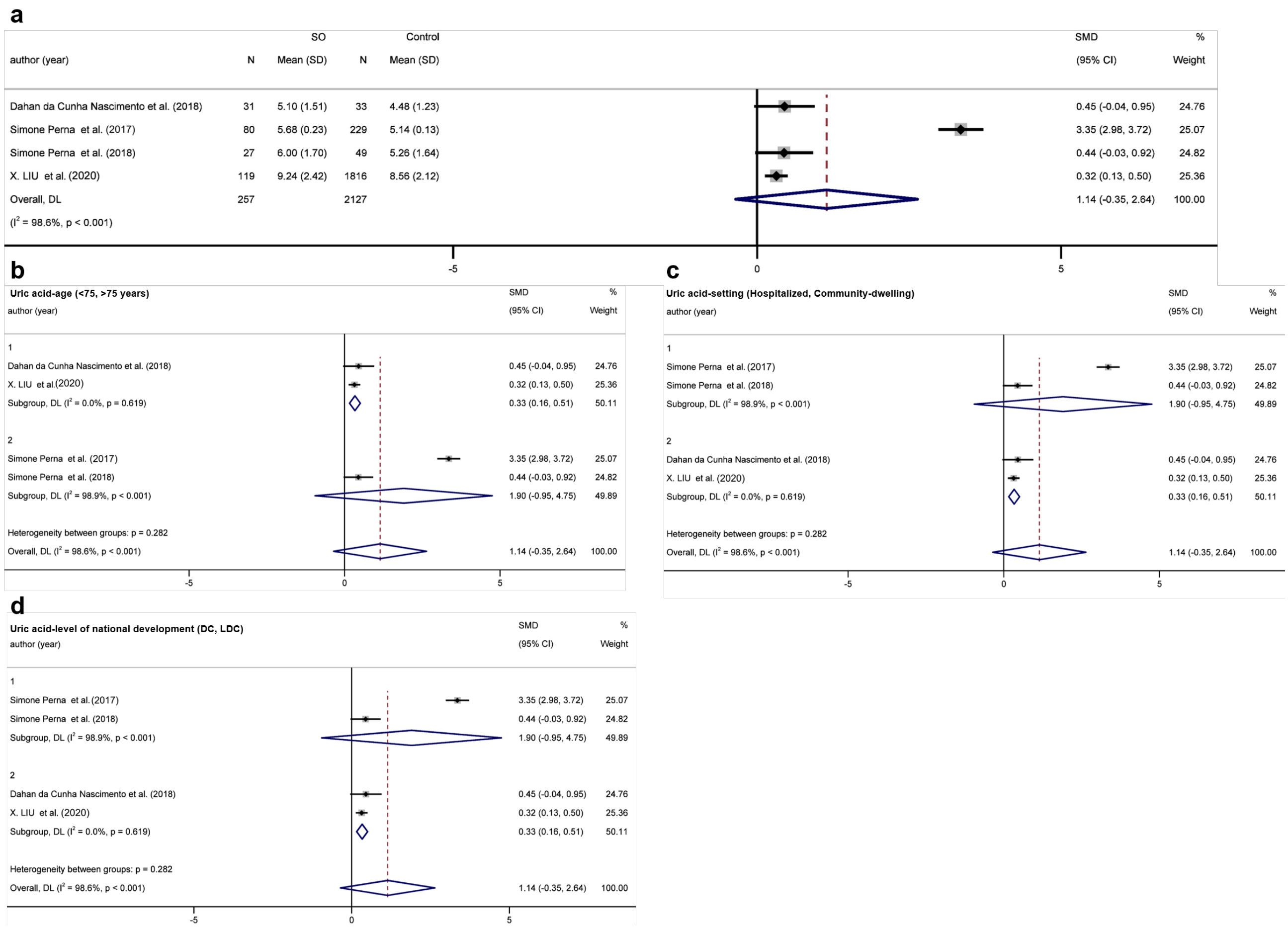

Figure S12: Forest plot of standardized mean differences for uric acid in SO using random-effects model. a. Forest plot of standardized mean differences for uric acid in SO; b. Forest plot of uric acid in SO; c. Age subgroup; d. Setting subgroup; SMD, standardized mean differences; DC, Developed Countrie; LDC, Less Developed Countries.

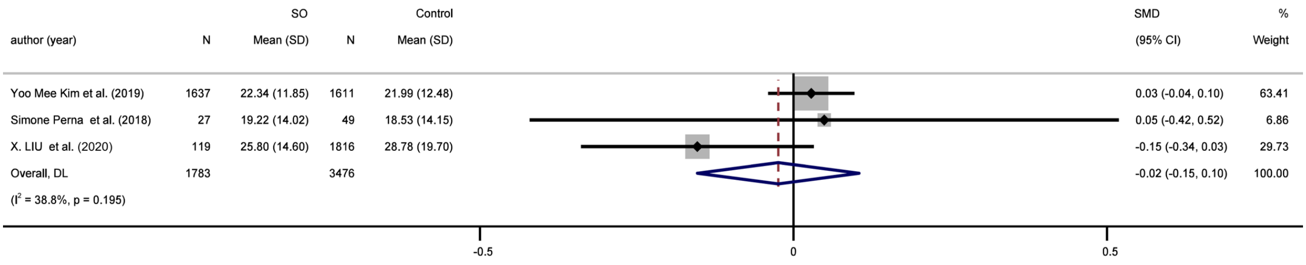

Figure S13: Forest plot of standardized mean differences for ALT in SO using random-effects model. SMD, standardized mean differences; DC, Developed Countries; LDC, Less Developed Countries.

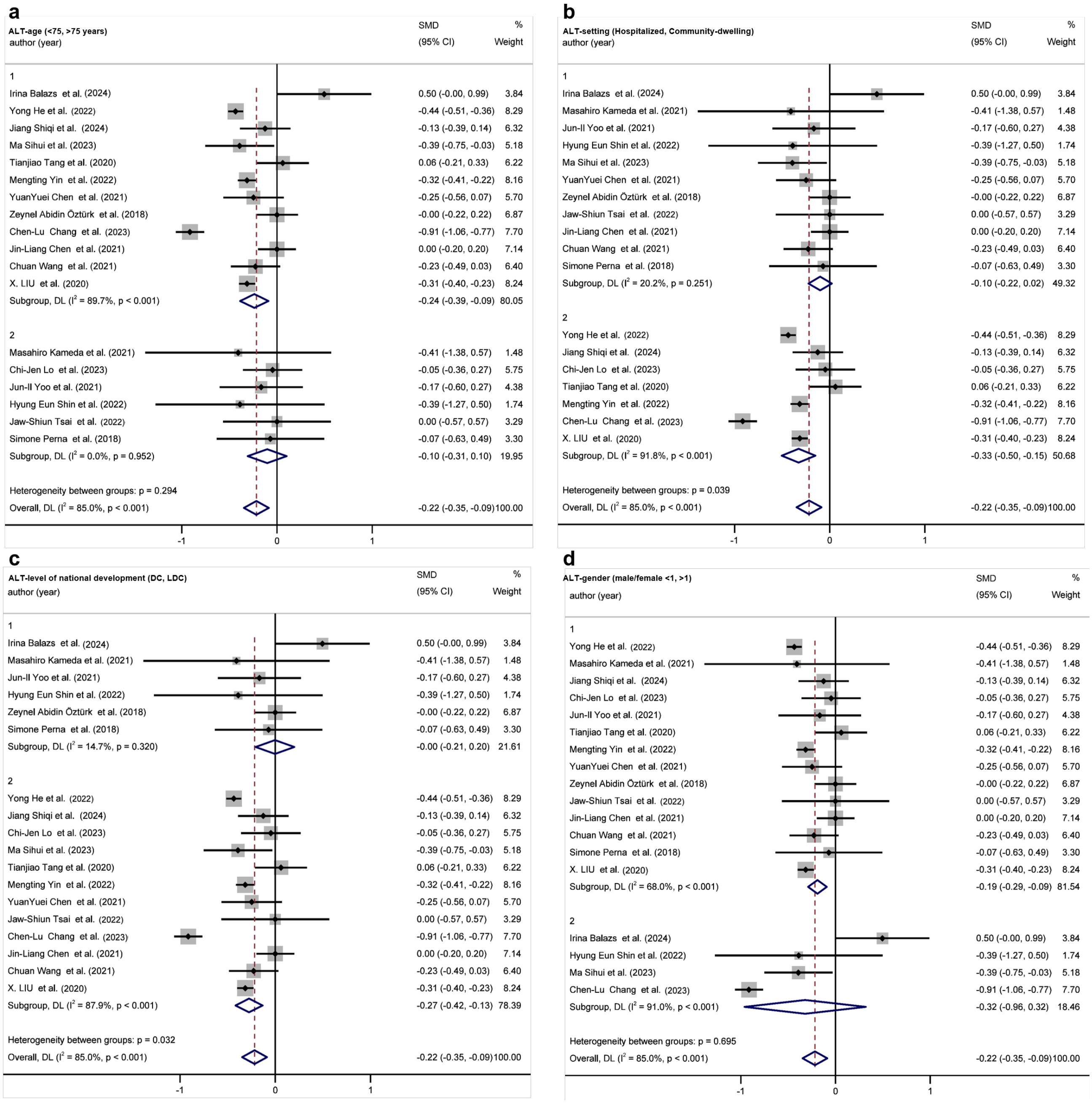

Figure S14: Forest plot of standardized mean differences for ALT in Sarcopenia using random-effects model. a. Age subgroup; b. Setting subgroup; c. Level of national development subgroup; d. Gender subgroup. SMD, standardized mean differences; DC, Developed Countries; LDC, Less Developed Countries.

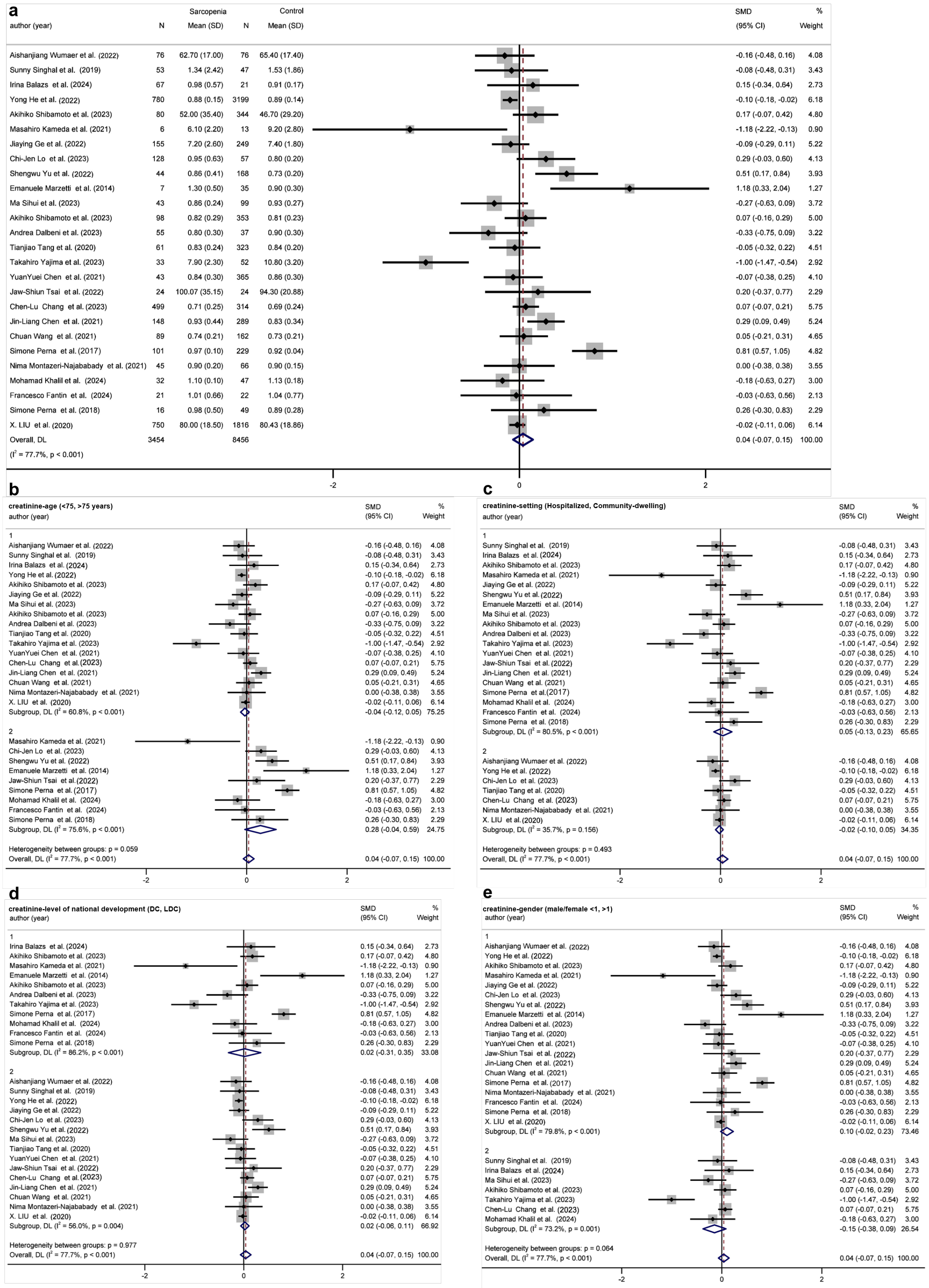

Figure S15: Forest plot of standardized mean differences for Creatinine in sarcopenia using random-effects model. a. Forest plot of Creatinine in sarcopenia; b. Age subgroup; c. Setting subgroup; d. Level of national development subgroup; e. Gender subgroup. SMD, standardized mean differences; DC, Developed Countries;

LDC, Less Developed Countries.

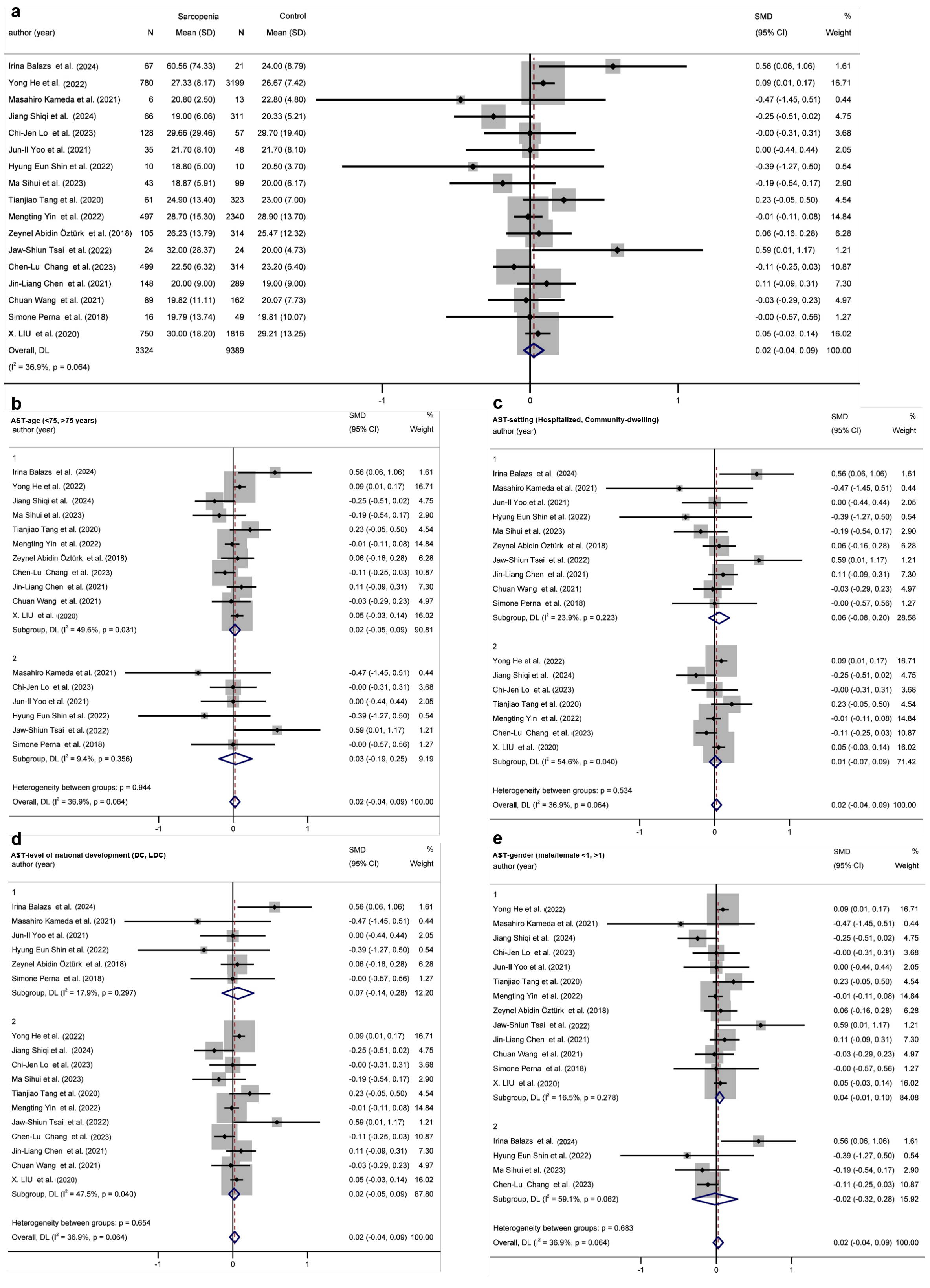

Figure S16: Forest plot of standardized mean differences for AST in sarcopenia using random-effects model.

a. Forest plot of AST in sarcopenia; b. Age subgroup; c. Setting subgroup; d. Level of national development

subgroup; e. Gender subgroup. SMD, standardized mean differences; DC, Developed Countries; LDC, Less Developed Countries.

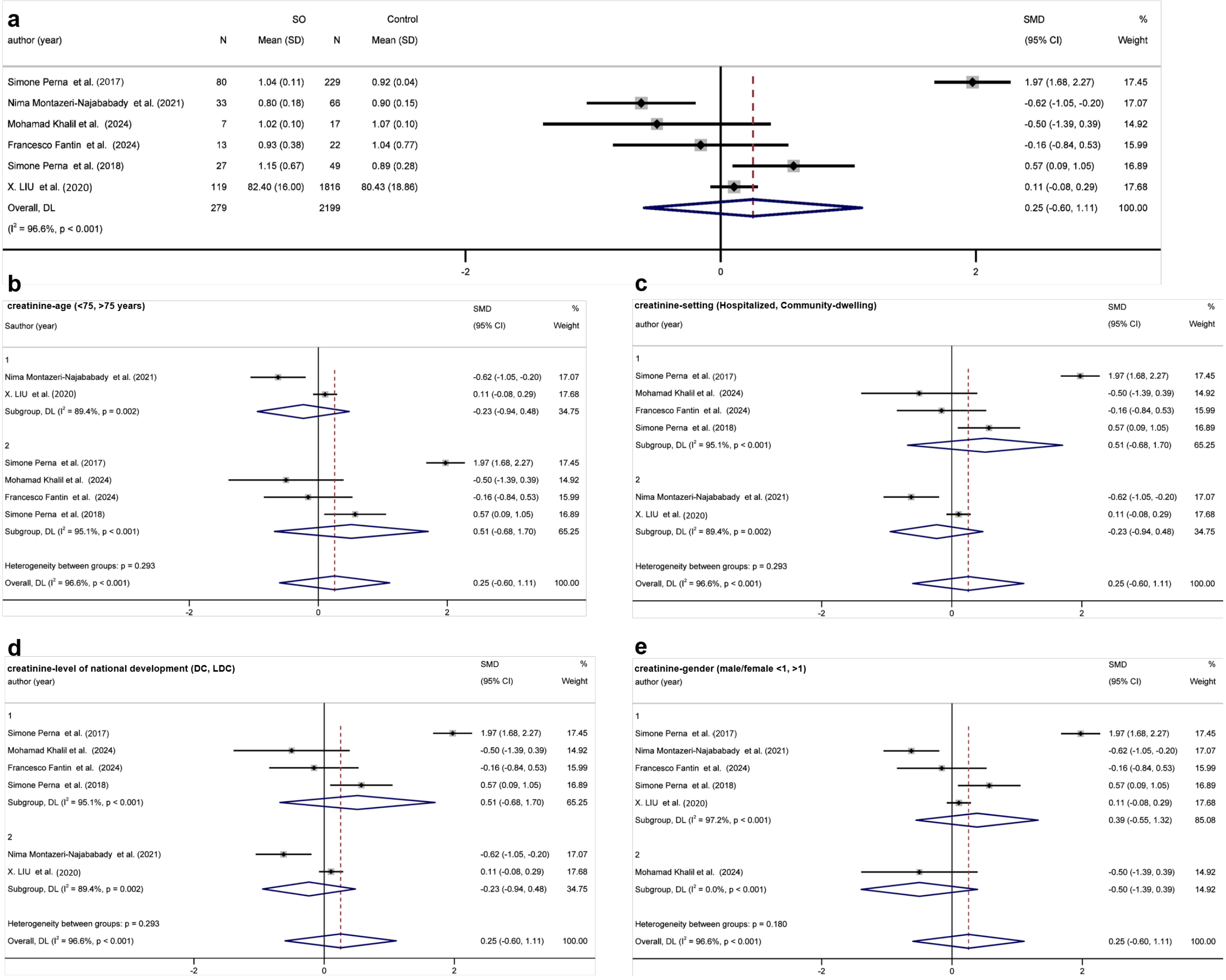

Figure S17: Forest plot of standardized mean differences for Creatinine in SO using random-effects model. a. Forest plot of Creatinine in SO. b. Age subgroup; c. Setting subgroup; d. Level of national development subgroup; e. Gender subgroup. SMD, standardized mean differences; DC, Developed Countries; LDC, Less Developed Countries.

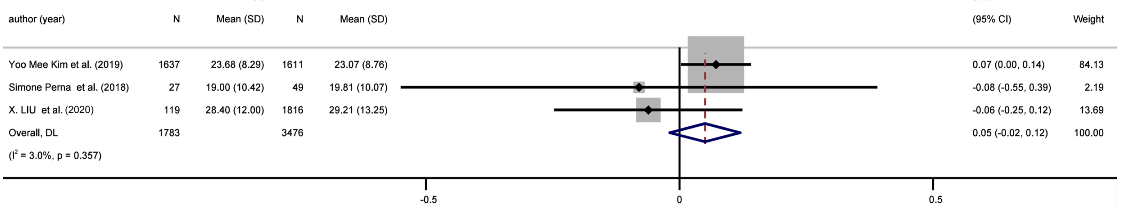

Figure S18: Forest plot of standardized mean differences for AST in SO using random-effects model. SMD, standardized mean differences; DC, Developed Countries; LDC, Less Developed Countries.

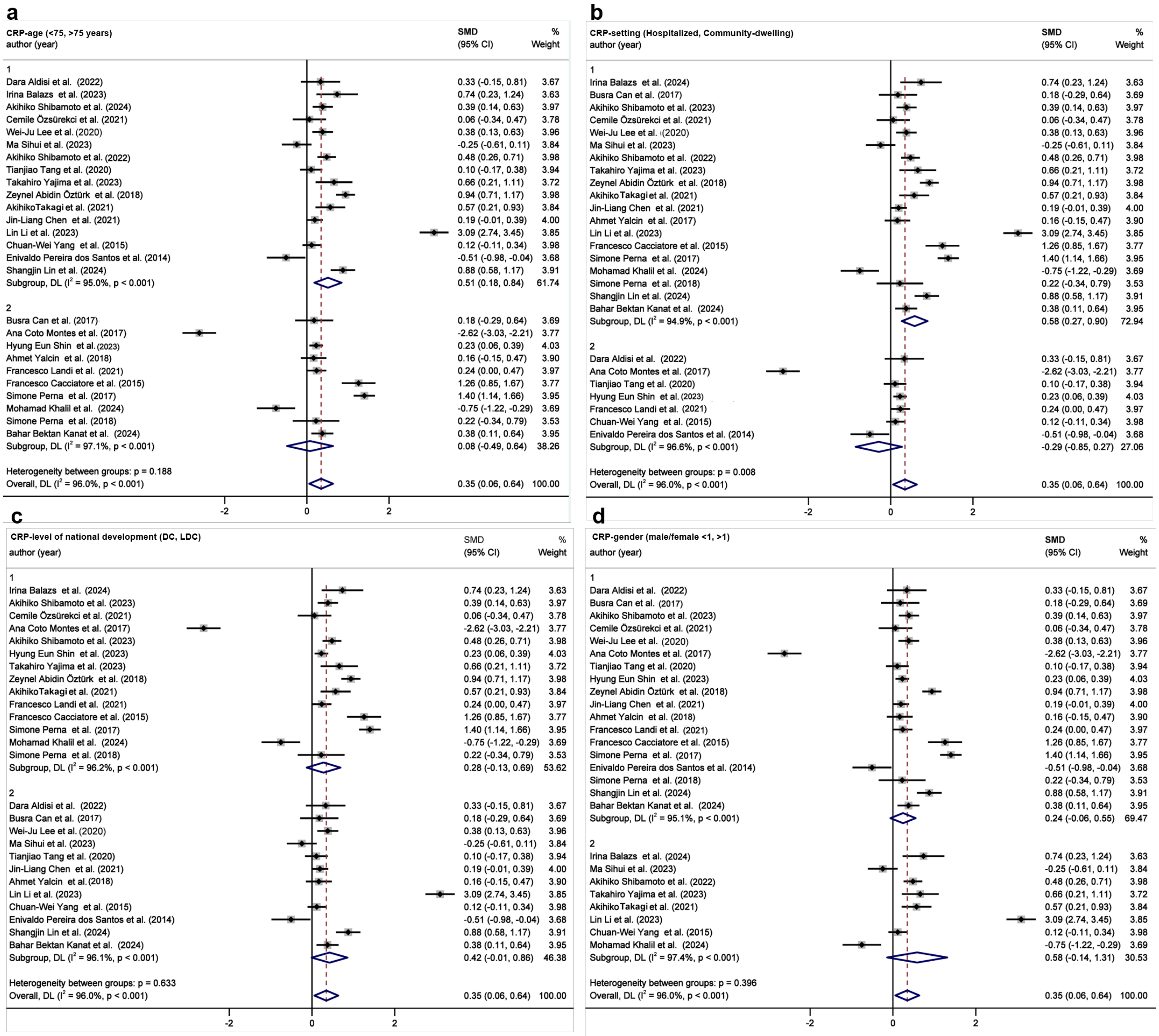

Figure S19: Forest plot of standardized mean differences for CRP in sarcopenia using random-effects model. a. Age subgroup; b. Setting subgroup; c. Level of national development subgroup; d. Gender subgroup. SMD, standardized mean differences; DC, Developed Countries; LDC, Less Developed Countries.

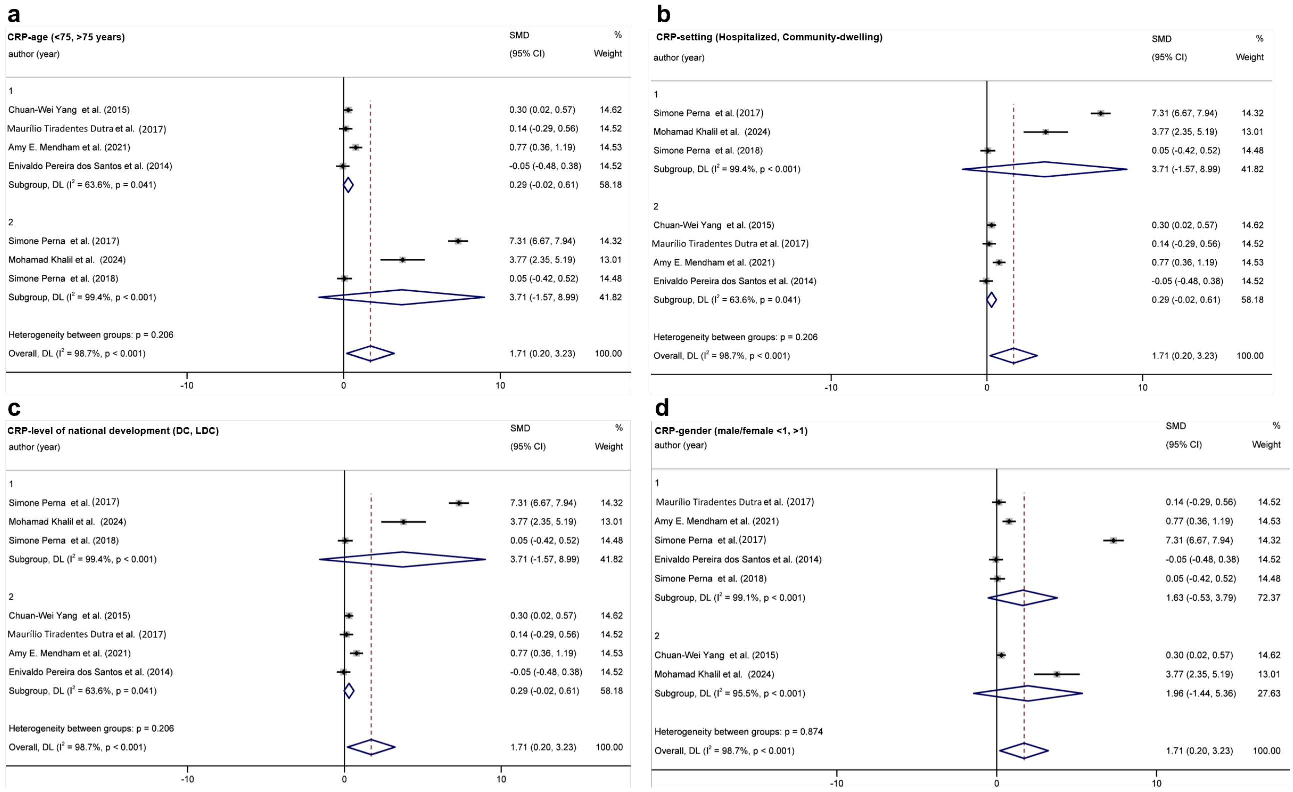

Figure S20: Forest plot of standardized mean differences for CRP in SO using random-effects model. a. Age subgroup; b. Setting subgroup; c. Level of national development subgroup; d. Gender subgroup. SMD, standardized mean differences; DC, Developed Countries; LDC, Less Developed Countries.

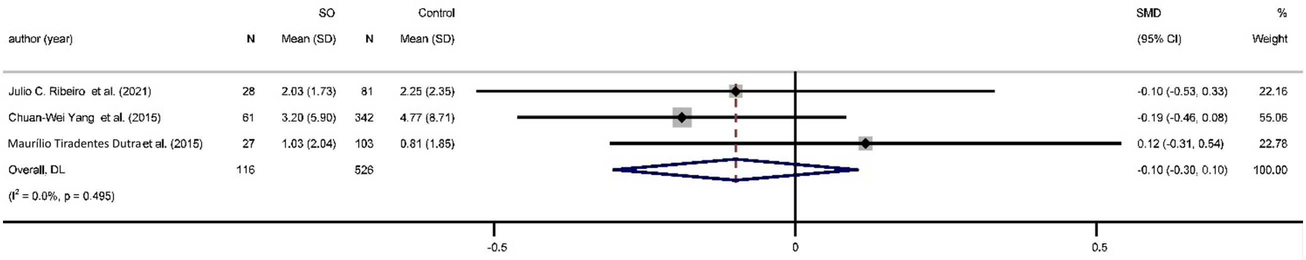

Figure S21: Forest plot of standardized mean differences for TNF-α in SO using random-effects model. SMD, standardized mean differences; DC, Developed Countries; LDC, Less Developed Countries.

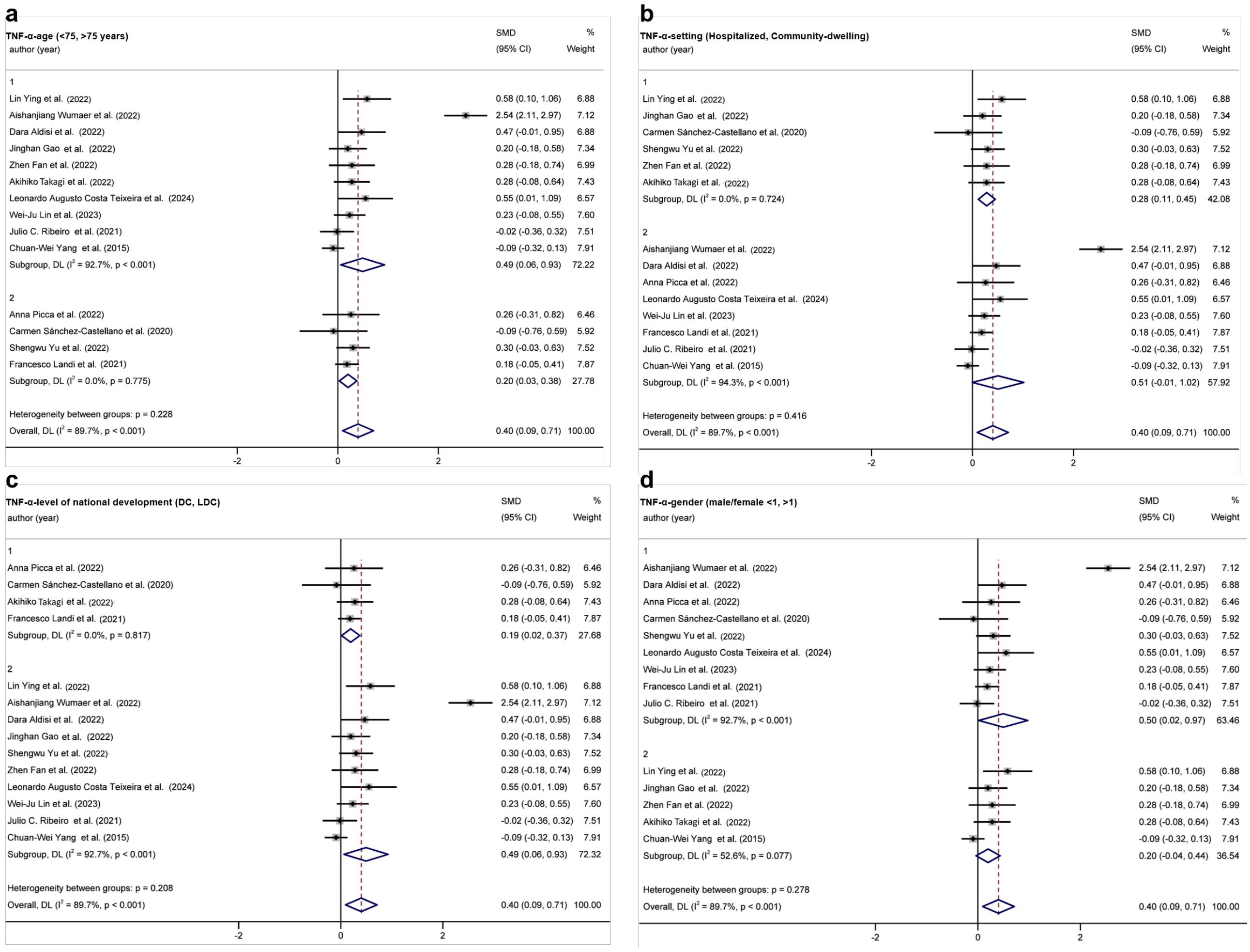

Figure S22: Forest plot of standardized mean differences for TNF-α in Sarcopenia using random-effects model. a. Age subgroup; b. Setting subgroup; c. Level of national development subgroup; d. Gender subgroup. SMD, standardized mean differences; DC, Developed Countries; LDC, Less Developed Countries.

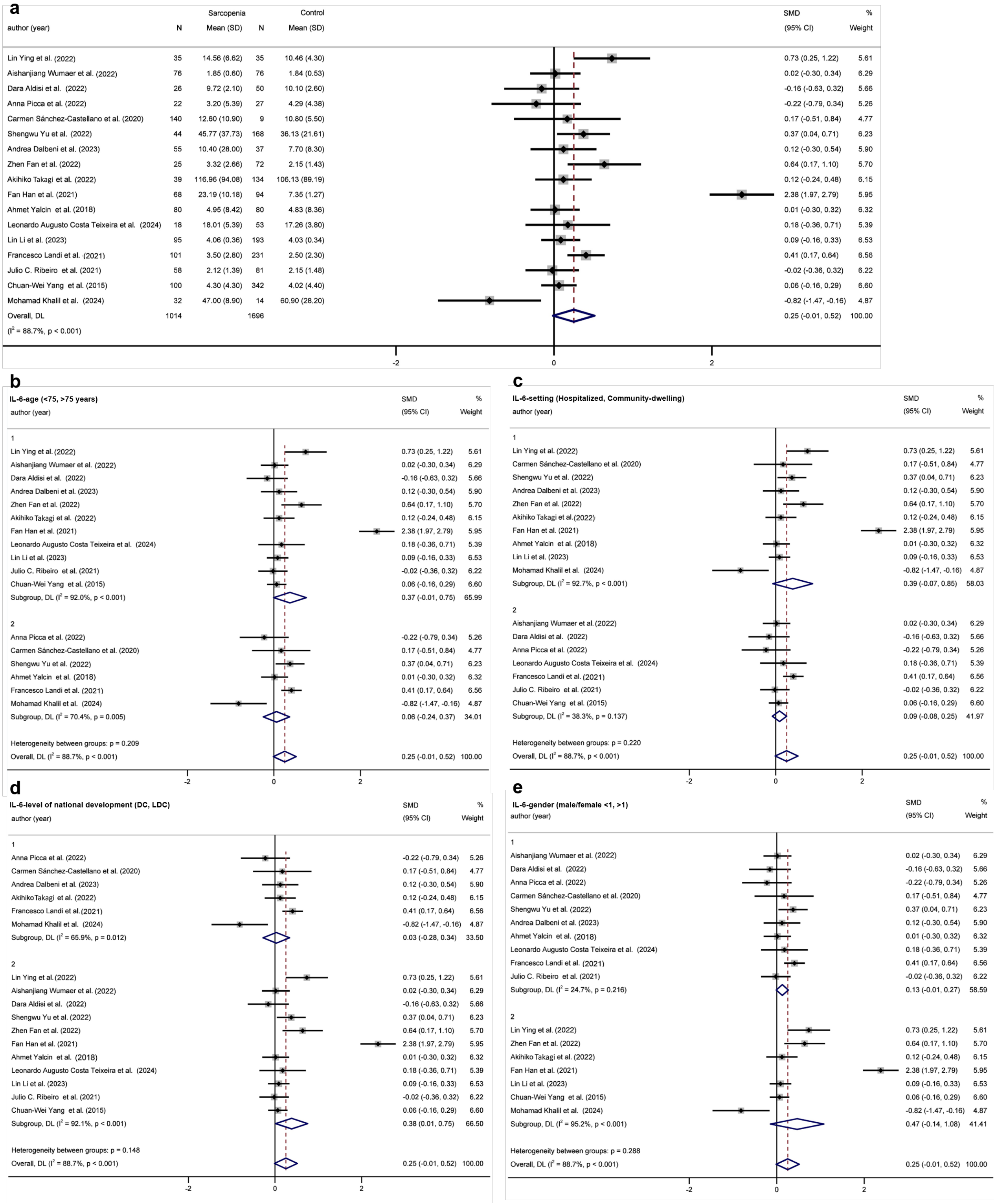

Figure S23: Forest plot of standardized mean differences for IL-6 in sarcopenia using random-effects model. a. Forest plot of IL-6 in sarcopenia; b. Age subgroup; c. Setting subgroup; d. Level of national development subgroup; e. Gender subgroup. SMD, standardized mean differences; DC, Developed Countries; LDC, Less Developed Countries.

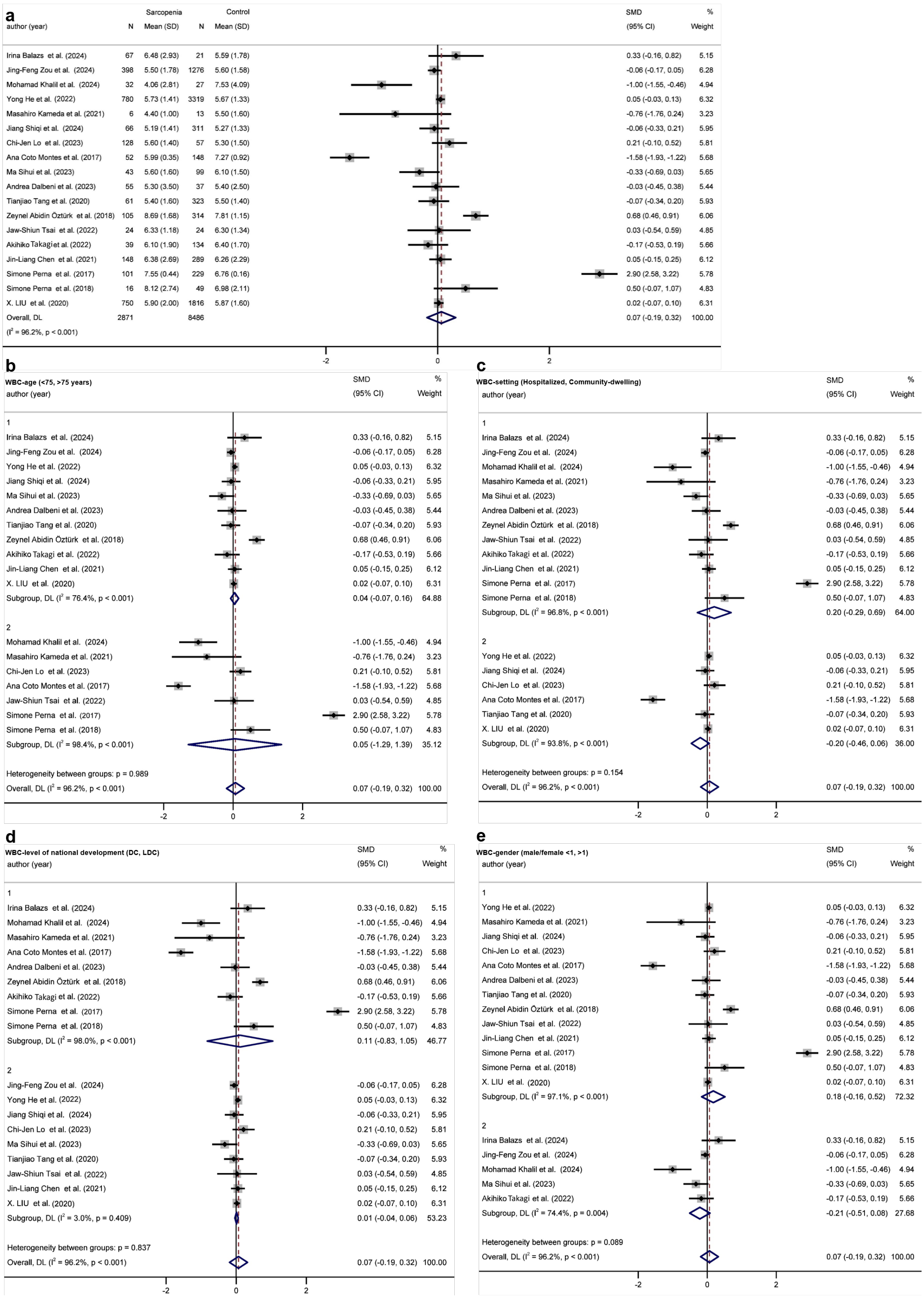

Figure S24: Forest plot of standardized mean differences for WBC in sarcopenia using random-effects model. a. Forest plot of WBC in sarcopenia; b. Age subgroup; c. Setting subgroup; d. Level of national development subgroup; e. Gender subgroup. SMD, standardized mean differences; DC, Developed Countries; LDC, Less

Developed Countries.

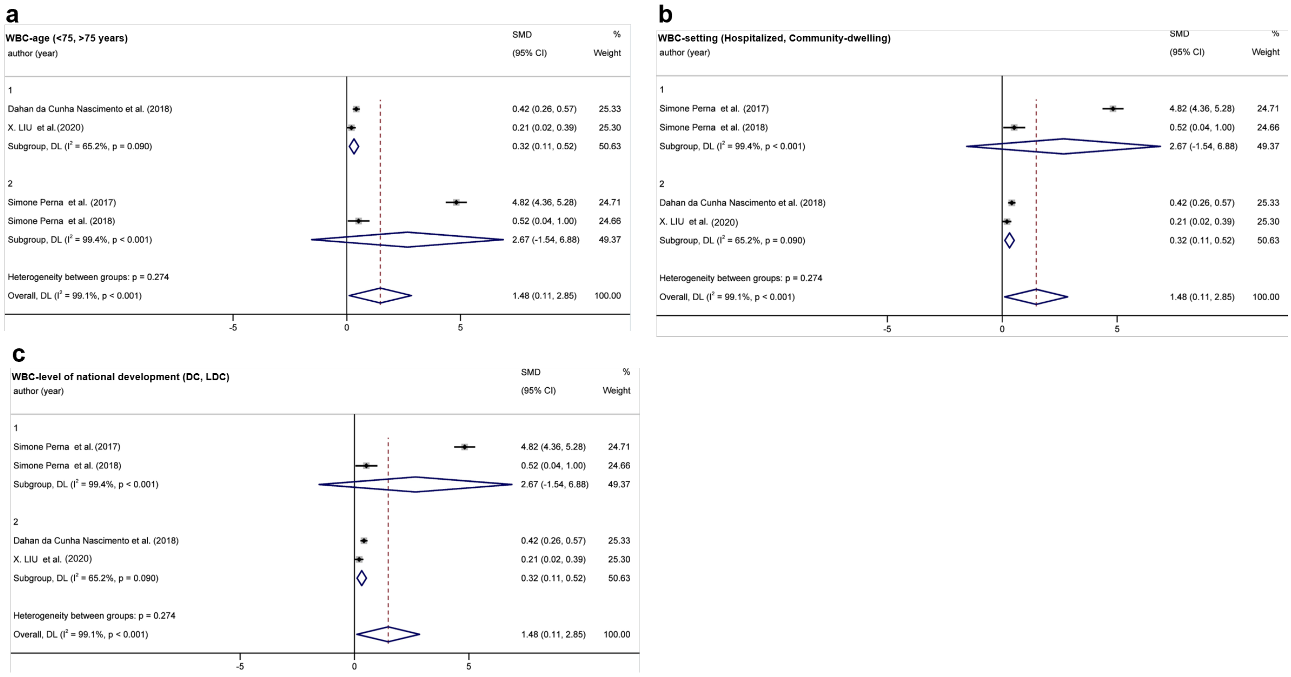

Figure S25: Forest plot of standardized mean differences for WBC in SO using random-effects model. a. Age subgroup; b. Setting subgroup; c. Level of national development subgroup; SMD, standardized mean differences; DC, Developed Countries; LDC, Less Developed Countries.

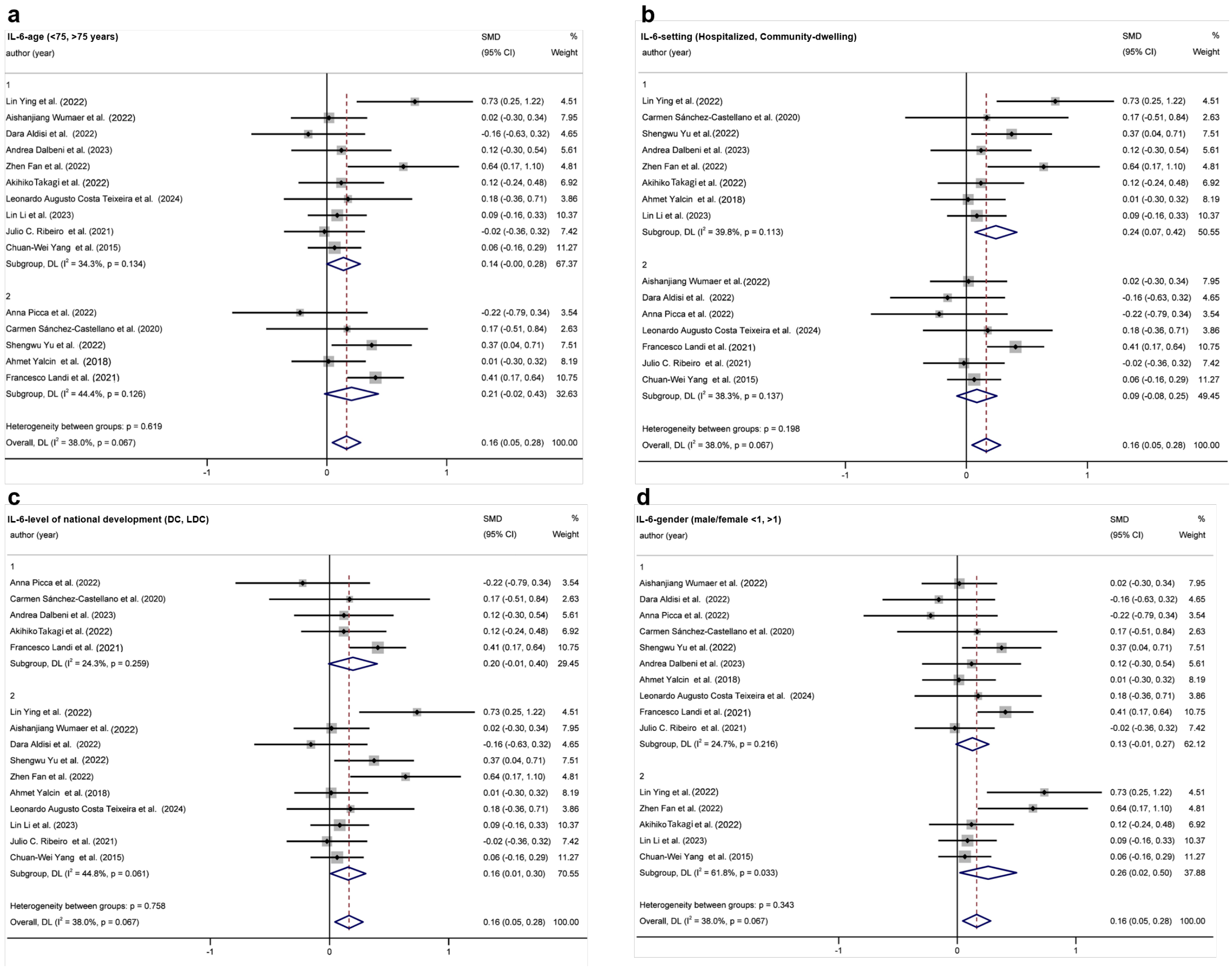

Figure S26: Forest plot of standardized mean differences for IL-6 in sarcopenia using random-effects model (After removing high-heterogeneity studies). a. Age subgroup; b. Setting subgroup; c. Level of national development subgroup; d. Gender subgroup. SMD, standardized mean differences; DC, Developed Countries; LDC, Less Developed Countries.

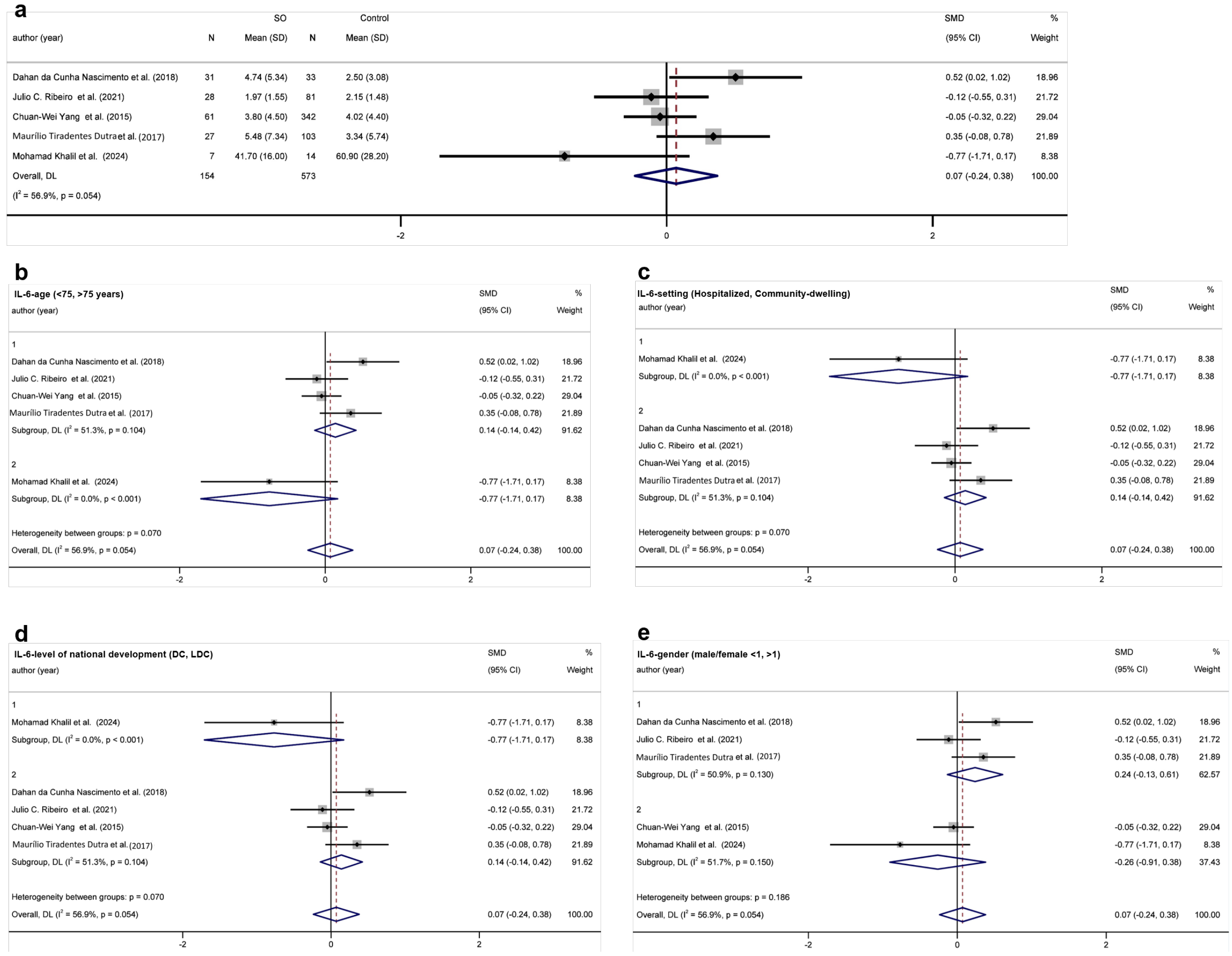

Figure S27: Forest plot of standardized mean differences for IL-6 in SO using random-effects model (After removing high-heterogeneity studies). a. Forest plot of IL-6 in SO; b. Age subgroup; c. Setting subgroup; d. Level of national development subgroup; e. Gender subgroup. SMD, standardized mean differences; DC, Developed Countries; LDC, Less Developed Countries.

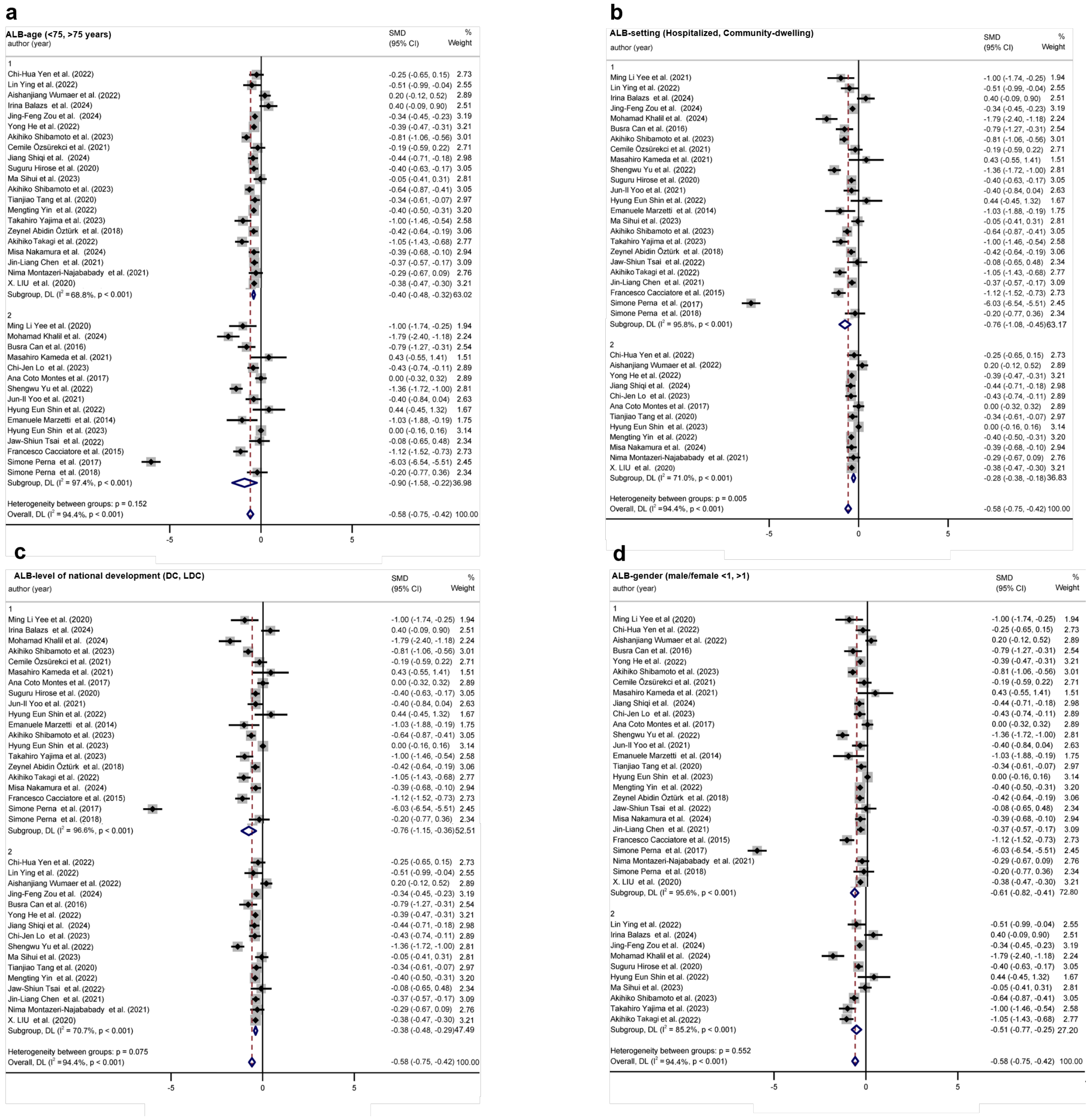

Figure S28: Forest plot of standardized mean differences for ALB in sarcopenia using random-effects model. a. Age subgroup; b. Setting subgroup; c. Level of national development subgroup; d. Gender subgroup. SMD, standardized mean differences; DC, Developed Countries; LDC, Less Developed Countries.

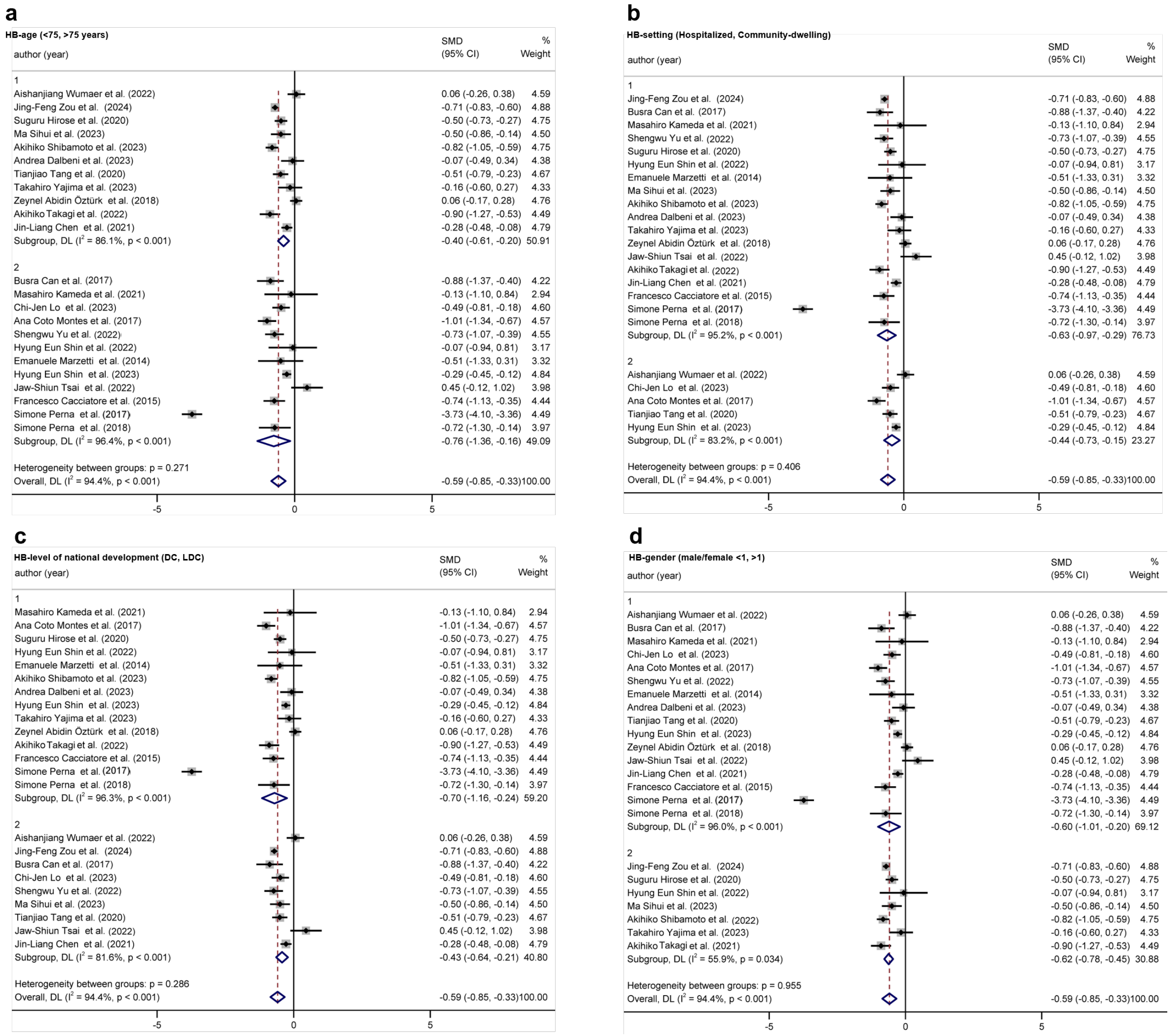

Figure S29: Forest plot of standardized mean differences for HB in sarcopenia using random-effects model. a. Age subgroup; b. Setting subgroup; c. Level of national development subgroup; d. Gender subgroup. SMD, standardized mean differences; DC, Developed Countries; LDC, Less Developed Countries.

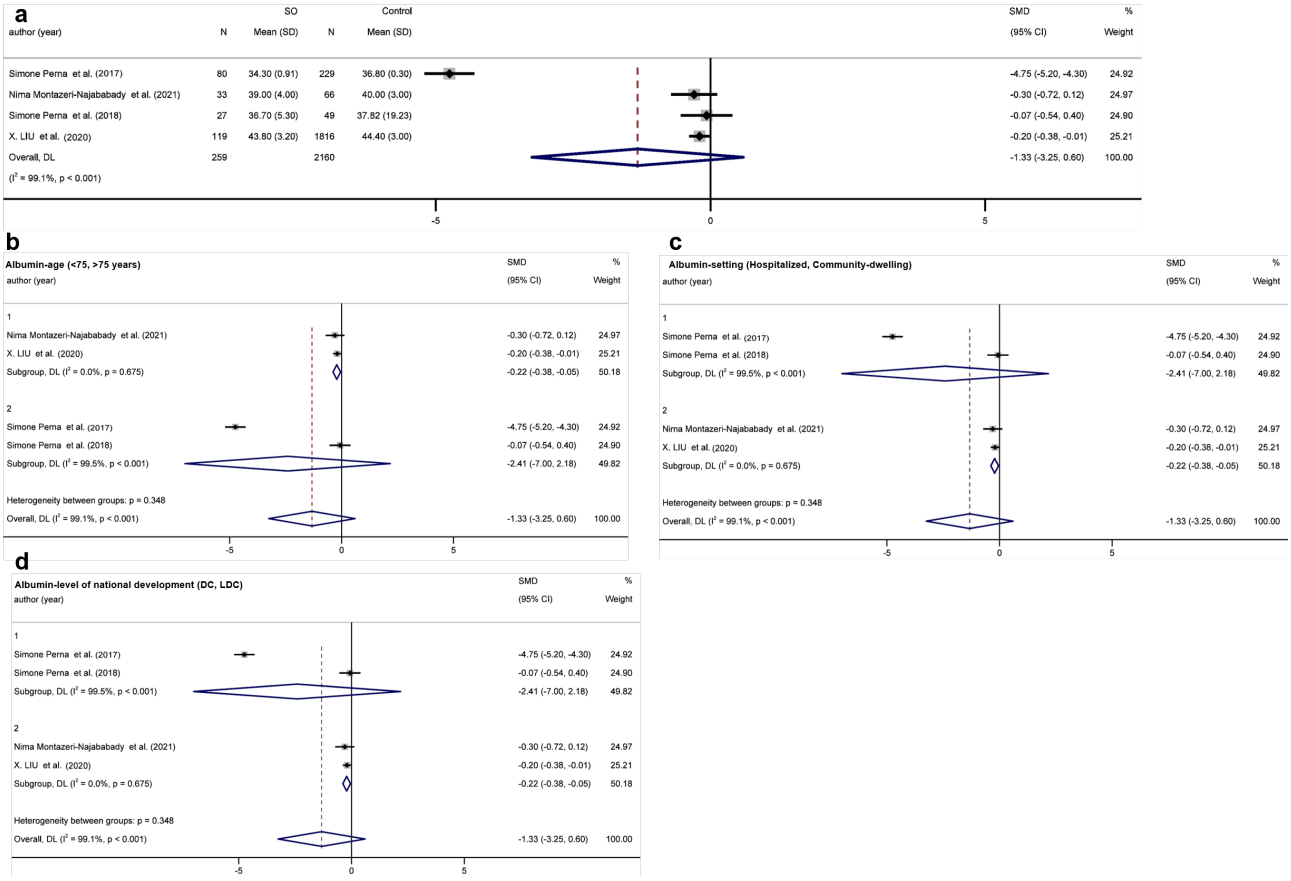

Figure S30: Forest plot of standardized mean differences for ALB in SO using random-effects model. a. Forest plot of ALB in SO; b. Age subgroup; c. Setting subgroup; d. Level of national development subgroup. SMD, standardized mean differences; DC, Developed Countries; LDC, Less Developed Countries.

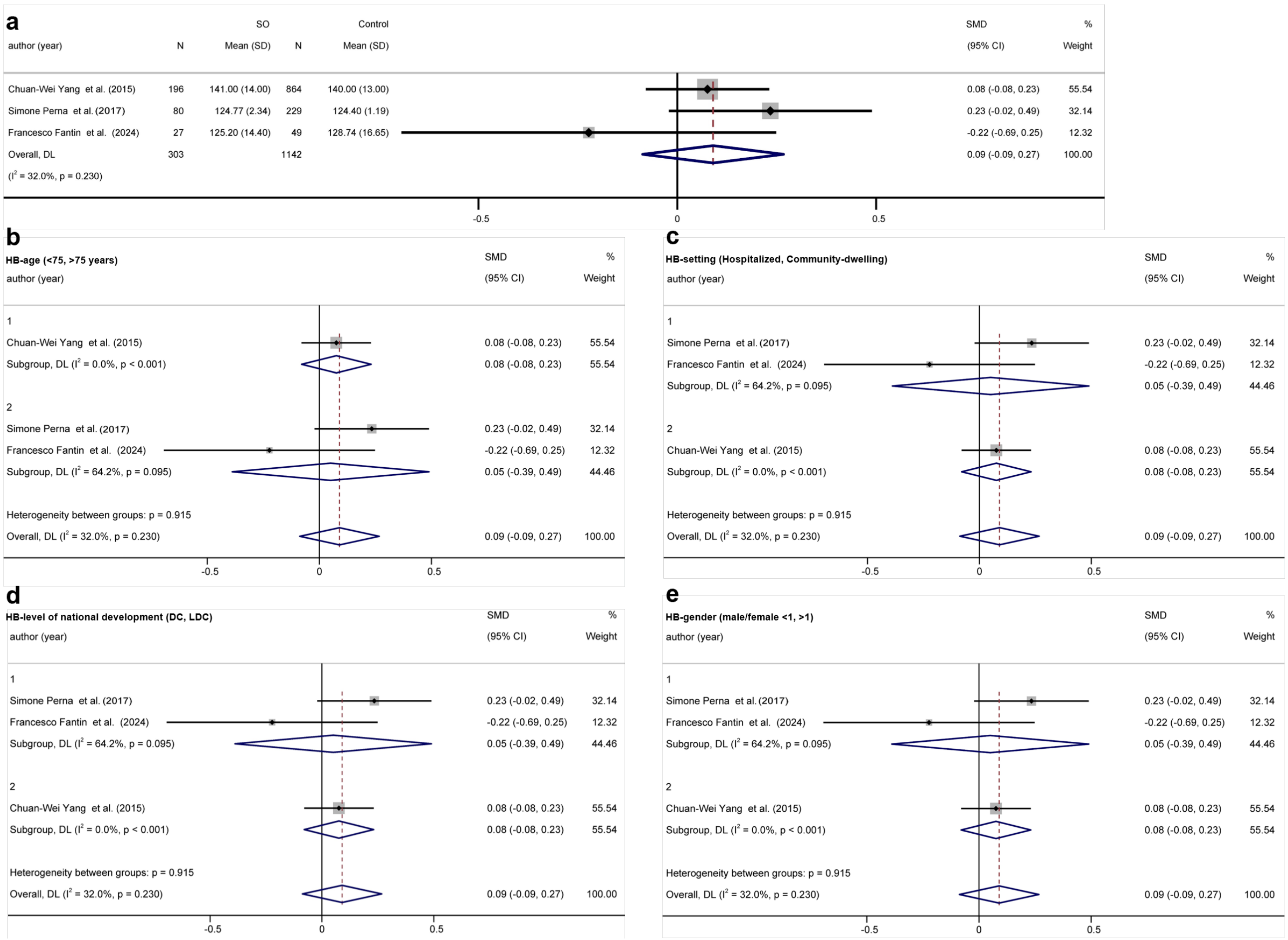

Figure S31: Forest plot of standardized mean differences for HB in SO using random-effects model. a. Forest plot of ALB in SO; b. Age subgroup; c. Setting subgroup; d. Level of national development subgroup; e. Gender subgroup. SMD, standardized mean differences; DC, Developed Countries; LDC, Less Developed Countries.

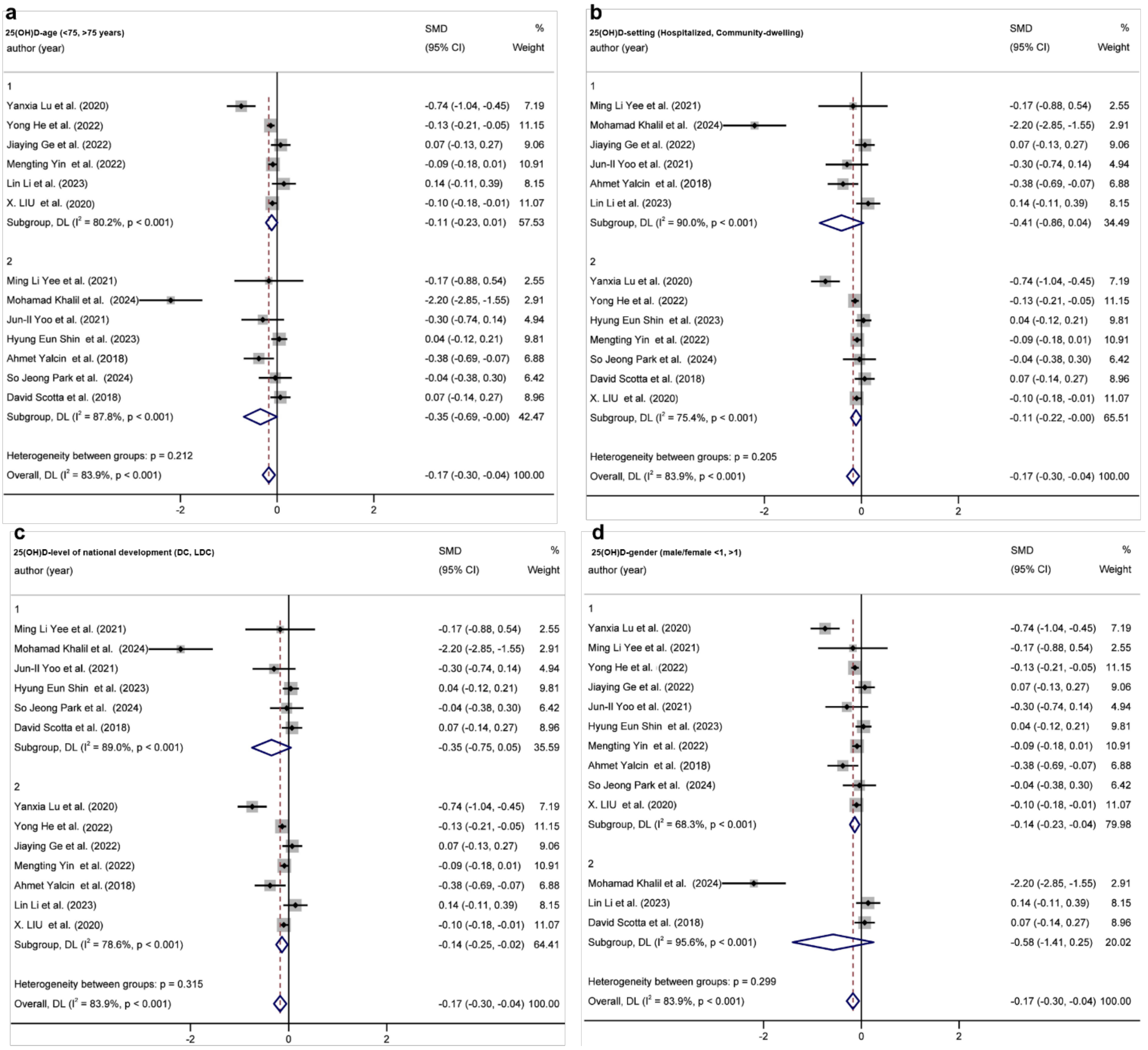

Figure S32: Forest plot of standardized mean differences for 25(OH)D in sarcopenia using random-effects model. a. Age subgroup; b. Setting subgroup; c. Level of national development subgroup; d. Gender subgroup. SMD, standardized mean differences; DC, Developed Countries; LDC, Less Developed Countries.

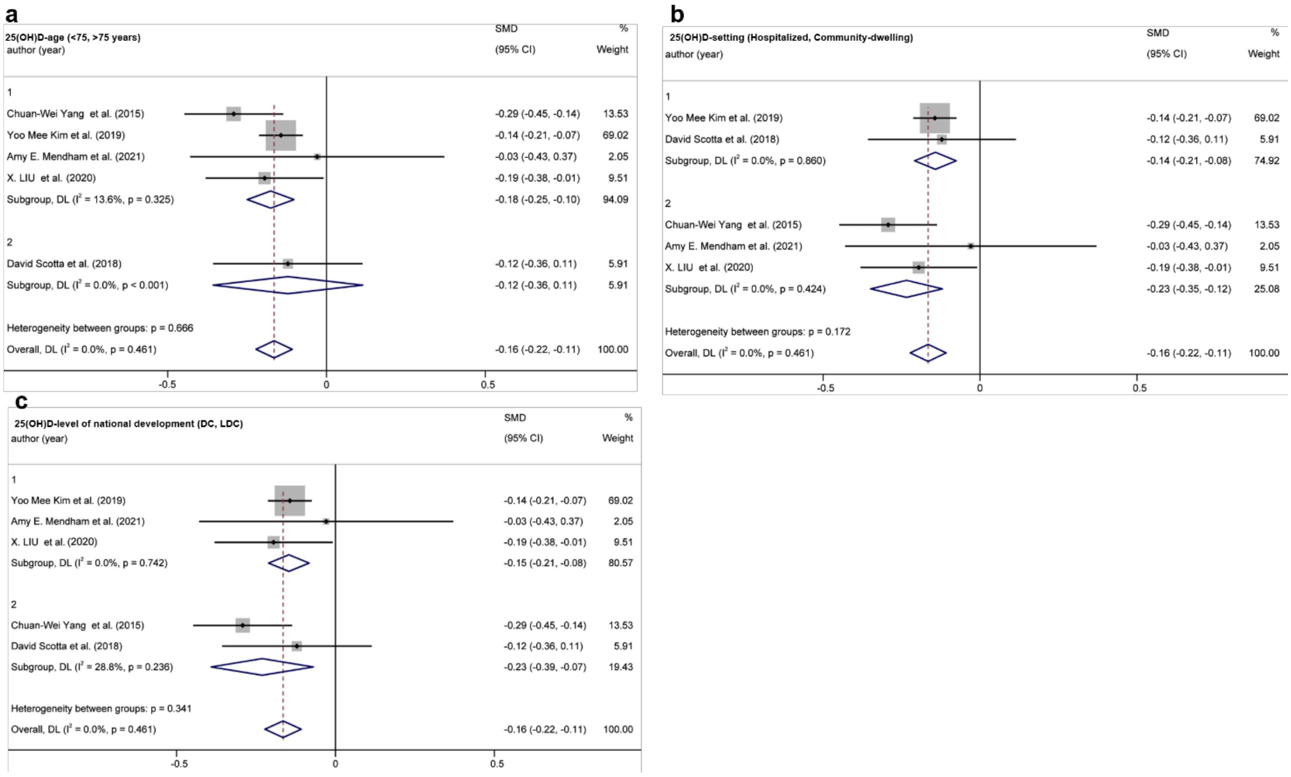

Figure S33: Forest plot of standardized mean differences for 25(OH)D in SO using random-effects model. a. Age subgroup; b. Level of national development subgroup; c. Gender subgroup. SMD, standardized mean differences; DC, Developed Countries; LDC, Less Developed Countries.

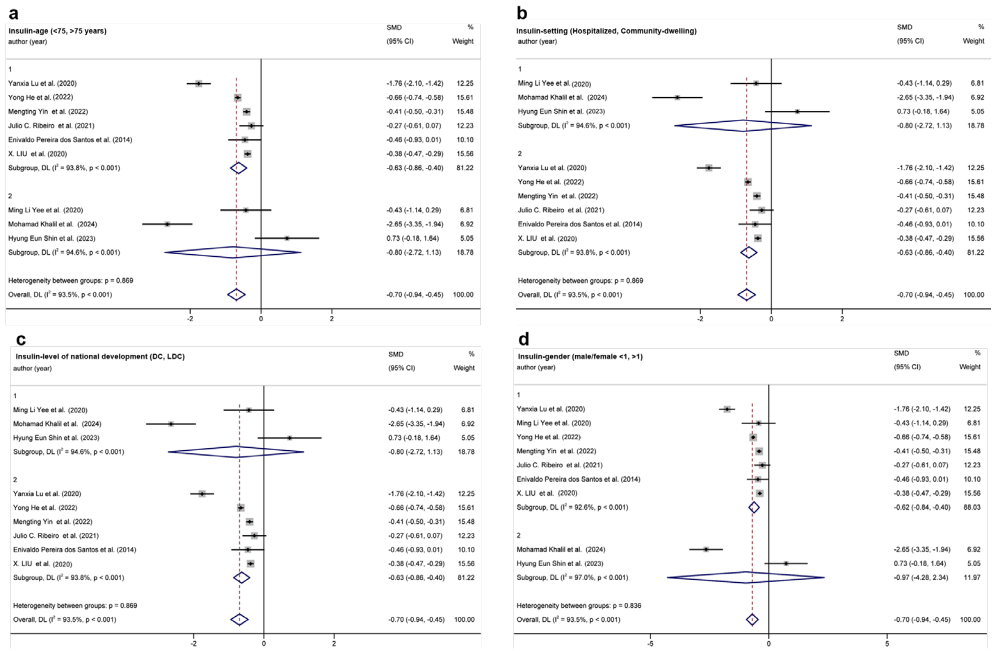

Figure S34: Forest plot of standardized mean differences for insulin in sarcopenia using random-effects model. a. Age subgroup; b. Setting subgroup; c. Level of national development subgroup; d. Gender subgroup. SMD, standardized mean differences; DC, Developed Countries; LDC, Less Developed Countries.

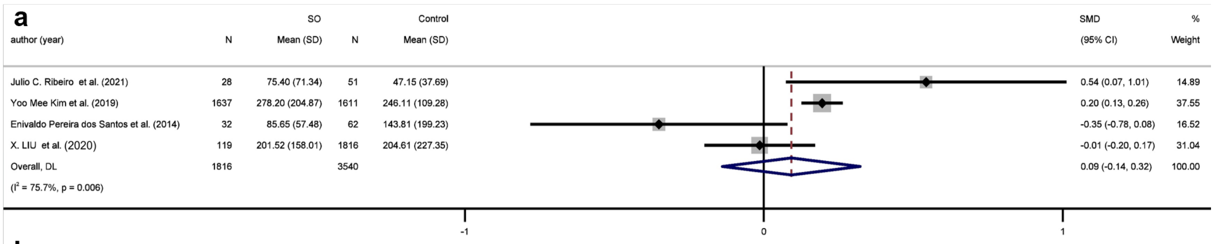

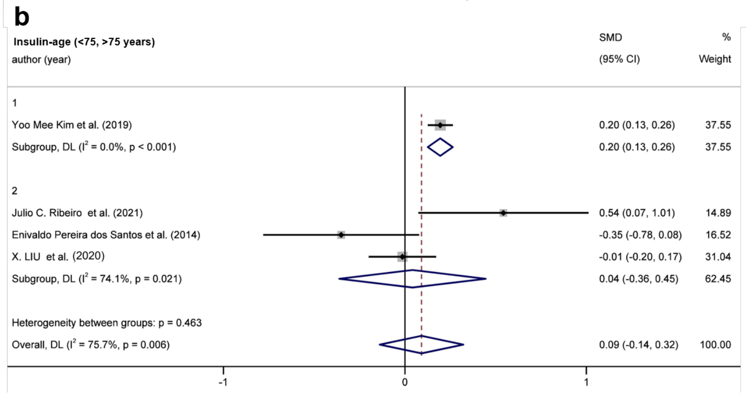

Figure S35: Forest plot of standardized mean differences for insulin in SO using random-effects model. a. Forest plot of insulin in SO; b. Level of national development subgroup; SMD, standardized mean differences; DC, Developed Countries; LDC, Less Developed Countries.

Supplementary S8: Publication bias and Small-study effects

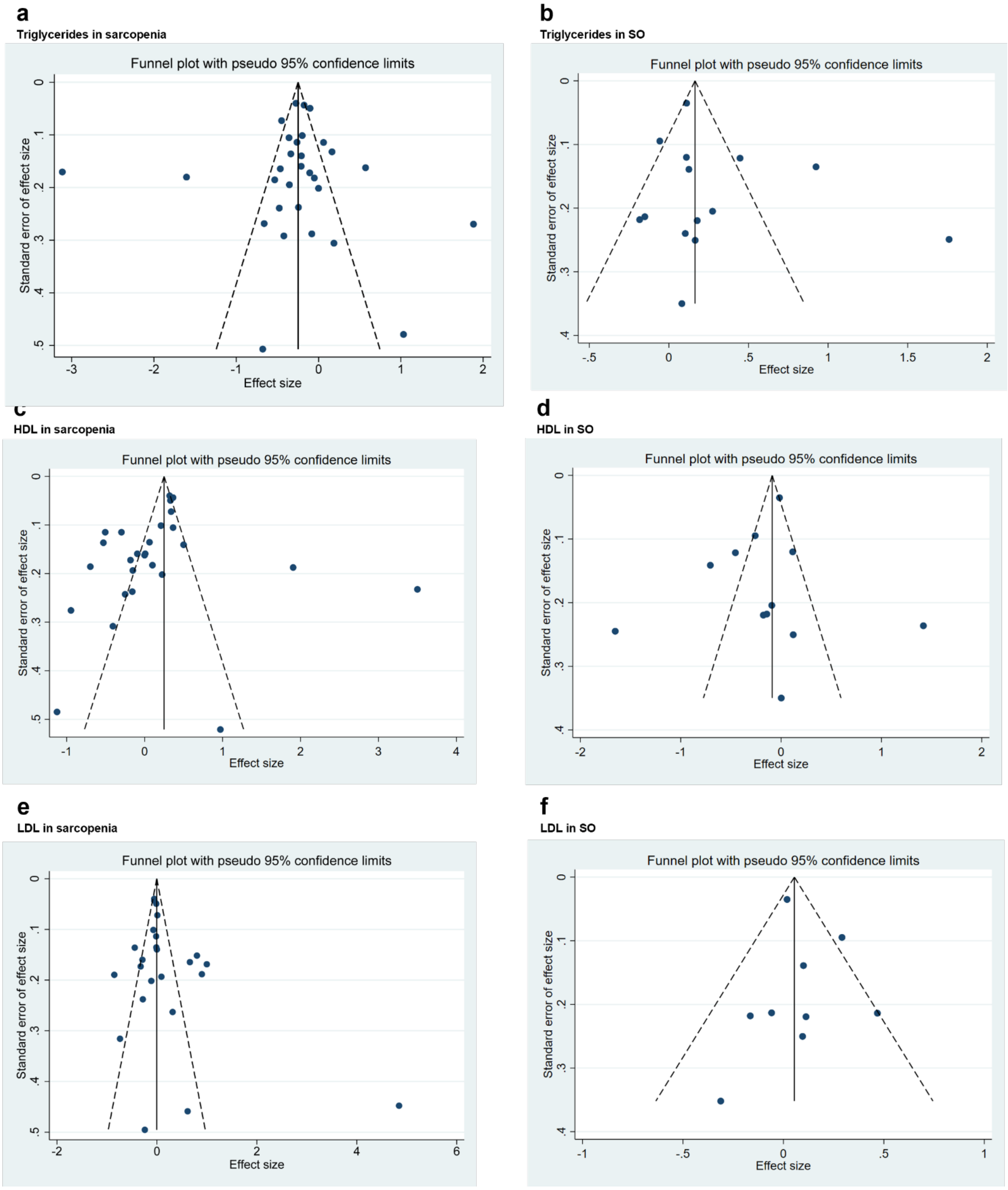

Figure S36: Funnel Plot for Publication Bias Assessment in Sarcopenia and SO Studies. a. Triglycerides in sarcopenia; b. Triglycerides in SO; c. High-density lipoprotein (HDL) in sarcopenia; d. High-density lipoprotein (HDL) in SO; e. Low-density lipoprotein (LDL) in sarcopenia; f. Low-density lipoprotein (LDL)

in SO.

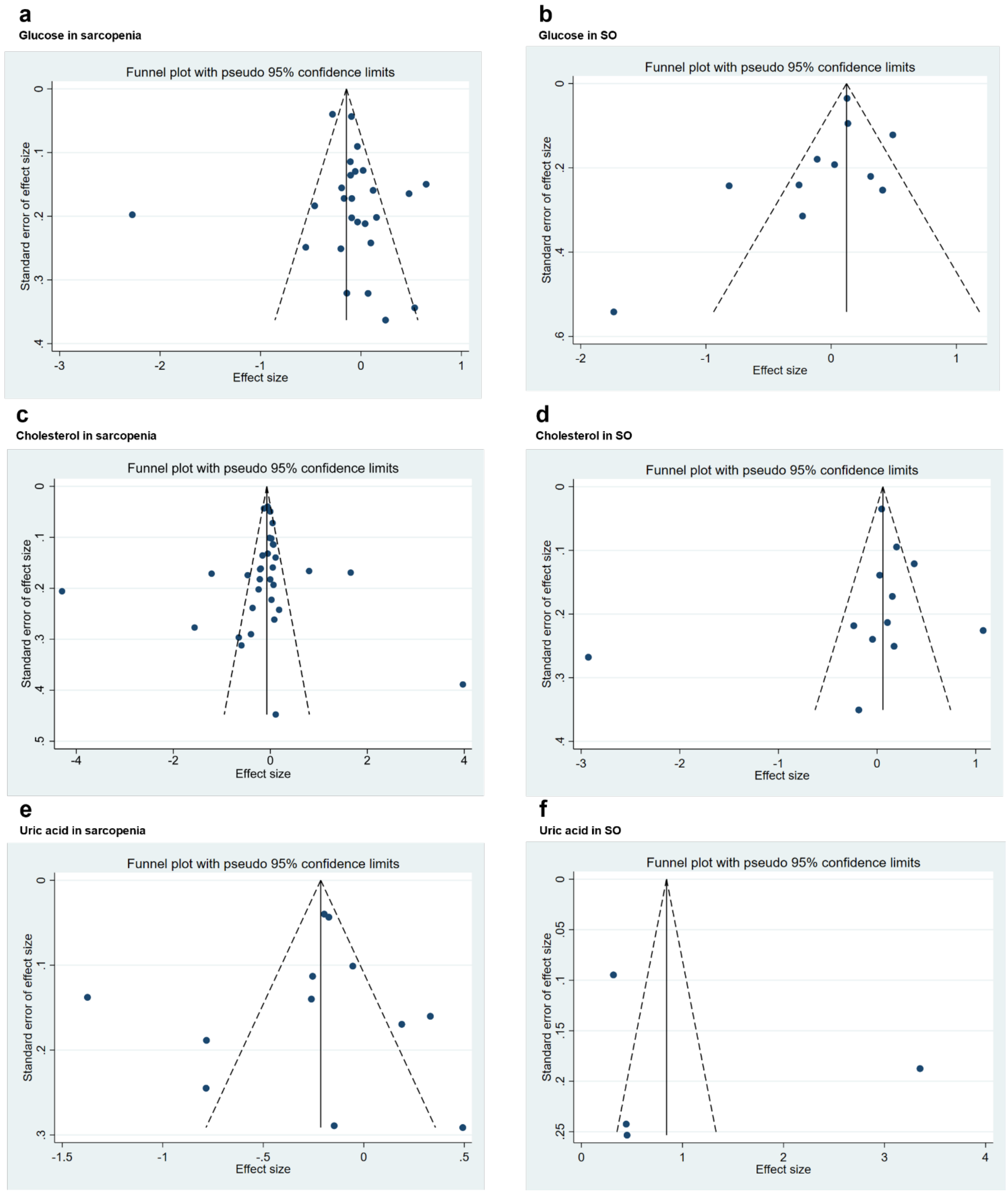

Figure S37: Funnel Plot for Publication Bias Assessment in Sarcopenia and SO Studies. a. Glucose in sarcopenia; b. Glucose in SO; c. Cholesterol in sarcopenia; d. Cholesterol in SO; e. Uric acid lipoprotein (LDL) in sarcopenia; f. Uric acid in SO.

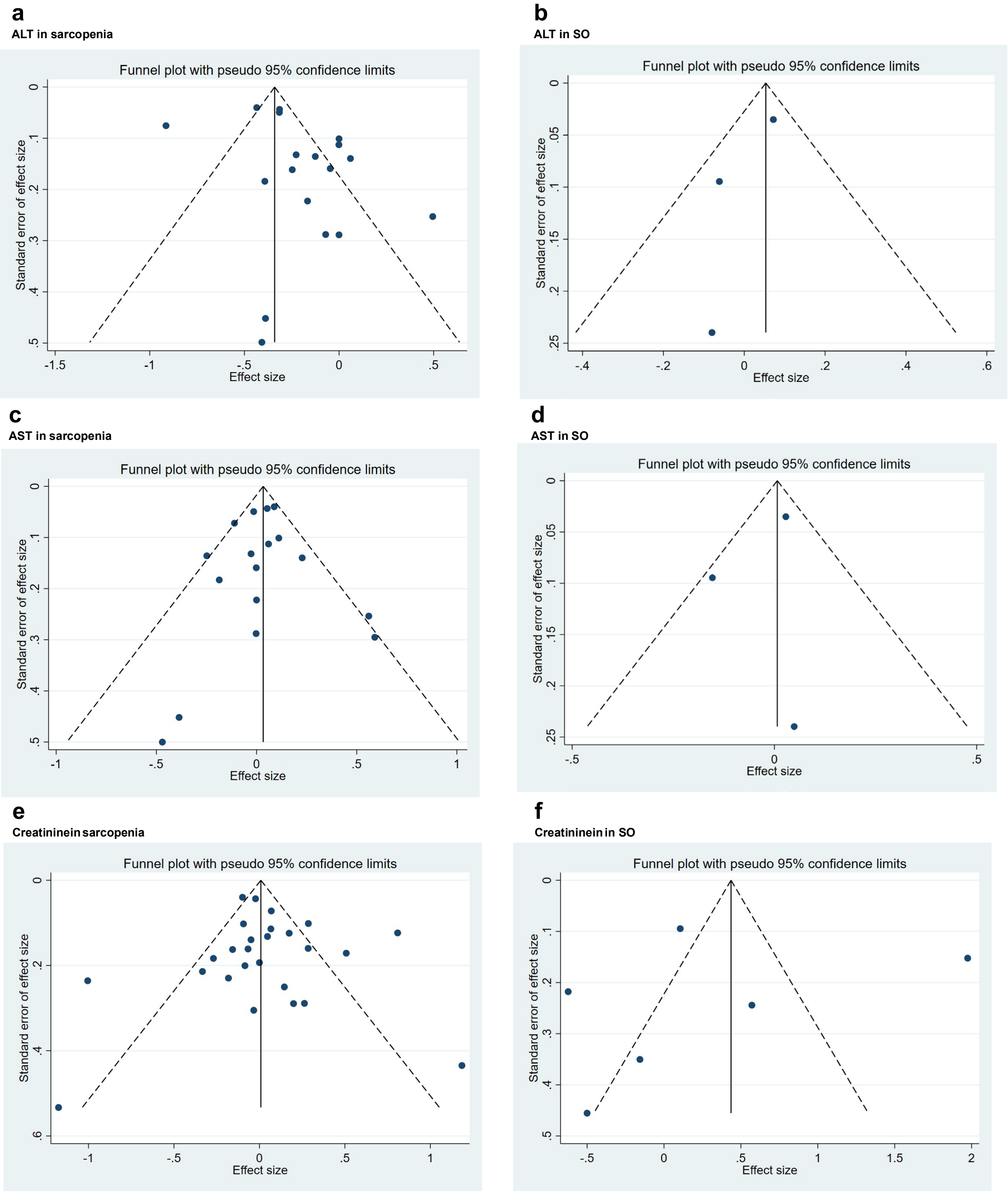

Figure S38: Funnel Plot for Publication Bias Assessment in Sarcopenia and SO Studies. a. Albuminous transaminase (ALT) in sarcopenia; b. Albuminous transaminase (ALT) in SO; c. Glutamate transaminase (AST) in sarcopenia; d. Glutamate transaminase (AST) in SO; e. Creatinine lipoprotein in sarcopenia; f. Creatinine in SO.

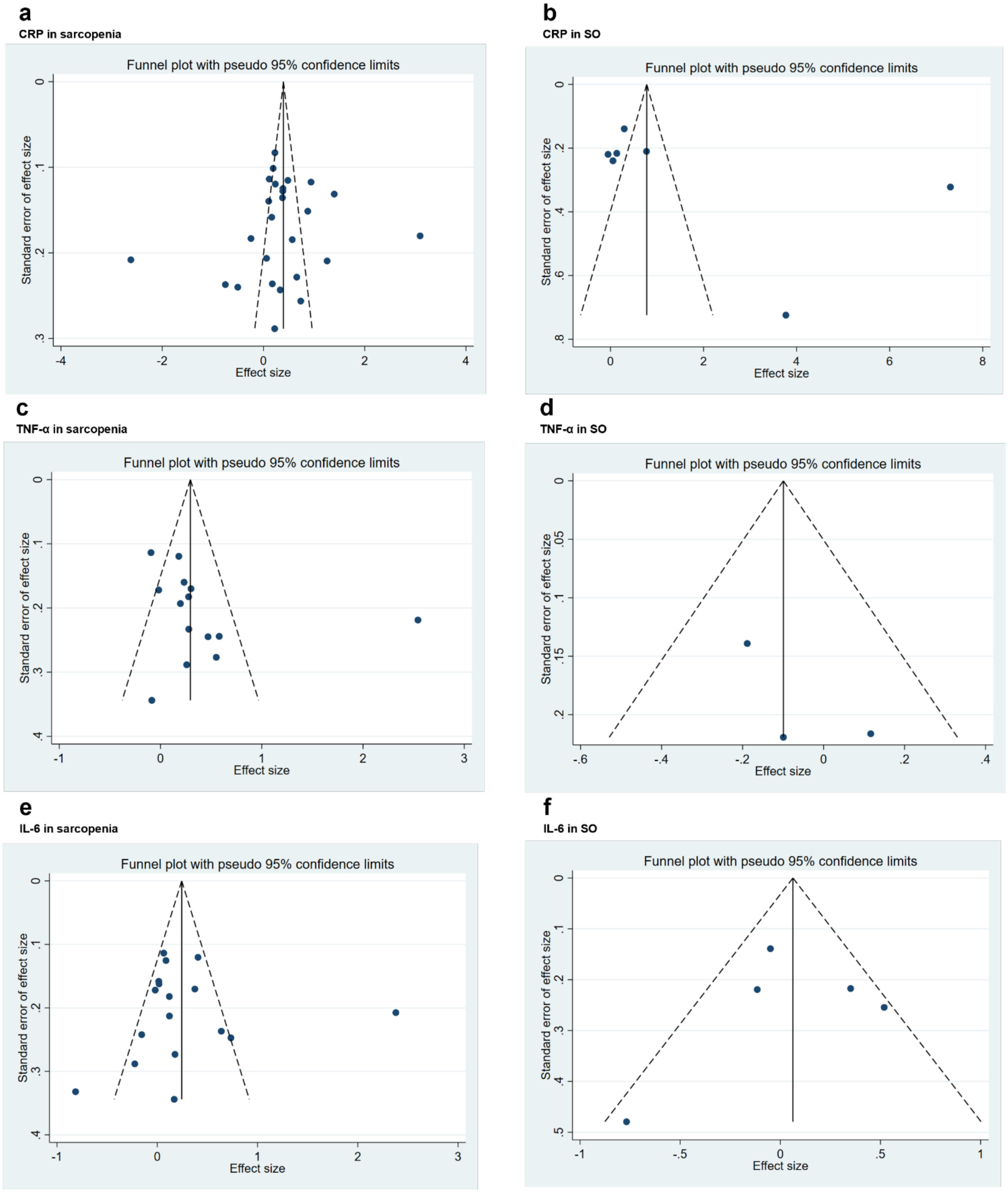

Figure S39: Funnel Plot for Publication Bias Assessment in Sarcopenia and SO Studies. a. C-reactive protein (CRP) in sarcopenia; b. C-reactive protein (CRP) in SO; c. TNF-α in sarcopenia; d. TNF-α in SO; e. IL-6 in sarcopenia; f. IL-6 in SO.

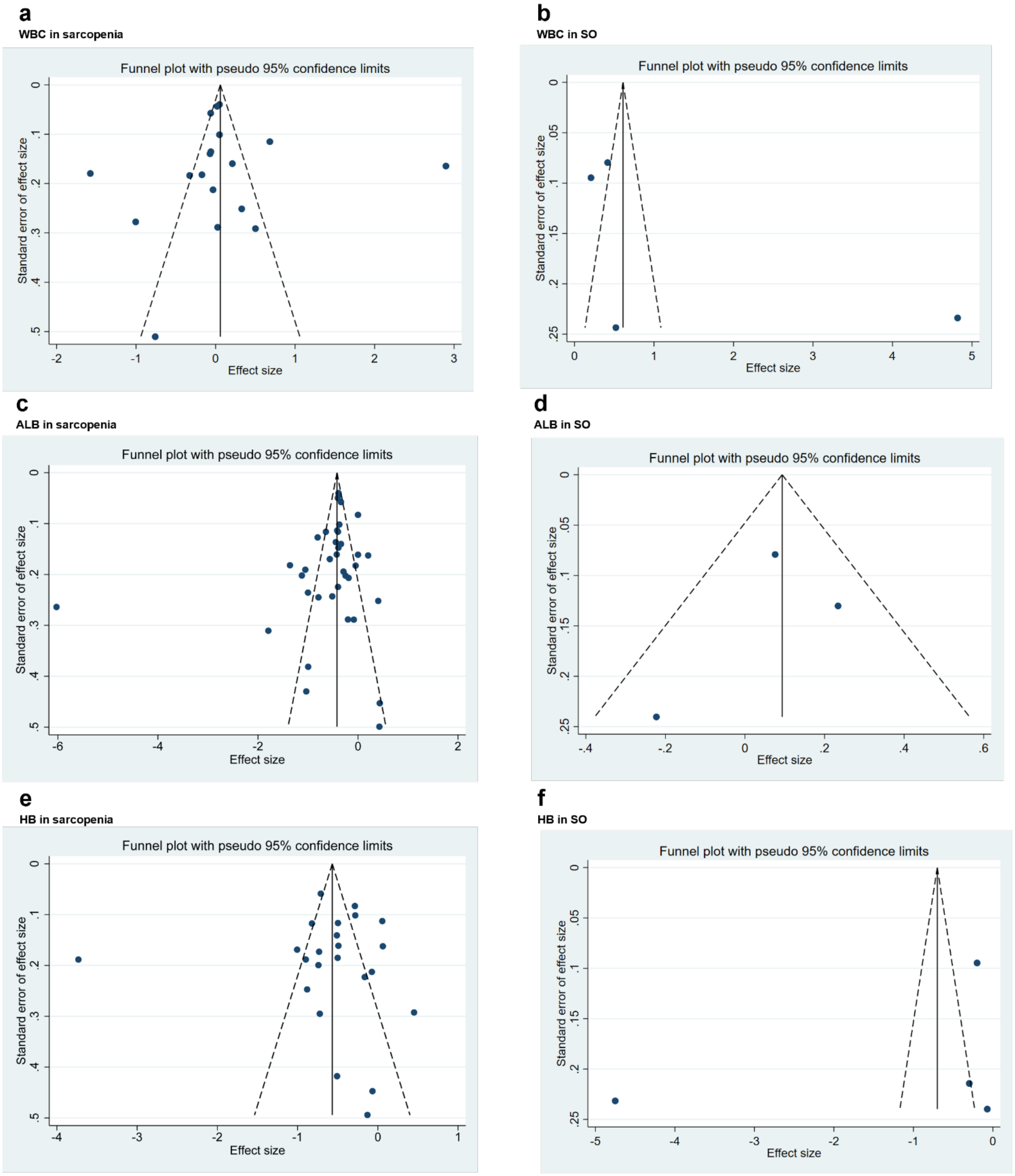

Figure S40: Funnel Plot for Publication Bias Assessment in Sarcopenia and SO Studies. a. White blood cell (WBC) in sarcopenia; b. White blood cell (WBC) in SO; c. Albumin in sarcopenia; d. Albumin in SO; e. Hemoglobin (HB) in sarcopenia; f. Hemoglobin (HB) in SO.

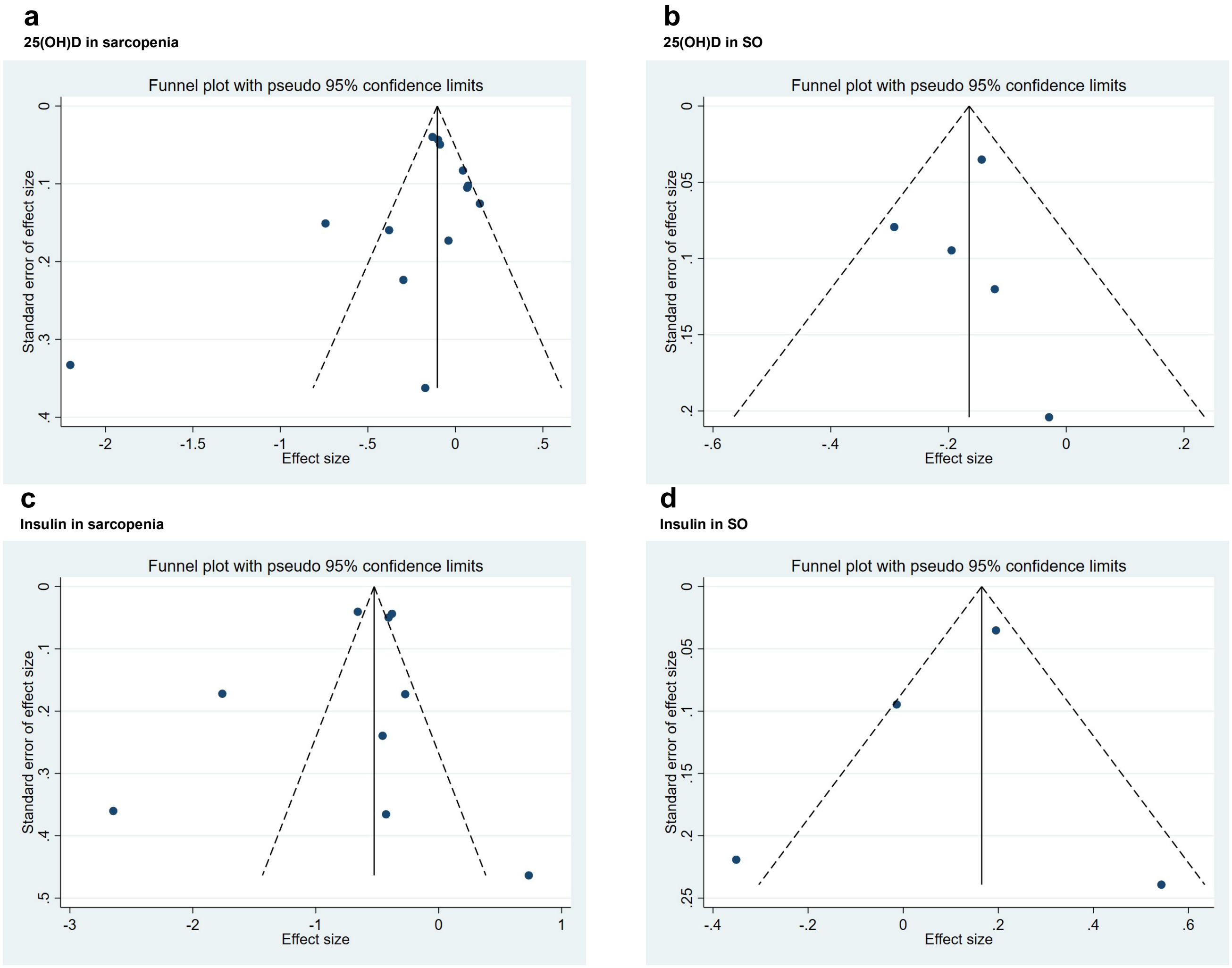

Figure S41: Funnel Plot for Publication Bias Assessment in Sarcopenia and SO Studies. a. 25-hydroxyvitamin D (25(OH)D) in sarcopenia; b. 25-hydroxyvitamin D (25(OH)D) in SO; c. Insulin in sarcopenia; d. Insulin in SO.
